# Polyfunctionalization of vicinal carbon centers and synthesis of unsymmetric 1,2,3,4-tetracarbonyl compounds

**DOI:** 10.1038/s41467-023-36757-w

**Published:** 2023-02-27

**Authors:** Luca De Angelis, Chao Pei, Ana L. Narro, Daniel Wherritt, Rene M. Koenigs, Michael P. Doyle

**Affiliations:** 1 Department of Chemistry, The University of Texas at San Antonio, San Antonio, TX USA; 2 RWTH Aachen University, Institute of Organic Chemistry, Landoltweg 1, 52074 Aachen, Germany

**Keywords:** Synthetic chemistry methodology, Reaction mechanisms

## Abstract

The synthesis and characterization of organic compounds with unusual atom or functional group connectivity is one of the main driving forces in the discovery of new synthetic methods that has raised the interest of chemists for many years. Polycarbonyl compounds are such compounds wherein multiple carbonyl groups are directly juxtaposed and influence each other’s chemical reactivity. While 1,2-dicarbonyl or 1,2,3-tricarbonyl compounds are well-known in organic chemistry, the 1,2,3,4-tetracarbonyl motif remains barely explored. Herein, we report on the synthesis of such 1,2,3,4-tetracarbonyl compounds employing a synthetic strategy that involves C-nitrosation of enoldiazoacetates, while the diazo functional group remains untouched. This strategy not only leverages the synthesis of 1,2,3,4-tetracarbonyl compounds to an unprecedented level, it also accomplishes the synthesis of 1,2,3,4-tetracarbonyl compounds, wherein each carbonyl group is orthogonally masked. Combined experimental and theoretical studies provide an understanding of the reaction mechanism and rationalize the formation of such 1,2,3,4-tetracarbonyl compounds.

## Abstract (web summary)

The synthesis of polycarbonyl compounds featuring four or more carbonyl groups is a synthetic challenge for organic chemists. Here, the authors describe how unsymmetric polycarbonyl compounds bearing up to 4 adjacent carbonyl groups can be accessed within a two-step synthesis from readily available starting materials and discuss their reactivity.

## Introduction

**Fig. 1:**
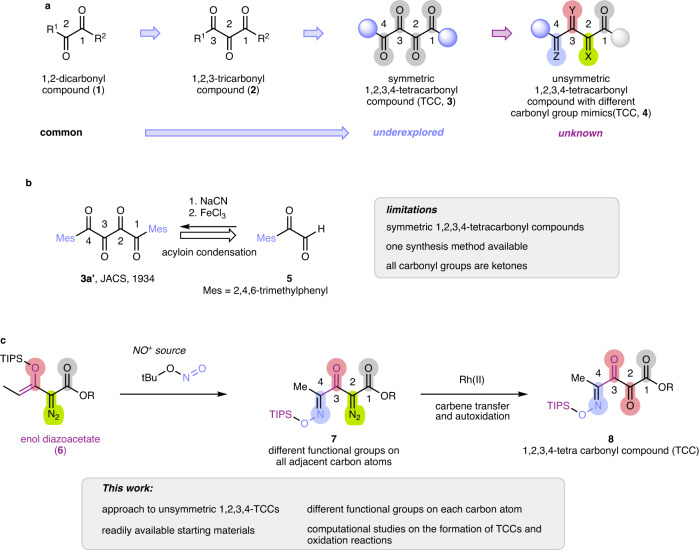
Synthesis of 1,2,3,4-tetracarbonyl compounds. **a** Polycarbonyl compounds. **b** Conventional synthesis of 1,2,3,4-tetracarbonyl compounds. **c** This work: reaction of enoldiazoacetates in the synthesis of unsymmetric 1,2,3,4-tetracarbonyl compounds.

The questions of how many carbonyl groups can be juxtaposed in a cyclic or acyclic compound and what are their unique properties and reactions are of continuing interest (Fig. 1a)^1–3^. Each adjacent carbonyl of a polycarbonyl compound modifies its neighbor and provides different reactivities and selectivities in its reactions. For example, pioneering work by Wasserman and coworkers^4^ demonstrated wide applications of vicinal tricarbonyl compounds (VTCs, **2**) in the synthesis of natural products and synthetic intermediates^5^, and numerous examples have been reported that utilize the high electrophilicity of the central carbonyl of VTCs in diastereoselective and enantioselective nucleophilic addition reactions^6–9^. A close derivative of VTCs are diazodicarbonyl compounds, where the central carbonyl group is replaced by a diazo functional group. In this case, the central diazo functional group is stabilized by both adjacent electron-withdrawing groups, and their reactivity towards electrophiles (with diazodicarbonyl compounds) or nucleophiles (with metal carbenes) is diminished^10^. The formal introduction of another carbonyl group to VTCs gives a vicinal tetracarbonyl compound (TCC, **3**), which represents an intriguing but highly underdeveloped class of organic compounds. An early study on their synthesis dates back to a report by Gray and Fuson from 1934 on the synthesis of dimesityl tetraketone **3a** via acyloin condensation of mesityl glyoxal (Fig. 1b)^11^. Since then few efforts have been devoted to the synthesis of TCCs, yet even today only limited examples of symmetric TCCs have been described. The propensity of TCCs for nucleophilic addition reactions has been recognized^1, 3^, but applications are strictly limited;^12^ diazo analogs or examples with orthogonal masking of carbonyl groups are non-existent (Fig. 1c). As such, methods that would facilitate a more generalized synthesis of unsymmetric TCCs—ideally bearing complementary masking of each carbonyl group—are in high demand to access and to study the chemical properties of these building blocks.

To achieve this goal, we hypothesized that an appropriately masked 1,2,3-tricarbonyl compound could be used to introduce the fourth carbonyl group, thereby achieving a modular synthesis of unsymmetric 1,2,3,4-tetracarbonyl compounds. Specifically, we considered 3-silyloxyvinyldiazoacetate as a suitable starting point due to its ease of accessibility and the presence of 3 carbonyl groups, which are present as an ester, diazo, and enolether functional group. The latter could then serve as an entry point to introduce the desired fourth carbonyl group by reaction with *tert*-butyl nitrite (TBN), a potent donor of the nitrosonium ion^13, 14^. At the same time, we anticipated the diazo functional group to remain intact thereby opening up a plethora of potential downstream transformations via carbene transfer reactions. As a net result, this strategy would allow the synthesis of a 1-ester-2-diazo-3-keto-4-oxime tetracarbonyl compound, which we considered to be readily convertible to 1-ester-2,3-diketo-4-oxime-containing tetracarbonyl compounds via metal-catalyzed carbene transfer reactions with water followed by oxidation.

Herein, we report a detailed study, on the reaction 3-silyloxyvinyldiazoacetate **6** with TBN, which gives facile access to the desired substrate class of tetracarbonyl compounds **7** (Fig. 1c) via nucleophilic addition and 1,2-silyl migration. Importantly, this approach now allows the synthesis of 1,2,3,4-tetracarbonyl compounds, where all 4 adjacent carbonyl groups bear different functionalities. We study this transformation with a range of different diazo compounds and show potential applications and limitations of the present method. Computational studies are conducted to gain an understanding in the exclusive formation of 1-ester-2-diazo-3-keto-4-oxime tetracarbonyl compounds and to rationalize the reaction mechanism. We conclude with applications of these compounds in carbene transfer reactions, which allows the introduction of protecting groups or the conversion into 1-ester-2,3-diketo-4-oxime functionalities.

## Results and discussion

### Synthesis of 1,2,3,4-tetracarbonyl compounds

**Fig. 2:**
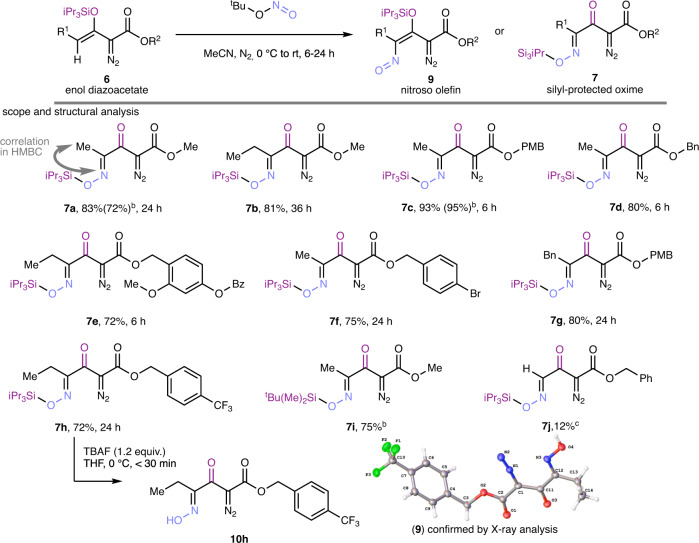
Formation of vicinal 1-ester-2-diazo-3-keto-4-oximes from β-siloxy-α-diazovinylacetates with *tert*-butyl nitrite. **a**. Investigations on the applicability and structural confirmation, desilylation with TBAF and x-ray diffraction. Reaction conditions: ^t^BuONO (3.5 equiv.) was added to a solution containing **6** (0.4 mmol) dropwise at 0 °C. The reaction was continued for the indicated time at room temperature. Another 1.0 equiv. of TBN was added after 24 h. ^b^Yield from reaction performed on a 1.00-g scale in parenthesis. ^c^82% of desilylated benzyl-2-diazo-3-oxobutanoate was isolated.

To assess our hypothesis, we initially studied the reaction between *Z*-configured silyl-protected enoldiazoacetates ***Z*−6** (R^1^ = Me, R^2^ = p-CF_3_C_6_H_4_CH_2_) and TBN in halocarbon solvents, but only unsatisfactory yields of the reaction product were obtained. However, when performed in acetonitrile, only a single product was formed in high yield for different ester functional groups and alkyl substituents R_2_ bound to the enolether with the diazo functional group remaining untouched (Fig. 2). Proton and carbon spectra, as well as HRMS, suggested the selective formation of either the *E*- or *Z*- stereoisomer of nitroso olefin (**9**) or a silyl-protected oxime (**7**). For an unambiguous structural assignment, we performed ^15^N-^1^H HMBC correlation spectroscopy that showed coupling at 390 ppm, which is in the range of an oxime (^15^N chemical shift: 300–400 ppm) but not a C-nitroso (^15^N chemical shift: 800–900 ppm) functional group^15^. HMBC data thus suggests the formation of silyl-protected oxime (**7**). To determine the scope of this reaction, we varied both the gamma substituent of the 3-silyloxyvinyldiazo ester and its ester alkyl group in reactions performed by the addition of excess TBN at 0 °C, then warmed to room temperature. Product yields range from 62–95% (Fig. 2), and reaction times showed a significant dependence on the ester alkyl group. Gamma ethyl and benzyl substituted vinyl diazoacetates **6** react at the same rates as the 3-silyloxyvinyldiazoacetates with a methyl substituent, but benzyl esters having an electron-donating substituent reacted faster. To further demonstrate the generality of this method, the *tert*-butyl(dimethyl)silyl (TBS) group as a protective group formed the oxime-OTBS-protected diazo compound **7i** in high yield. A limitation lies with hydrogen as the gamma substituent; here only significantly reduced yields of **7j** were obtained.

Further evidence for the formation of **7** was then found in a desilylation reaction with tetra-*n*-butylammonium fluoride (TBAF), which gave oxime **10** **h**. The structure of the latter was confirmed by x-ray analysis (Fig. 2) and now provides solid evidence for an intriguing example of a 1,2,3,4-tetracarbonyl compound, where all 4 carbonyl groups bear different functionalities; namely oxime, ketone, diazo, and ester functional groups.

### Studies on the reaction mechanism

**Fig. 3:**
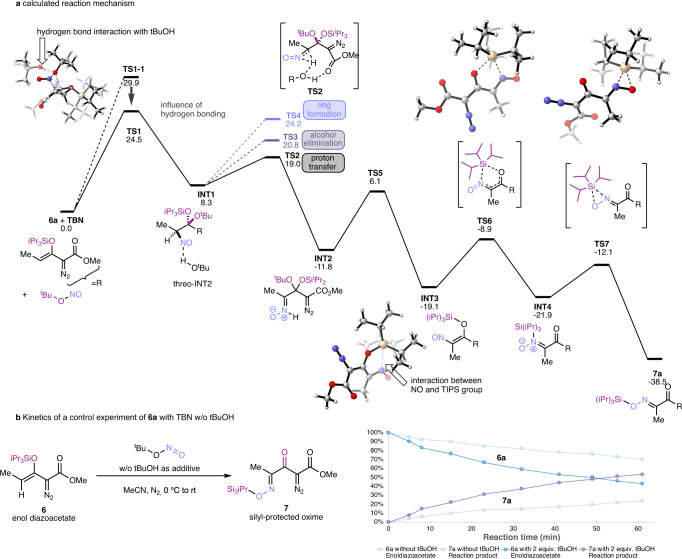
Computational studies on the reaction mechanism. **a** Calculated reaction mechanism. **b** Reaction kinetics.

To better understand the formation of tetracarbonyl compound **7**, we performed theoretical calculations at the (SMD = acetonitrile)-BP86-D3(BJ)/6–311 + +G(d,p)//BP86-D3(BJ)/6–31 G(d) level of theory, which has proven to be a suitable method in a previous study of a similar transformation, Fig. 3a^16^.

We initiated calculation with the additional step of TBN onto the enol group of **6a**. All attempts in locating a first transition state led us to a 1,2-addition transition state. This 1,2-addition step (**TS1-1**, ΔG = 29.9 kcal/mol, Supplementary Fig. 10) can be accelerated by the hydrogen bonding with additional *t*BuOH, which is consistent with experimental results and denoted as **TS1** with an activation energy of 24.5 kcal/mol to give intermediate **INT1**. To verify the computed influence of *t*BuOH, we examined the reaction of enoldiazoacetate **6a** with TBN with and without additional *t*BuOH. Indeed, a deceleration of the reaction was observed in the absence of *t*BuOH, which gave the reaction essentially a 40–60% yield after a 3–4 h reaction time, while in the presence of *t*BuOH, the reaction rapidly went to completion (80–90% yield) within the same time frame (Fig. 3b and Supplementary Figs. 3 and 4). Further calculation data concerned other possible pathways in the first step, such as [5 + 1] cycloaddition via **TS1-2**, metathesis-like process via **TS1-3** and [5 + 2] cycloaddition via **TS1-4**, yet in all cases higher energy barriers (ΔG > 28 kcal/mol in Supplementary Fig. 12) were observed and are thus unlikely to account for the product formation.

From enoldiazoacetate-derived intermediate **INT1**, we analyzed a set of downstream pathways, which could account for the reaction outcome. We could identify a favorable proton transfer process through **TS2** (ΔG^‡^ = 10.7 kcal/mol). We rationalize that the acetal group and NO group enhance the acidity of their α-hydrogen, which is required to promote this proton transfer step or direct *t*BuOH elimination via **TS2** (ΔG^‡^ = 10.7 v.s. 12.5 kcal/mol for **TS3**)^17^. Importantly, in the case of enoldiazoacetate, a potential ring closing step through **TS4** has a substantially higher energy barrier of 15.9 kcal/mol, which we reasoned to be due to reduced conformational flexibility caused by the methyl group. As a consequence, the formation of precursors that can lead to 1,2,3-triazine 1-oxides from **6a** is less favored^14^. **INT2** then undergoes elimination of *t*BuOH via **TS5** and leads to the formation of a *Z*-nitroso silylenol ether **INT3**, which exhibits an ideally suited geometric orientation of the nitroso and the enolether group for subsequent silyl migration. Analysis of the structure of **INT3** evidences a rather short N-Si distance of 2.57 Å, which indicates that the intermediate **INT3** has a strong tetrel bonding interaction between N and Si atoms^18^.

We next examined the formation of the silyl-protected oxime **7a**. Intramolecular silyl group transfer between oxygen atoms is a known process, but with limited examples^11^, and none of the kind proposed in Fig. 3a to explain the formation of **7**. The tetrel interaction between N and Si in **INT3** facilitates the intramolecular silyl migration step via the five-membered-ring transition state **TS6**. Finally, facile cis-migration of the silyl group via **TS7** leads to the TIPS-protected (*E*)-oxime product **7a**, which is the driving force that directs the initial vinylogous addition product from 3-silyloxyvinyldiazoacetates to irreversibly form **6**. As part of these studies, we have also considered a one-step O-O 1,5-silyl migration process, which is unfavorable due to its high activation energy of 19.5 kcal/mol and leads to an undesired TIPS-protected (*Z*)-oxime product (Supplementary Fig. 11).

### Applications of 1-ester-2-diazo-3-keto-4-oxime tetracarbonyl compounds

**Fig. 4:**
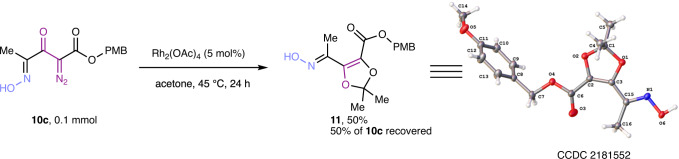
Carbene transfer reaction of oxime 10c. Rhodium-catalyzed reaction of unprotected oxime with acetone and X-ray crystal structure.

As anticipated from a diazo group between two carbonyls^19^, catalytic dinitrogen extrusion from **7** or **10** with either dirhodium tetraacetate or copper(I) catalysts does not occur at room temperature. However, treatment of **10c** at a modestly higher temperature with rhodium acetate in acetone formed the adduct **11** expected from carbonyl ylide formation and carbonyl-induced ring closure in modest yield (Fig. 4) with 50% of reactant **10c** recovered^20^. Interestingly, the same reaction performed with the TIPS-protected **7c** under the same conditions did not produce the corresponding acetone adduct, and 90% of the reactant **7c** was recovered.

**Fig. 5:**
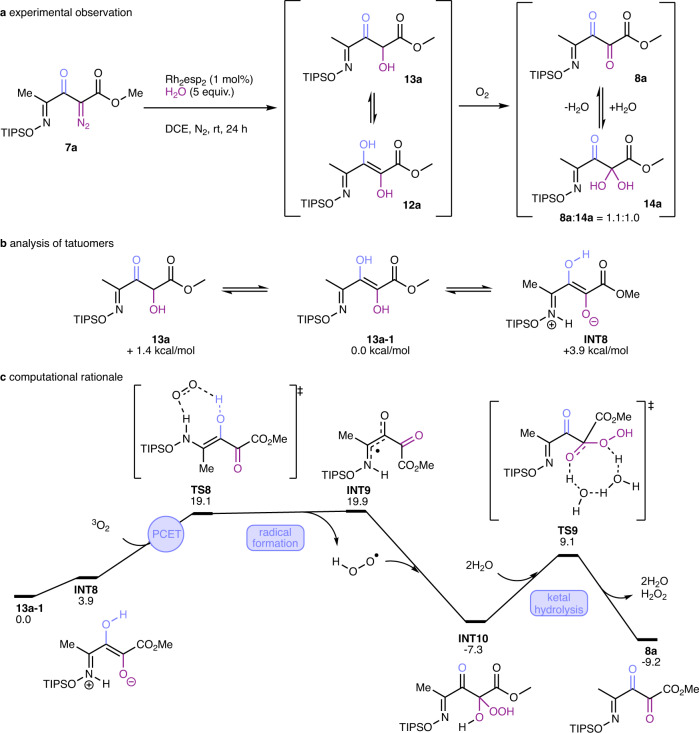
Reaction of a silyl-protected 1,2,3,4-tetracarbonyl compound in a rhodium-catalyzed carbene transfer reaction with water. **a** Experimental observation. **b** Analysis of tautomers. **c** Computational studies.

Treatment of **7a** with Rh_2_(esp)_4_ in 1,2-dichloroethane with added 5 equiv. of water under an atmosphere of air produced a surprising outcome. Instead of the expected alcohol by O-H insertion (**12a**/**13a**), the diazo carbon was converted to a carbonyl group in high yield; and the resulting product mixture was composed of both the keto form (**8a**) and its hydrate (**14a**) in approximately equal amounts (Fig. 5a). Since this process involves overall oxidation, we examined the catalyst by spectrophotometric analysis to determine if its oxidation state changed during the reaction, but we found no change in its oxidation state throughout the course of the reaction compared to the catalytic O-H insertion reaction with ethyl diazoacetate. However, when this reaction was performed under the same conditions but in an inert atmosphere, a new product was detected whose spectroscopic and HRMS analyses were consistent with enediol **12a** which, depending on the solvent employed for analysis, is in equilibrium with its α-hydroxy carbonyl derivative **13a**. Upon exposure to air, enediol **12a** formed the **8a/14a** mixture. Thus, the formation of **8a/14a** is due to oxidation of **12a** by molecular oxygen in a manner similar to, but faster than, ascorbic acid^21, 22^, and suggests that the oxime at the 4-position of **12a** enhances this oxidation. Confirmation of this is found in DFT calculations (Fig. 5b). We found that dienol **12a** undergoes a facile intramolecular proton transfer and leads to **INT8**, which is slightly endergonic. However, **INT8** can readily undergo a proton-coupled electron transfer with triplet oxygen with an activation-free energy of 19.1 kcal/mol. Subsequent radical coupling leads to acetal intermediate **INT10**, which reacts via water-assisted H_2_O_2_ elimination to afford product **8a**. This observation is in line with the electrochemical oxidation potential of enediol **12a** (0.652 V in DMF), which suggests facile oxidation of enediol **12a**.

**Fig. 6:**
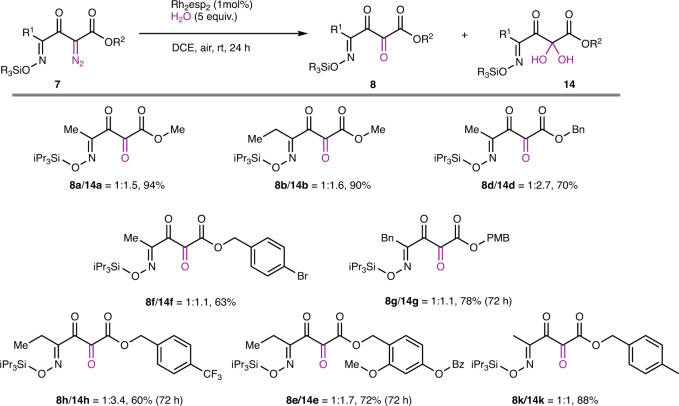
Formation of 1,2,3,4-tetracarbonyl compounds. Rhodium-catalyzed reaction of oximes **7** with water and aerobic oxidation.

The generality of this transformation can be seen in the results obtained with the diazo analogs in Fig. 6. Reactions performed in dichloroethane under an air atmosphere with 5.0 molar equivalents of water catalyzed by Rh_2_(esp)_2_ yielded the 1,2,3-tricarbonyl-4-oxime and its hydrate in modest to excellent yields usually within 24 h.

In summary, we herein report on a streamlined and facile synthetic method of densely functionalized 1,2,3,4-tetracarbonyl compounds, wherein each of the four carbonyl groups bears different functionalities. The reaction of enoldiazoacetates with *tert*-butyl nitrite proceeds via C-alkylation of the enol functional group, without touching the diazo functional group, thereby installing an oxime onto the diazoacetate to give rise to 1-ester-2-diazo-3-keto-4-oximes in excellent yield. This approach not only solves the synthesis problem of 1,2,3,4-tetracarbonyl compounds, it also gives way to unprecedented vicinal tetracarbonyl compounds. Control experiments, and theoretical calculations provide important support of the reaction mechanism of this reaction. The work presented in this article will clearly stimulate further research into the reactivity of 1,2,3,4-tetracarbonyl compounds and allow their application in modern organic synthesis.

## Methods

### Formation of 1-ester-2-diazo-3-keto-4-protected oximes

*tert*-Butyl nitrite (3.5 equiv., 1.4 mmol) was added dropwise over 1 min to a round bottom flask containing a solution of enoldiazo compound **6** (0.4 mmol, 0.1 M in MeCN) at 0 °C under an N_2_ atmosphere. The reaction solution was slowly warmed to room temperature, and the progress of the reaction was followed by TLC until consumption of the enoldiazo compound was complete. The color of the solution went from orange/yellow to colorless. The solvent was then removed under reduced pressure, and the residue was purified by flash chromatography (hexane/EtOAc = 5/1) to give the desired diazo product **7**.

### Formation of 1-ester-2-diazo-3-keto-4-oximes

To a solution of the protected oxime **7** (0.1 mmol, 0.2 M in THF) in a dry 8-mL vial was added TBAF (1.5 equiv., 0.15 mmol) at 0 °C all at once. The progress of the reaction was followed by TLC until consumption of the protected oxime was complete, and the residue was purified by flash chromatography (DCM/MeOH = 9/1) to give the desired product **9**.

### Formation of 1-ester-2,3-diketo-4-oximes

In a dry 8-mL tube, to the solution of Rh_2_(esp)_2_ (1 mol%) in 1 mL of dichloroethane with H_2_O (5.0 equiv., 0.5 mmol), diazo-protected oxime **9** (0.1 mmol,) was added over 1 h with a syringe pump at room temperature. The color of the solution changed from light blue/green to light brown after 24 h. The residue was then purified by flash chromatography (hexane/ethyl acetate = 3/1) to give the desired product as an inseparable mixture of 1-ester-2,3-diketo-4-oximes **13** and their hydrate **14**.

## Supplementary information

Supplementary Information

Description of Additional Supplementary Files

Supplementary Data 1

## Data Availability

The authors declare that data supporting the findings of this study are available within the paper and its supplementary information files. Cartesian coordinates of all stationary points are available in Supplementary Data 1.
