# Supplementary material for: Polyfunctionalization of vicinal carbon centers and synthesis of unsymmetric 1,2,3,4-tetracarbonyl compounds
Source: Nat Commun. 2023 Feb 27;14:1109. doi: 10.1038/s41467-023-36757-w (PMC9971237; doi:10.1038/s41467-023-36757-w)
Supplement: Supplementary file 1 — Supplementary Information [file 41467_2023_36757_MOESM1_ESM.pdf]

Supplementary Information

Polyfunctionalization of Vicinal Carbon Centers and Synthesis of 1,2,3,4-Tetracarbonyl Compounds

Luca De Angelis,[1§] Pei Chao,[2§] Ana L. Narro,[1] Daniel Wherritt,[1] Rene M. Königs,[2*] and Michael P. Doyle[1*]

[1]Department of Chemistry, University of Texas at San Antonio, San Antonio, Texas, USA

[2]Institute of Organic Chemistry, RWTH Aachen University, Aachen, North Rhine-Westphalia, Germany

[*]Email: michael.doyle@utsa.edu

[*]Email: rene.koenigs@rwth-aachen.de

1. Supplementary Methods..........2

1.1 General conditions..........2

1.2 General procedure for the synthesis of enoldiazo compounds..........3

2. Supplementary Discussion..........4

2.1 Optimization of the reaction conditions..........4

2.2 Kinetic Studies..........6

2.3 Oxidation potential studies..........7

2.4 Solvent influence on the equilibrium between α-hydroxy carbonyl (13a) and enediol (12a) compounds..........9

2.5 General procedures..........9

2.6 Analytical and spectral characterization data for products..........10

2.7 Crystallographic data..........17

2.8 Computational Details..........21

2.9 Computed Reaction Pathways..........21

2.10 Computed Energy of all Stationary Points..........24

2.11 Copies of NMR Spectra..........26

3 Supplementary References..........50

1. Supplementary Methods

1.1 General conditions

All reactions, unless noted, were performed in oven-dried (150 °C) glassware with magnetic stirring under an atmosphere of air. Analytical thin layer chromatography (TLC) was carried out using EM Science silica gel 60 F254 plates; visualization was accomplished with UV light (254 nm). Column chromatography was performed on CombiFlash® Rf200 and Rf+ purification systems using normal phase disposable columns. Melting points were obtained uncorrected from an Electro Thermo Mel-Temp DLX 104 device. NMR spectra were recorded on a on a Bruker spectrometer (500 MHz or 300 MHz) and calibrated using the resonance signal of the residual undeuterated solvent for ^{1}H-NMR [δ_H = 7.26 ppm ($CDCl_3$), δ_H = 2.50 ppm ($(CD_3)_2SO$), δ_H = 1.94 ppm CD_3CN, δ_H = 8.03 ppm $DCON(CD_3)_2$] and deuterated solvent for ^{13}C-NMR [δ_C = 77.16 ($CDCl_3$), δ_C = 39.5 $(CD_3)_2SO$, δ_C = 118.3 ppm CD_3CN, δ_C = 163.2 ppm $DCON(CD_3)_2$] as an internal reference at 298 K. Spectra were reported as follows: chemical shift (δ ppm), multiplicity (s = singlet, d = doublet, t = triplet, q = quartet, p = pentet, hept = heptet, m = multiplet, comp = composite), coupling constants (Hz), integration and assignment. ^{13}C-NMR spectra were collected on Bruker instruments (126 MHz and 75 MHz) with complete proton decoupling. ^{15}N-NMR was obtained on Bruker instrument (50.664 MHz) through ^{15}N-^{1}H HMBC NMR. High-resolution mass spectra (HRMS) were performed on a Bruker MicroTOFESI mass spectrometer with an ESI resource using CsI or LTQ ESI positive ion calibration solution as the standard. Tetrahydrofuran, dichloromethane, chloroform, and toluene were purified using a JC-Meyer solvent purification system.

Materials: all the *β*-keto-esters, *tert*-butyl nitrite (TBN: 90% TBN in ^{t}BuOH), tetra-*n*-butylammonium fluoride (TBAF), $Rh_2(OAc)_4$, $Rh_2(esp)_2$, *tert*-butyldimethylsilyl trifluoromethanesulfonate and triisopropylsilyl trifluoromethanesulfonate were purchased from Sigma-Aldrich, Fisher, TCI, or Oakwood Chemicals and were used without further purification. Enoldiazo compounds **6a-6k** were prepared according to the reported literature.[1] A representative procedure for the synthesis of Enoldiazo compounds **6a-6k** is given in this supplementary information.

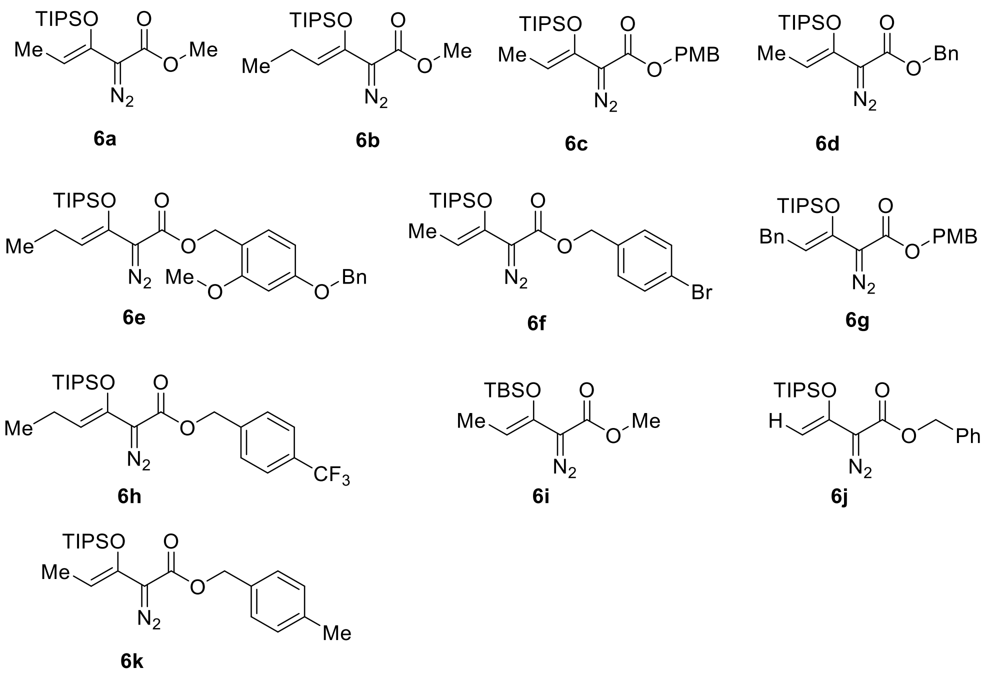

Supplementary Fig. 1. Overview on enoldiazo compounds included in the manuscript.

1.2 General procedure for the synthesis of enoldiazo compounds

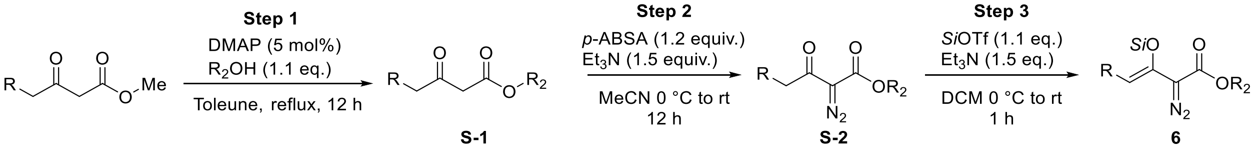

Supplementary Fig. 2. Synthesis of enoldiazo compounds **6**.

Step 1: Following the reported procedure,[1] to a 100-mL oven-dried flask containing a magnetic stirring bar, the ethyl 3-oxobutanoate (10 mmol, 1.16g), the corresponding alcohol (11 mmol, 1.1 eq.) and DMAP (4-dimethylaminopyridine, 0.5 mmol, 5 mol%) were dissolved in toluene (30 mL), and the reaction mixture was stirred at 110-120 ℃ overnight (monitored by TLC until all of the starting material was consumed). After cooling to room temperature and removing the solvent in vacuo, the residue was purified by column chromatography on silica gel using a 10:1 to 4:1 gradient of hexane/ethyl acetate (v/v) as eluent to afford the **S-1** in >80% yield.

Step 2: Following the reported procedure,[1] to a stirred solution of an oxobutanamide **S-1** (10 mmol, 1.0 equiv.) and *p*-acetamidobenzenesulfonyl azide (*p*-ABSA), (12 mmol, 1.2 equiv.) in acetonitrile (5 ml/mmol), triethylamine (15 mmol, 1.5 equiv.) was added dropwise at 0 ℃ over 3 min. The reaction mixture was allowed to warm to room temperature and stirred for 12 h. Acetonitrile was then removed under reduced pressure, and the residue was redissolved in dichloromethane. The sulfonamide precipitate was filtered, and the filtrate was concentrated under reduced pressure. The residue was then purified by column chromatography on silica gel using a 9:1 to 4:1 gradient of hexane/ethyl acetate (v/v) as eluent to afford the corresponding diazo **S-2** in 60-90% yield.

Step 3: Following the reported procedure,[1] to a 100-mL oven-dried round bottom flask containing a magnetic stirring bar, **S-2** (2.0 mmol) and Et_3N (3.0 mmol, 1.5 eq) in DCM (10 mL), was added *Si*OTf [TIPSOTf or TBSOTf (2.2 mmol, 1.1 eq)] slowly at 0 °C. After the reaction mixture was stirred for 0.5-1 h under these conditions, hexanes (30 mL) were added, followed by saturated aqueous $NaHCO_3$ (40 mL). The organic phase was separated and washed two more times with saturated aqueous $NaHCO_3$ (40 mL X 2) and dried with anhydrous Na_2SO_4. After evaporating the solvents the residue was purified by column chromatography on silica gel which was pre-treated with 5 vol.% triethylamine/hexanes (eluent: pure hexanes) to give the desired products **6** in 90-95% yield.

2. Supplementary Discussion

2.1 Optimization of the reaction conditions

Supplementary Table 1. Optimization of reaction conditions for the addition of *tert*-butyl nitrite to enoldiazo compound **6**.[a]

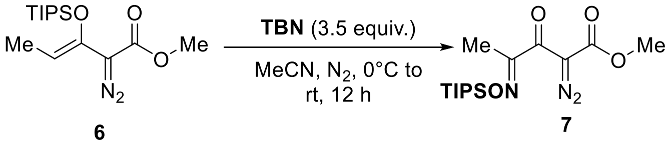

entry	variation from standard condition	7(%)
**1**	**none**	**93(83)**
2	in the dark	90
3	45 °C instead of 0 °C to rt	46
4	isopentyl nitrite instead of TBN	nd
5	THF, rt	nd
6	DCM, rt	18
7	$CHCl_3$, rt	23
8	acetone, rt	nd
9	cyclohexane, rt	nd
10	toluene, rt	nd
11	hexane, rt	nd

[a]Reaction conditions: ^{t}BuONO (3.5 equiv.) was added dropwise to a 2.0 mL solution containing **6** (0.20 mmol, 0.10 M) at 0 °C under N_2. The reaction was continued for 12 h at room temperature. Another 1.0 equiv. of TBN was added after 24 h. [b]The percentage of **7** compared with the initial amount of **6** was determined by proton NMR spectral analysis using $CHBr_3$ as internal standard. nd = not detected. Parenthesis isolated yield. Concentration 0.1M.

Supplementary Table 2. Optimization of reaction conditions for the synthesis of 1-ester-2,3-diketo-4-oxime **8** and its hydrate, **14.**[a]

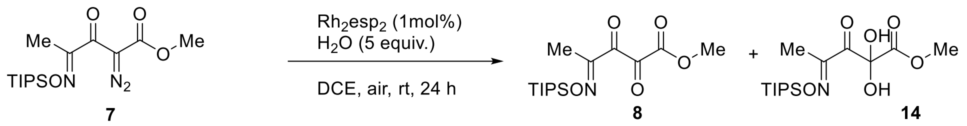

entry	variation from standard condition	8(%)[b] + 14(%)[b] (8:14)[c]
**1**	**none**	94 (1:1.2)
	no H_2O	complex
2	under N_2	nd
3	DMDO instead of $Rh_2(esp)_2$	58 (1:3)[d]
4	DCM	72 (1:1.8)
5	DCM, H_2O (10 equiv.)	60 (1.8:1)
6	$CHCl_3$	80 (1:1.8)
7	THF	nd[e]
8	MeCN	nd[f]
9	$Rh_2(Oct)_4$ (1 mol%)	complex[g]
10	$Rh_2(hfb)_4$ (1 mol%)	nd[h]
11	$Rh_2(OAc)_4$ (1 mol%)	6[j]
12	JohnPhosAu(MeCN)SbF_6 (1 mol%)	nd[k]
13	$AgSbF_6$ (5 mol%)	complex[l]
14	$[Cu(CH_3CN)_4]PF_6$ (5 mol%)	complex[m]

[a]Reaction conditions: using a syringe pump, diazo compound **7** (0.1 mmol) in 1 mL of DCE was added to a DCE solution (1 mL) containing H_2O (5.0 equiv.) and $Rh_2(esp)_2$ (1.0×10^{-3} mmol) over 1 h. The reaction was continued for the indicated time at room temperature. [b]Isolated yield. [c]The **8**:**14** ratio was determined by ^{1}H-NMR analysis. [d]40% of **7** was unreacted. [e]92% of **7** was unreacted. [f]96% of **7** was unreacted. [g]82% conversion. [h]99% of **7** was unreacted. [j]85% of **7** was unreacted. [k]98% of **7** was unreacted. [l]100% conversion. [m]100% conversion. nd = not detected. $Rh_2(hfb)_4$ = rhodium(II) heptafluorobutyrate dimer.

2.2 Kinetic Studies

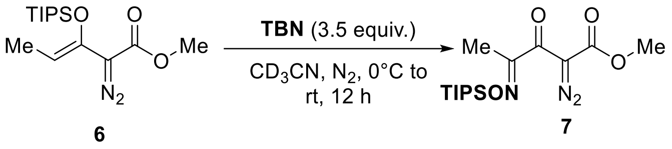

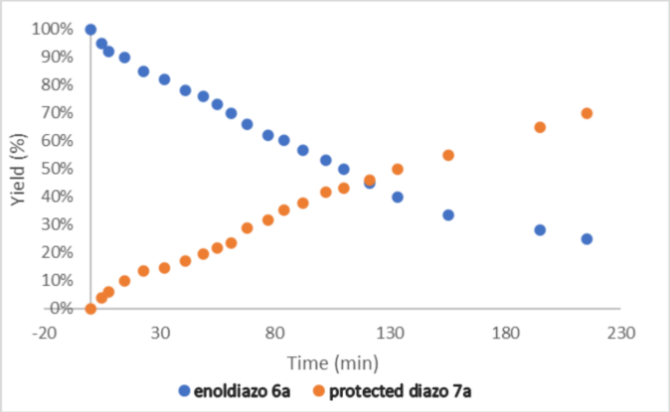

Supplementary Fig. 3. Time course of reaction between ^{t}BuONO and enoldiazo **6a**. ^{t}BuONO (3.5 equiv.) was added dropwise to a 0.6 mL CD_3CN solution containing **6a** (0.1 mmol) at 0 °C under N_2. The reaction was continued and monitored during the time for 4 h at room temperature.

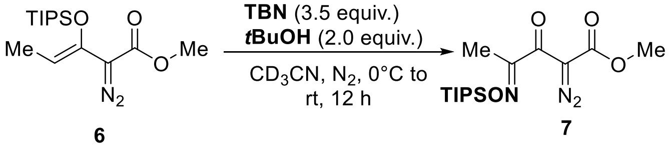

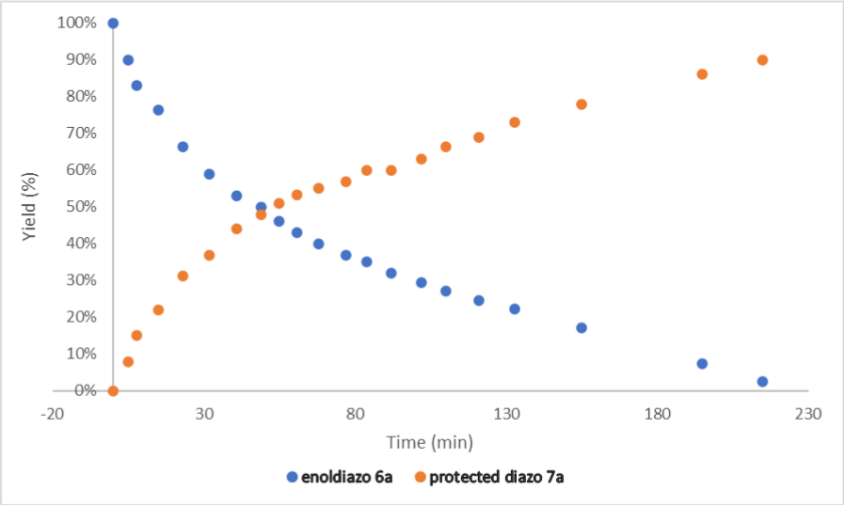

Supplementary Fig. 4. Time course reaction between ^{t}BuONO, *t*BuOH and enoldiazo **6a**. ^{t}BuONO (3.5 equiv.) was added dropwise to a 0.6 mL CD_3CN solution containing **6a** (0.2 mmol) and *t*BuOH (2.0 equiv.) at 0 °C under N_2. The reaction was continued and monitored during the time for 4 h at room temperature.

2.3 Oxidation potential studies

Supplementary Table 3. Oxidation potential of enediol compound **12a**[a]

Entry	Solvent	Potential (vs fc/fc$^+$)
1	ACN	0.76 V
2	DMF	0.65 V
3	DCM	0.82 V

[a]Differential Pulse Voltammetry (DPV) was performed in acetonitrile (ACN), dichloromethane (DCM), and dimethylformamide (DMF) at 23°C on a CH Instruments 620D electrochemical workstation. A three-electrode setup was employed comprising a 2 mm diameter glassy carbon working electrode, a platinum wire auxiliary electrode, and a Ag/AgCl quasi-reference electrode. Triply recrystallized Bu_4NPF_6 was used as the supporting electrolyte. All electrochemical data were referenced to the ferrocene/ferrocenium couple at 0.00 V.

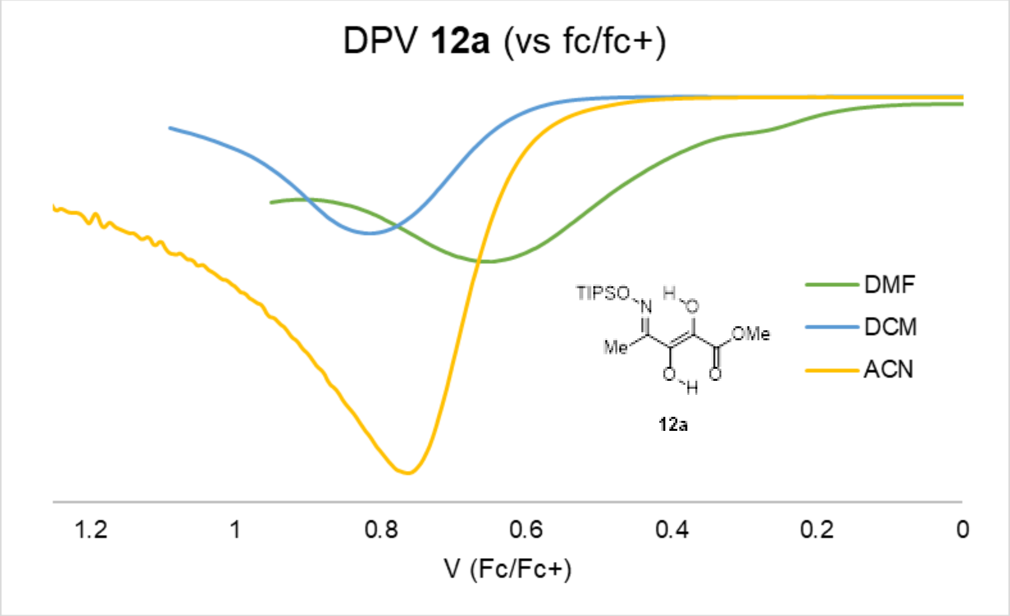

Supplementary Fig. 5. Differential pulse voltammetry (DPV) measurements of enediol **12a** with different solvent. Green: *N,N*-dimethylformamide. Blue: methylene chloride. Yellow: acetonitrile.

Supplementary Table 4. Oxidation potential of Ascorbic Acid[a]

Entry	Solvent	Potential (vs fc/fc$^+$)
1	ACN : H_2O (9:1)	0.47 V
2	DMF	0.20 V

[a]Differential Pulse Voltammetry (DPV) and Cyclic voltammetry (CV) were performed in acetonitrile/water and dimethylformamide (DMF) at 23°C on a CH Instruments 620D electrochemical workstation. In entry 1 a 9:1 ratio of ACN to deionized (DI) water was used to improve solubility of the compound. A three-electrode setup was employed comprising a 2 mm diameter glassy carbon working electrode, a platinum wire auxiliary electrode, and a Ag/AgCl

quasi-reference electrode. Triply recrystallized Bu_4NPF_6 was used as the supporting electrolyte. All electrochemical data were referenced to the ferrocene/ferrocenium couple at 0.00 V.

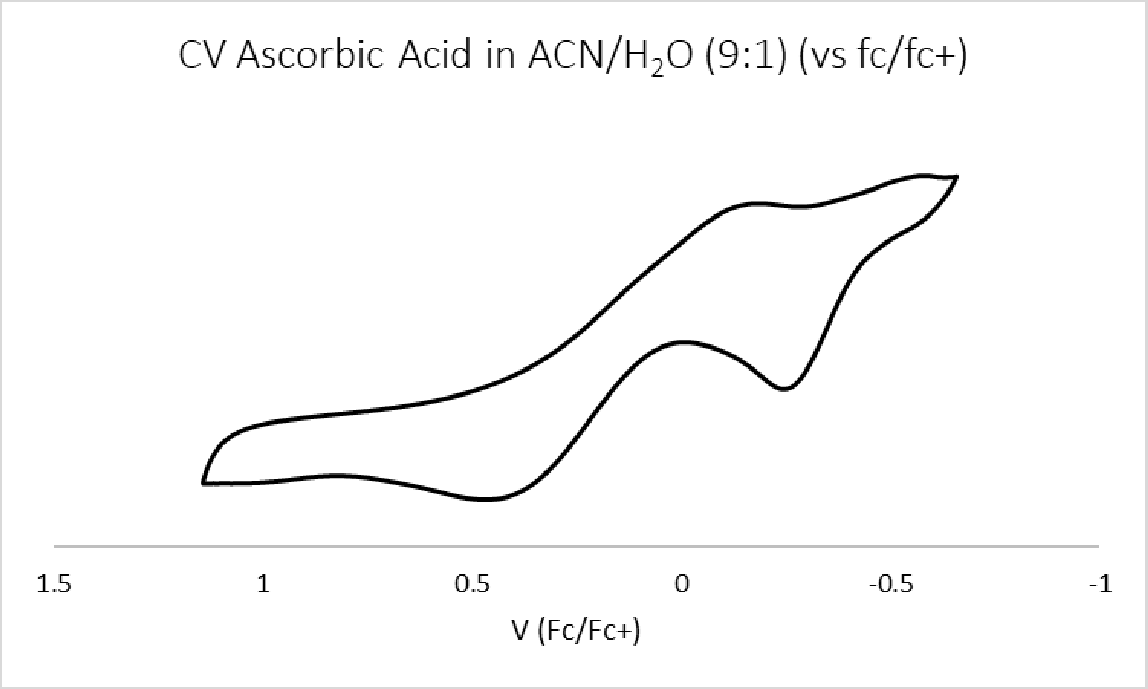

Supplementary Fig. 6. Cyclic voltammetry measurement of ascorbic acid in ACN/H_2O (9:1).

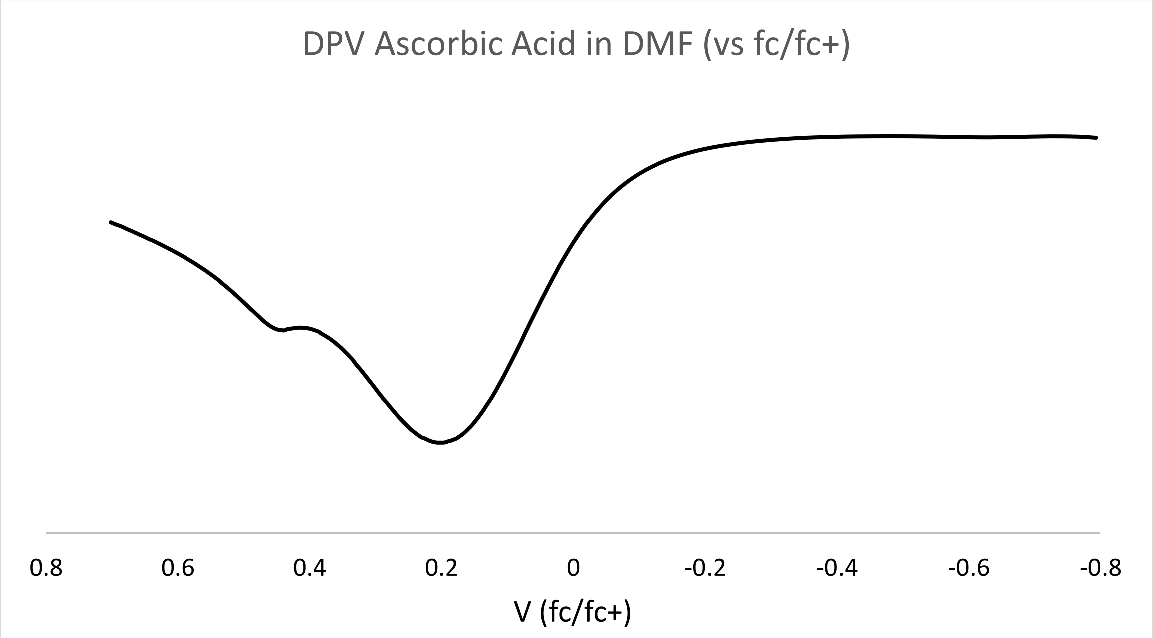

Supplementary Fig. 7. Differential pulse voltammetry (DPV) measurement of ascorbic acid in DMF.

2.4 Solvent influence on the equilibrium between α-hydroxy carbonyl (13a) and enediol (12a) compounds

Supplementary Table 5. NMR studies of the equilibrium between α-hydroxy carbonyl (**13a**) and enediol (**12a**) compounds[a]

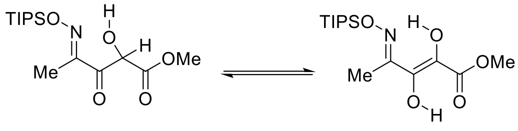

Solvent	**13a** (%)	**12a** (%)
CD_3CN (10 h)	77	23
$CDCl_3$ (10 h)	0	100
DMF-d_7 (10 h)	10	90

[a]The **13a**:**12a** ratio was determined by ^{1}H-NMR (500 MHz) analysis from the integral values of the methyl group of **13a** (s, 2.02 ppm) and the methyl group of **12a** (s, 2.18 ppm) at room temperature.

2.5 General procedures

Formation of 1-Ester-2-diazo-3-keto-4-protected Oximes. *tert*-Butyl nitrite (3.5 equiv., 1.4 mmol) was added dropwise over 1 min in a round bottom flask containing a solution of enoldiazo compound **6** (0.4mmol, 0.10 M in MeCN) at 0°C under a N_2 atmosphere. The reaction solution was slowly warmed to room temperature, and the progress of the reaction was followed by TLC until consumption of the vinyldiazo compounds was complete. The color of solution went from orange/yellow to colorless. The solvent was then removed under reduced pressure, and the residue was purified by flash chromatography (hexane/ethyl acetate = 5/1) to give the desired diazo product **7**.

Formation of 1-Ester-2-diazo-3-keto-4-oximes. To a solution of the protected oxime **7** (0.1 mmol, 0.20 M in THF) in a dry 8-mL vial was added TBAF (1.5 equiv., 0.15 mmol) at 0 °C all at once. The progress of the reaction was followed by TLC until consumption of the protected oxime was complete, and the residue was purified by flash chromatography (dichloromethane/methanol = 9/1) to give the desired product **10**.

Formation of Dioxolene Carboxylate 11. To solution of $Rh_2(OAc)_4$ (5 mol%) in 2 mL of acetone in a dry 8-mL tube, diazo-oxime **10** (0.1 mmol,) was added at room temperature. The temperature was increased to 45 °C and the solution was stirred for 24 h. The residue was then purified by flash chromatography (hexane/ethyl acetate = 5/1) to give the desired dioxolene product **11** (16.1 mg, 50%).

Formation of Enediol and α-Hydroxycarbonyl Compounds. In a dry 8-mL tube, to solution of $Rh_2(esp)_2$ (1 mol%) in 1 mL of dichloroethane with H_2O (5.0 equiv., 0.5 mmol), diazo protected oxime **7** (0.1 mmol, 0.10 M in DCE) was added over 1 h with a syringe pump at room temperature under N_2. The color of the solution changed from light blue/green to light brown after 24 h. The vial was moved into a glove box, and the solvent was then removed. Deuterated solvent was added

under N_2 to determine the equilibrium between α-hydroxy carbonyl (**13a**) and enediol (**12a**) compounds. The enediol **12** is stable under N_2, but in the presence of air **12** is rapidly converted to 2,3-diketo oxime **8** and its hydrate form **14**.

Formation of 1-Ester-2,3-diketo-4-oximes. In a dry 8-mL tube, to solution of $Rh_2(esp)_2$ (1 mol%) in 1 mL of dichloroethane with H_2O (5.0 equiv., 0.5 mmol), diazo-protected oxime **7** (0.1 mmol, 0.10 M in DCE) was added over 1 h with a syringe pump at room temperature. The color of the solution changed from light blue/green to light brown after 24 h. The residue was then purified by flash chromatography (hexane/ethyl acetate = 3/1) to give the desired product as an inseparable mixture of 1-ester-2,3-diketo-4-oximes **8** and their hydrate **14**.

2.6 Analytical and spectral characterization data for products

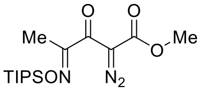

7a **Methyl (*Z*)-2-Diazo-3-oxo-4-[(triisopropylsilyl)oxyimino]pentanoate, 7a**. Colorless oil (113.2 mg, 83% yield), 0.4 mmol scale reaction. Flash column chromatography conditions: hexane:ethyl acetate = 5:1.**^{1}H NMR** (500 MHz, chloroform-*d*) δ 3.86 (s, 3H), 2.07 (s, 3H), 1.24 (hept, *J* = 7.5 Hz, 3H), 1.07 (d, *J* = 7.5 Hz, 18H).**^{13}C NMR** (126 MHz, $CDCl_3$) δ 179.0, 162.5, 160.2, 52.8, 17.7, 11.9, 10.0. **^{15}N NMR** (50.664 MHz, $CDCl_3$) δ 390.4. **HRMS** (ESI) calculated for $[M+Na]^+$ $C_{15}H_{27}N_3O_4Si$ m/z 364.1663, observed: 364.1658

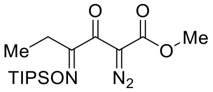

7b **Methyl (*Z*)-2-Diazo-3-oxo-4-[(triisopropylsilyl)oxyimino]hexanoate**, **7b**. Colorless oil (115.1 mg, 81% yield), 0.4 mmol scale reaction. Flash column chromatography conditions: hexane:ethyl acetate = 5:1. **^{1}H NMR** (500 MHz, chloroform-*d*) δ 3.86 (s, 3H), 2.62 (q, *J* = 7.6 Hz, 2H), 1.23 (hept, *J* = 7.4 Hz, 3H), 1.08 (t, *J* = 7.6 Hz, 3H), 1.07 (d, *J* = 7.4 Hz, 18H). **^{13}C NMR** (126 MHz, $CDCl_3$) δ 179.2, 164.4, 162.6, 52.9, 18.1, 17.8, 12.1, 10.5. **HRMS** (ESI) calculated for $[M+Na]^+$: $C_{16}H_{29}N_3O_4Si$ m/z: 378.1820, observed: 378.1808.

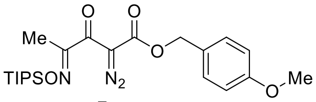

7c **4-Methoxybenzyl (*Z*)-2-Diazo-3-oxo-4-[(triisopropylsilyl)oxyimino] pentanoate**, **7c**. Colorless oil (166.4 mg, 93% yield), 0.4 mmol scale reaction. Flash column chromatography conditions: hexane:ethyl acetate = 5:1. **^{1}H NMR** (500 MHz, chloroform-*d*) δ 7.34 (d, *J* = 8.7 Hz, 2H), 6.89 (d, *J* = 8.7 Hz, 2H), 5.23 (s, 2H), 3.80 (s, 3H), 2.06 (s, 3H), 1.23 (hept, *J* = 7.4 Hz, 3H), 1.06 (d, *J* = 7.4 Hz, 18H). **^{13}C NMR** (126 MHz, $CDCl_3$) δ 178.9, 161.7, 160.2, 159.8, 130.2, 127.5, 114.0, 67.1, 55.3, 17.7, 11.9, 10.0. **HRMS** (ESI) calculated for $[M+H]^+$: $C_{22}H_{33}N_3O_5Si$ m/z: 448.2262, observed: 448.2250.

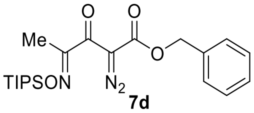

7d **Benzyl (*Z*)-2-Diazo-3-oxo-4-[(triisopropylsilyl)oxyimino]pentanoate**, **7d**. Colorless oil (133.5 mg, 80% yield), 0.4 mmol scale reaction. Flash column chromatography conditions: hexane:ethyl acetate = 5:1. **^{1}H NMR** (500 MHz, chloroform-*d*) δ 7.43 – 7.29 (m, 5H), 5.30 (s, 2H), 2.07 (s, 3H), 1.24 (hept, *J* = 7.4 Hz, 3H), 1.07 (d, *J* = 7.4 Hz, 18H).**^{13}C NMR** (126

MHz, $CDCl_3$) δ 178.9, 161.7, 160.2, 128.6, 128.4, 128.2, 67.2, 17.7, 12.3, 11.9, 10.0. **HRMS** (ESI) calculated for $[M+H]^+$: $C_{21}H_{31}N_3O_4Si$ m/z: 418.2157, observed: 418.2148.

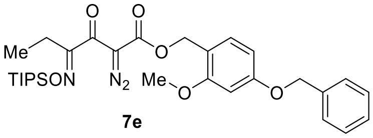

7e

4-(Benzyloxy)-2-methoxybenzyl (*Z*)-2-Diazo-3-oxo-4-[(triisopropylsilyl)oxyimino]hexanoate, **7e**. Colorless oil (167.3 mg, 72% yield), 0.4 mmol scale reaction. Flash column chromatography conditions: hexane:ethyl acetate = 5:1. **^{1}H NMR** (500 MHz, chloroform-*d*) δ 7.45 (d, *J* = 7.2 Hz, 2H), 7.39 (t, *J* = 7.2 Hz, 2H), 7.32 (t, *J* = 7.2 Hz, 2H), 6.99 (d, *J* = 2.0 Hz, 1H), 6.95 – 6.85 (comp, 2H), 5.24 (s, 2H), 5.18 (s, 2H), 3.93 (s, 3H), 2.64 (q, *J* = 7.6 Hz, 2H), 1.25 (hept, *J* = 7.4 Hz, 3H), 1.10 – 1.08 (comp, 21H).**^{13}C NMR** (126 MHz, $CDCl_3$) δ 164.4, 161.8, 149.6, 148.3, 137.0, 128.5, 128.4, 127.8, 127.2, 121.3, 113.7, 112.4, 71.0, 67.3, 56.0, 17.9, 17.7, 12.3, 12.0, 10.4. **HRMS** (ESI) calculated for $[M+H]^+$: $C_{30}H_{41}N_3O_6Si$ m/z: 568.2837, observed: 568.2835.

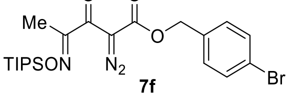

7f

4-Bromobenzyl (*Z*)-2-Diazo-3-oxo-4-[(triisopropylsilyl)oxyimino]-pentanoate, **7f**. Colorless oil (148.5 mg, 75% yield), 0.4 mmol scale reaction. Flash column chromatography conditions: hexane:ethyl acetate = 5:1. **^{1}H NMR** (500 MHz, chloroform-*d*) δ 7.50 (d, *J* = 8.2 Hz, 2H), 7.28 (d, *J* = 8.2 Hz, 2H), 5.24 (s, 2H), 2.07 (s, 3H), 1.23 (hept, *J* = 7.5 Hz, 3H), 1.07 (d, *J* = 7.5 Hz, 18H).**^{13}C NMR** (126 MHz, $CDCl_3$) δ 178.7, 161.8, 160.3, 134.3, 131.8, 129.9, 122.6, 66.5, 17.7, 11.9, 10.0. **HRMS** (ESI) calculated for $[M+H]^+$: $C_{21}H_{30}BrN_3O_4Si$ m/z: 496.1262, observed: 496.1260.

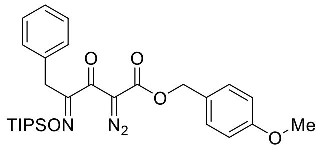

7g

4-Methoxybenzyl (*Z*)-2-Diazo-3-oxo-5-phenyl-4-[(triisopropylsilyl)-oxyimino]pentanoate, **7g**. Colorless oil (167.5 mg, 80% yield), 0.4 mmol scale reaction. Flash column chromatography conditions: hexane:ethyl acetate = 5:1. **^{1}H NMR** (500 MHz, chloroform-*d*) δ 7.36 (d, *J* = 8.3 Hz, 2H), 7.30 (d, *J* = 7.5 Hz, 2H), 7.26 (t, *J* = 7.5 Hz, 2H), 7.19 (t, *J* = 7.5 Hz, 1H), 6.92 (d, *J* = 8.3 Hz, 2H), 5.25 (s, 2H), 4.05 (s, 2H), 3.83 (s, 4H), 1.27 (hept, J = 7.5 Hz, 3H), 1.07 (d, J = 7.5 Hz, 18H).**^{13}C NMR** (126 MHz, $CDCl_3$) δ 178.5, 161.4, 161.3, 159.8, 136.0, 130.3, 129.37, 128.5, 127.5, 126.5, 114.0, 67.1, 55.3, 29.9, 17.7, 12.4, 11.9. **HRMS** (ESI) calculated for $[M+H]^+$: $C_{28}H_{37}N_3O_5Si$ m/z: 524.2575, observed: 524.2571.

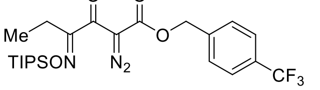

7h

4-(Trifluoromethyl)benzyl (*Z*)-2-Diazo-3-oxo-4-[(triisopropylsilyl)-oxyimino]hexanoate, **7h**. Colorless oil (143.7 mg, 72% yield), 0.4 mmol scale reaction. Flash column chromatography conditions: hexane:ethyl acetate = 5:1. **^{1}H NMR** (500 MHz, chloroform-*d*) δ 7.66 (d, *J* = 8.1 Hz, 2H), 7.55 (d, *J* = 8.1 Hz, 2H), 5.37 (s, 2H), 2.66 (q, *J* = 7.5 Hz, 2H), 1.26 (hept, *J* = 7.4 Hz, 4H), 1.10 (d, *J* = 7.4 Hz, 18H), 1.10 (t, *J* = 7.5 Hz, 3H).**^{13}C NMR** (126 MHz, $CDCl_3$) δ 178.7, 164.4, 161.9, 139.3, 130.6 (q, *J* = 32.4 Hz), 128.2, 125.6 (q, *J* = 3.7 Hz), 123.9 (d, *J* = 272.4 Hz), 66.3, 17.7, 12.3, 11.9, 10.4. **HRMS** (ESI) calculated for $[M+H]^+$: $C_{23}H_{32}F_3N_3O_4Si$ m/z: 500.2187, observed: 5002182.

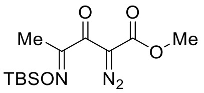

7i **Methyl (*Z*)-4-[(*tert*-Butyldimethylsilyl)oxyimino]-2-diazo-3-oxopentanoate, 7i**. Colorless oil (89.7 mg, 75% yield), 0.4 mmol scale reaction. Flash column chromatography conditions: hexane:ethyl acetate = 5:1. **^{1}H NMR** (500 MHz, chloroform-*d*) δ 3.89 (s, 3H), 2.08 (s, 3H), 0.97 (s, 9H), 0.23 (s, 6H).**^{13}C NMR** (126 MHz, $CDCl_3$) δ 178.8, 162.4, 159.9, 52.7, 25.7, 18.0, 9.9, -5.1. **HRMS** (ESI) calculated for $[M+H]^+$: $C_{12}H_{21}N_3O_4Si$ m/z: 300.1374, observed: 300.1372.

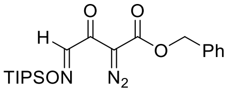

7j **Benzyl (*Z*)-2-Diazo-3-oxo-4-[(triisopropylsilyl)oxyimino]butanoate, 7j**. Colorless oil (19.3 mg, 12% yield), 0.4 mmol scale reaction. Flash column chromatography conditions: hexane:ethyl acetate = 5:1. **^{1}H NMR** (500 MHz, chloroform-*d*) δ 8.25 (s, 1H), 7.45 – 7.32 (comp, 5H), 5.30 (s, 2H), 1.26 (hept, 7.2 Hz, 3H), 1.08 (d, *J* = 7.2 Hz, 18H).**^{13}C NMR** (126 MHz, $CDCl_3$) δ 176.8, 160.8, 150.8, 135.0, 128.7, 128.6, 128.5, 128.4, 67.3, 17.6, 11.8. **HRMS** (ESI) calculated for $[M+H]^+$: $C_{20}H_{29}N_3O_4Si$ m/z: 404.2000, observed: 404.1997.

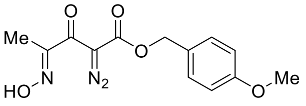

10c **4-Methoxybenzyl (*Z*)-2-Diazo-4-(hydroxyimino)-3-oxopentanoate, 10c**. white solid (108.3 mg, 93% yield), 0.4 mmol scale reaction. Recrystallization. **^{1}H NMR** (500 MHz, DMSO-*d*$_6$) δ 12.18 (s, 1H), 7.34 (d, *J* = 8.7 Hz, 2H), 6.95 (d, *J* = 8.7 Hz, 2H), 5.17 (s, 2H), 3.76 (s, 3H), 1.87 (s, 3H). **^{13}C NMR** (126 MHz, DMSO) δ 178.9, 161.2, 159.3, 154.0, 129.9, 127.6, 113.8, 66.2, 55.1, 9.3. **HRMS** (ESI) calculated for $[M+Na]^+$: $C_{13}H_{13}N_3O_5$ m/z: 314.0747, observed: 314.0748.

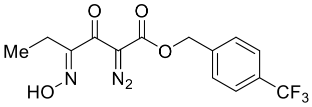

10h **4-(Trifluoromethyl)benzyl (*Z*)-2-Diazo-4-(hydroxyimino)-3-oxo-hexanoate, 10h**. white solid (131.7 mg, 96% yield), 0.4 mmol scale reaction. Recrystallization. **^{1}H NMR** (500 MHz, DMSO-*d*$_6$) δ 12.17 (s, 1H), 7.76 (d, *J* = 8.0 Hz, 2H), 7.63 (d, *J* = 8.0 Hz, 2H), 5.35 (s, 2H), 2.43 (q, *J* = 7.6 Hz, 2H), 0.98 (t, *J* = 7.6 Hz, 3H). **^{13}C NMR** (126 MHz, DMSO) δ 179.27, 161.67, 158.59, 158.51, 141.05, 129.0 (q, *J* = 32.3 Hz), 125.8 (q, *J* = 3.5 Hz), 124.6 (q, *J* = 271.1 Hz), 72.03, 65.93, 17.38, 10.59. **HRMS** (ESI) calculated for $[M+H]^+$: $C_{14}H_{12}F_3N_3O_4$ m/z: 344.9853, observed: 344.9854.

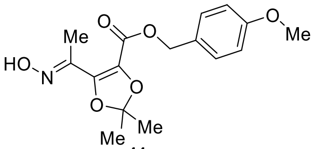

11 **4-Methoxybenzyl (*Z*)-5-(1-(Hydroxyimino)ethyl)-2,2-dimethyl-1,3-dioxole-4-carboxylate, 11**. white solid (16.1 mg, 50% yield), 0.1 mmol scale reaction. Flash column chromatography conditions: hexane:ethyl acetate = 5:1. **^{1}H NMR** (500 MHz, chloroform-*d*) δ 8.66 (s, 1H), 7.36 (d, *J* = 8.2 Hz, 1H), 6.91 (d, *J* = 8.2 Hz, 1H), 5.23 (s, 2H), 3.83 (s, 3H), 2.15 (s, 2H), 1.66 (s, 6H).**^{13}C NMR** (126 MHz, $CDCl_3$) δ 159.9, 159.7, 147.4, 142.6, 130.5, 129.2, 127.4, 115.6, 113.9, 66.7, 55.2, 25.3, 12.8. **HRMS** (ESI) calculated for $[M+Na]^+$: $C_{16}H_{19}NO_6$ m/z: 344.1102, observed: 344.1105.

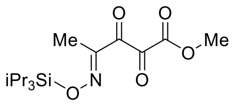

8a **Methyl (*Z*)-2,3-Dioxo-4-[(triisopropylsilyl)oxyimino]pentanoate**, **8a**. Colorless oil (**8a/14a** = 1:1,5; 30.9 mg, 94% yield), 0.1 mmol scale reaction. Flash column chromatography conditions: hexane:ethyl acetate = 3:1. **^{1}H NMR** (500 MHz, chloroform-*d*) δ 3.88 (s, 3H), 2.07 (s, 3H), 1.23 – 1.17 (comp, 3H), 1.03 (*d*, J = 7.5 Hz, 18H).**^{13}C NMR** (126 MHz, $CDCl_3$) δ 191.4, 186.5, 166.2, 157.4, 53.3, 17.5, 11.7, 8.1. **^{15}N NMR** (50.664 MHz, $CDCl_3$) δ 419.6. **HRMS** (ESI) calculated for $[M+H]^+$: $C_{15}H_{27}NO_5Si$ m/z: 330.1731, observed: 330.1730.

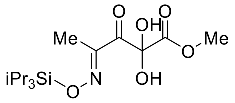

14a **Methyl (*Z*)-2,2-Dihydroxy-3-oxo-4-[(triisopropylsilyl)oxyimino]pentan-oate**, **14a**. Colorless oil. **^{1}H NMR** (500 MHz, chloroform-*d*) δ 5.08 (s, 2H), 3.79 (s, 3H), 2.09 (s, 3H), 1.31 – 1.23 (comp, 3H), 1.08 (d, *J* = 7.5 Hz, 18H).**^{13}C NMR** (126 MHz, $CDCl_3$) δ 190.7, 169.3, 157.4, 91.1, 53.4, 17.6, 11.7, 9.6. **^{15}N NMR** (50.664 MHz, $CDCl_3$) δ 411.6. **HRMS** (ESI) calculated for $[M+H]^+$: $C_{15}H_{29}NO_6Si$ m/z: 348.1837, observed: 348.1836.

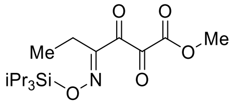

8b **Methyl (*Z*)-2,3-Dioxo-4-[(triisopropylsilyl)oxyimino]hexanoate**, **8b**. Colorless oil (**8b/14b** = 1:1,6; 30.9 mg, 90% yield), 0.1 mmol scale reaction. Flash column chromatography conditions: hexane:ethyl acetate = 3:1. **^{1}H NMR** (500 MHz, chloroform-*d*) δ 3.88 (s, 3H), 2.64 – 2.56 (comp, 2H), 1.24 – 1.17 (comp, 3H), 1.11 (*t*, J = 7.6 Hz, 3H), 1.08 – 1.01 (comp, 18H).**^{13}C NMR** (126 MHz, $CDCl_3$) δ 191.5, 186.9, 169.4, 158.6, 53.4, 17.8, 17.6, 11.9, 10.1. **HRMS** (ESI) calculated for $[M+H]^+$: $C_{16}H_{29}NO_5Si$ m/z: 344.1888, observed: 344.1887.

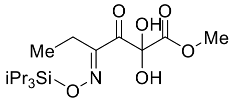

14b **Methyl (*Z*)-2,2-Dihydroxy-3-oxo-4-[(triisopropylsilyl)oxyimino]-hexanoate**, **14b**. Colorless oil. **^{1}H NMR** (500 MHz, chloroform-*d*) δ 5.07 (s, 2H), 3.79 (s, 3H), 2.68 – 2.61 (comp, 2H), 1.32 – 1.22 (comp, 3H), 1.08 (d, *J* = 7.5 Hz, 18H), 1.04 – 1.02 (comp, 3H). **^{13}C NMR** (126 MHz, $CDCl_3$) δ 190.6, 165.0, 162.0, 91.3, 53.5, 17.6, 16.3, 11.8, 9.9. **HRMS** (ESI) calculated for $[M+H]^+$: $C_{16}H_{31}NO_6Si$ m/z: 362.1993, observed: 362.1993.

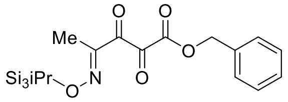

8d **Benzyl (*Z*)-2,3-Dioxo-4-[(triisopropylsilyl)oxyimino]pentanoate**, **8d**. Colorless oil (**8d/14d** = 1:2.7; 28.4 mg, 70% yield), 0.1 mmol scale reaction. Flash column chromatography conditions: hexane:ethyl acetate = 3:1. **^{1}H NMR** (500 MHz, chloroform-*d*) δ 7.34 – 7.31 (comp, 2H), 7.29 – 7.23 (comp, 3H), 5.20 (s, 1H), 2.08 (s, 1H), 1.28 (hept, *J* = 7.4 Hz, 3H), 1.08 (d, *J* = 7.4 Hz, 18H). **^{13}C NMR** (126 MHz, $CDCl_3$) δ 191.7, 186.6, 160.7, 157.7, 134.0, 128.8, 128.7, 127.9, 68.3, 17.6, 11.9, 9.8. **HRMS** (ESI) calculated for $[M+H]^+$: $C_{21}H_{31}NO_5Si$ m/z: 406.2044, observed: 406.2044.

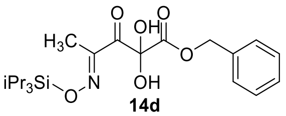
14d

Benzyl (*Z*)-2,2-Dihydroxy-3-oxo-4-[(triisopropylsilyl)oxyimino]-pentanoate, **14d**. Colorless oil. **^{1}H NMR** (500 MHz, chloroform-*d*) δ 7.39 – 7.31 (comp, 5H), 5.28 (s, 2H), 5.10 (s, 1H), 2.06 (s, 3H), 1.17 (hept, *J* = 7.5, 3H), 1.00 (d, *J* = 7.5 Hz, 18H). **^{13}C NMR** (126 MHz, $CDCl_3$) δ 190.7, 168.9, 158.1, 134.6, 129.1, 128.7, 91.5, 68.6, 17.8, 11.8, 8.1. **HRMS** (ESI) calculated for $[M+H]^+$: $C_{21}H_{33}NO_6Si$ m/z: 424.2150, observed: 424.2150.

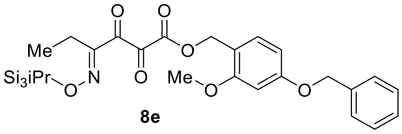
8e

4-(Benzyloxy)-2-methoxybenzyl (*E*)-2,3-Dioxo-4-[(triisopropylsilyl)oxyimino]hexanoate, **8e**. Colorless oil (**8e/14e** = 1:1.7; 40.0 mg, 72% yield), 0.1 mmol scale reaction. Flash column chromatography conditions: hexane:ethyl acetate = 3:1. **^{1}H NMR** (500 MHz, chloroform-*d*) 7.40 – 7.32 (comp, 2H), 7.34 – 7.26 (comp, 3H), 6.90 – 6.89 (m, 1H), 6.83 (s, 1H), 6.82 – 6.80 (m, 1H), 5.19 (s, 2H), 5.16 (s, 2H), 3.88 (s, 3H), 2.58 (q, *J* = 7.6 Hz, 2H), 1.17 (hept, *J* = 7.4, 3H), 1.04 – 1.02 (comp, 3H), 1.00 (d, *J* = 7.4 Hz, 18H).**^{13}C NMR** (126 MHz, $CDCl_3$) δ191.7, 186.9, 164.9, 158.0, 149.9, 148.9, 137.1, 128.7, 128.1, 127.6, 127.4, 121.9, 113.8, 112.7, 71.1, 68.8, 56.1, 17.6, 17.5, 11.8, 9.9. **HRMS** (ESI) calculated for $[M+Na]^+$: $C_{30}H_{41}NO_7Si$ m/z: 578.2545, observed: 578.2542.

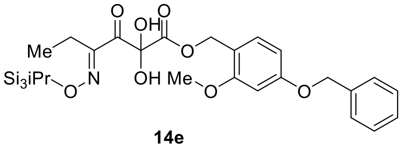
14e

4-(Benzyloxy)-2-methoxybenzyl (*E*)-2,2-Dihydroxy-3-oxo-4-[(triisopropylsilyl)oxyimino]hexanoate, **14e**. Colorless oil. **^{1}H NMR** (500 MHz, chloroform-*d*) δ 7.45 – 7.39 (comp, 3H), 7.34 – 7.26 (comp, 2H), 6.85 (s, 1H), 6.83 – 6.79 (m, 1H), 6.76 (d, *J* = 8.1, Hz, 1H), 5.14 (s, 2H), 5.12 (s, 2H), 5.09 (s, 2H), 3.86 (s, 3H), 2.63 (q, *J* = 7.6 Hz, 2H), 1.29 (hept, *J* = 7.5 Hz, 3H), 1.09 (d, *J* = 7.5 Hz, 18H), 1.04 – 1.01 (comp, 3H).**^{13}C NMR** (126 MHz, $CDCl_3$) δ 190.5, 168.9, 162.1, 149.8, 136.9, 128.7, 128.0, 127.4, 126.9, 120.8, 111.9, 91.5, 71.1, 68.4, 56.1, 17.8, 16.2, 11.9, 10.1. **HRMS** (ESI) calculated for $[M+Na]^+$: $C_{30}H_{43}NO_8Si$ m/z: 596.2650, observed: 596.2651.

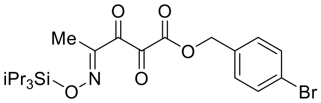
8f

4-Bromobenzyl (*Z*)-2,3-Dioxo-4-[(triisopropylsilyl)oxyimino]pentanoate, **8f**. Colorless oil (**8f/14f** = 1:2.7; 30.4 mg, 63% yield), 0.1 mmol scale reaction. Flash column chromatography conditions: hexane:ethyl acetate = 3:1. **^{1}H NMR** (500 MHz, chloroform-*d*) δ 7.51 (d, *J* = 8.4 Hz, 1H), 7.24 (d, *J* = 8.4 Hz, 1H), 5.09 (s, 2H), 2.06 (s, 3H), 1.15 (hept, *J* = 7.4 Hz, 3H), 1.00 (d, *J* = 7.4 Hz, 14H).**^{13}C NMR** (126 MHz, $CDCl_3$) δ 191.6, 186.3, 160.7, 157.8, 132.9, 132.1, 130.4, 123.3, 67.7, 17.6, 11.8, 8.3. **HRMS** (ESI) calculated for $[M+H]^+$: $C_{21}H_{30}BrNO_5Si$ m/z: 484.1149, observed: 484.1149.

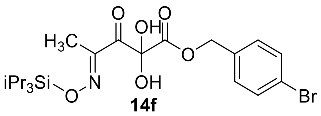
14f

4-Bromobenzyl (*Z*)-2,2-Dihydroxy-3-oxo-4-[(triisopropylsilyl)-oxyimino]pentanoate, **14f**. Colorless oil. **^{1}H NMR** (500 MHz, chloroform-*d*) δ 7.47 (d, *J* = 8.4 Hz, 2H), 7.14 (d, *J* = 8.4 Hz, 2H), 5.22 (s, 2H), 5.14 (s, 2H), 2.09 (s, 3H), 1.27 (hept, *J* = 7.4 Hz, 3H), 1.08 (d, *J* = 7.4 Hz, 18H).**^{13}C NMR** (126 MHz, $CDCl_3$) δ 190.6, 168.7, 157.9, 133.6, 131.9,

129.5, 122.8, 91.5, 67.4, 17.8, 11.9, 9.8. **HRMS** (ESI) calculated for $[M+H]^+$: $C_{21}H_{32}BrNO_6Si$ m/z: 502.1255, observed: 502.1255.

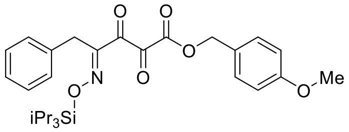

8g **4-Methoxybenzyl (*Z*)-2,3-Dioxo-5-phenyl-4-[(triisopropylsilyl)-oxyimino]pentanoate**, **8g**. Colorless oil (**8g/14g** = 1:1.1; 39.9 mg, 78% yield), 0.1 mmol scale reaction. Flash column chromatography conditions: hexane:ethyl acetate = 3:1. **^{1}H NMR** (500 MHz, chloroform-*d*) 7.21 – 7.16 (comp, 5H), 7.11 (d, *J* = 8.6 Hz, 2H), 6.86 (d, *J* = 8.7 Hz, 2H), 5.17 (s, 2H), 3.98 (s, 2H), 3.81 (s, 3H), 1.14 (hept, *J* = 7.5 Hz, 3H), 0.96 (d, *J* = 7.5 Hz, 17H).**^{13}C NMR** (126 MHz, $CDCl_3$) δ 191.6, 186.6, 161.6, 159.8, 157.7, 135.7, 130.6, 128.9, 128.6, 126.7, 126.3, 68.5, 55.3, 29.7, 17.6. **HRMS** (ESI) calculated for $[M+Na]^+$: $C_{28}H_{37}NO_6Si$ m/z: 534.2282, observed: 534.2282.

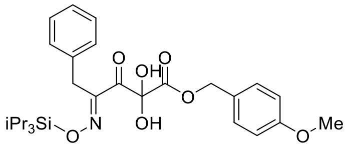

14g **4-Methoxybenzyl (*E*)-2,2-Dihydroxy-3-oxo-5-phenyl-4-[(triiso-propylsilyl)oxyimino]pentanoate**, **14g**. Colorless oil. **^{1}H NMR** (500 MHz, chloroform-*d*) δ 7.23 – 7.22 (comp, 7H), 6.83 (d, *J* = 8.7 Hz, 1H), 5.07 (s, 2H), 5.05 (s, 2H), 3.91 (s, 2H), 3.80 (s, 3H), 1.26 (hept, *J* = 7.6 Hz 3H), 1.05 (d, *J* = 7.6 Hz, 18H).**^{13}C NMR** (126 MHz, $CDCl_3$) δ 190.5, 168.7, 160.1, 158.7, 135.0, 129.7, 128.8, 128.4, 126.4, 125.9, 91.3, 68.2, 55.2, 28.6, 17.4, 11.6. **HRMS** (ESI) calculated for $[M+Na]^+$: $C_{28}H_{39}NO_7Si$ m/z: 552.2388, observed: 552.2388.

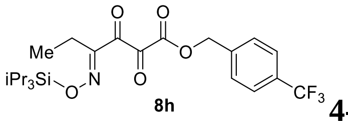

8h **4-(Trifluoromethyl)benzyl (*Z*)-2,3-Dioxo-4-[(triisopropylsilyl)oxy-imino]hexanoate**, **8h**. Colorless oil (**8h/14h** = 1:3.4; 29.2 mg, 70% yield), 0.1 mmol scale reaction. Flash column chromatography conditions: hexane:ethyl acetate = 3:1. **^{1}H NMR** (500 MHz, chloroform-*d*) δ 7.60 (d, *J* = 8.1 Hz, 2H), 7.38 (d, *J* = 8.0 Hz, 2H), 5.25 (s, 2H), 2.65 (q, *J* = 7.6 Hz, 2H), 1.27 (hept, *J* = 7.6, 3H), 1.09 (d, *J* = 7.6 Hz, 18H) 1.04 (t, *J* = 7.6 Hz, 1H). **^{13}C NMR** (126 MHz, $CDCl_3$) δ 191.4, 186.3, 165.1, 157.8 138.6, 131.0 (q, *J* = 32.3 Hz), 127.7, 126.1 (q, *J* = 271.6 Hz), 125.8 (q, *J* = 3.4 Hz), 67.4, 17.8, 17.6, 11.9 10.1. **HRMS** (ESI) calculated for $[M+H]^+$: $C_{23}H_{32}F_3NO_5Si$ m/z: 488.2075, observed: 488.2074.

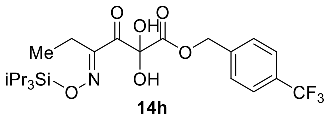

14h **4-(Trifluoromethyl)benzyl (*E*)-2,2-Dihydroxy-3-oxo-4-[(triiso-propylsilyl)oxyimino]hexanoate**, **14h**. Colorless oil. **^{1}H NMR** (500 MHz, chloroform-*d*) δ 7.64 (d, *J* = 8.1 Hz, 2H), 7.48 (d, *J* = 8.0 Hz, 2H), 5.32 (s, 2H), 5.09 (s, 2H), 2.60 (q, *J* = 7.6 Hz, 2H), 1.17 (hept, *J* = 7.5 Hz, 3H), 1.09 (t, *J* = 7.6 Hz, 3H), 0.99 (d, *J* = 7.5 Hz, 18H).**^{13}C NMR** (126 MHz, $CDCl_3$) δ 190.3, 168.7, 162.2, 137.9, 131.1 (q, *J* = 32.3 Hz), 128.6, 126.1 (q, *J* = 271.6 Hz),125.9 (q, *J* = 3.4 Hz), 91.5, 67.1, 17.6, 16.3, 11.8, 9.9. **HRMS** (ESI) calculated for $[M+H]^+$: $C_{23}H_{34}F_3NO_6Si$ m/z: 506.2180, observed: 506.2180.

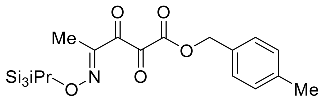

8k **4-Methylybenzyl (*E*)-2,3-Dioxo-4-[(triisopropylsilyl)oxyimino]pentanoate**, **8k**. Colorless oil (**8k/14k** = 1:1; 36.9 mg, 88% yield), 0.1 mmol scale reaction. Flash column chromatography conditions: hexane:ethyl acetate = 3:1. **^{1}H NMR** (500 MHz, chloroform-*d*) δ 7.24 (d, *J* = 8.0 Hz, 2H), 7.17 (d, *J* = 8.0 Hz, 2H), 5.15 (s, 2H), 2.35 (s, 3H), 2.05 (s, 3H), 1.33 – 1.24 (comp, 3H), 1.08 (d, *J* = 7.5 Hz, 18H).**^{13}C NMR** (126 MHz, $CDCl_3$) δ191.8, 186.7, 160.7, 157.7, 138.6, 131.0, 129.4, 128.1, 68.7, 21.4, 17.8, 11.9, 9.8. **HRMS** (ESI) calculated for $[M+H]^+$: $C_{22}H_{33}NO_5Si$ m/z: 420.2201, observed: 420.2203.

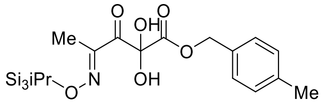

14k **4-Methylbenzyl (*E*)-2,2-Dihydroxy-3-oxo-4-[(triisopropylsilyl)-oxyimino]pentanoate**, **14k**. Colorless oil. **^{1}H NMR** (500 MHz, chloroform-*d*) δ 7.15 (s, 4H), 5.23 (s, 2H), 5.09 (s, 2H), 2.33 (s, 3H), 2.05 (s, 3H), 1.21 – 1.14 (comp, 2H), 1.00 (d, *J* = 7.4 Hz, 18H).**^{13}C NMR** (126 MHz, $CDCl_3$) δ 190.7, 168.9, 158.1, 139.0, 131.6, 129.5, 128.9, 91.5, 68.4, 21.3, 17.6, 11.8, 8.2. **HRMS** (ESI) calculated for $[M+H]^+$: $C_{22}H_{35}NO_6Si$ m/z: 438.2306, observed: 438.2309.

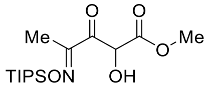

13a **Methyl (*Z*)-2-Hydroxy-3-oxo-4-[(triisopropylsilyl)oxyimino]pentanoate**, **13a**. Colorless oil. **^{1}H NMR** (500 MHz, acetonitrile-*d*$_3$) δ 5.38 (d, *J* = 8.0 Hz, 1H), 4.16 (d, *J* = 8.0 Hz, 1H), 3.69 (s, 3H), 2.02 (s, 3H), 1.33 (hept, *J* = 14.5, 7.2 Hz, 5H), 1.13 (d, *J* = 7.2 Hz, 18H).**^{13}C NMR** (126 MHz, CD_3CN) δ 193.1, 169.1, 159.1, 73.3, 52.2, 17.0, 17.0, 11.5.

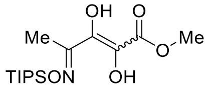

12a **Methyl (4*Z*)-2,3-Dihydroxy-4-[(triisopropylsilyl)oxyimino]pent-2-enoate**, **12a**. Colorless oil. **^{1}H NMR** (300 MHz, chloroform-*d*) δ 10.17 (s, 1H), 9.94 (br, 1H), 3.94 (s, 3H), 2.20 (s, 3H), 1.29 (hept, *J* = 7.2 Hz, 3H), 1.10 (d, *J* = 7.2 Hz, 18H).**^{13}C NMR** (75 MHz, $CDCl_3$) δ 169.2, 163.1, 142.5, 127.9, 52.5, 17.7, 11.7, 9.9. **HRMS** (ESI) calculated for $[M+Na]^+$: $C_{15}H_{29}NO_5Si$ m/z: 354.1707, observed: 354.1707

2.7 Crystallographic data

Crystallographic data and structure refinement for compound 9h

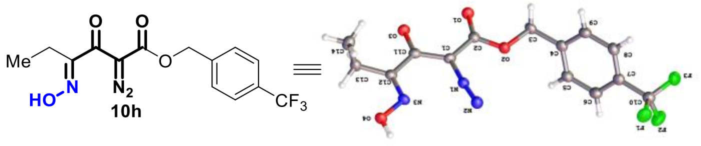

CCDC 2062029

Supplementary Fig. 8. ORTEP drawing of **10h** showing thermal ellipsoids at the 50% probability level.

Single crystals of $C_{14}H_{12}F_3N_3O_4$(1) were prepared by slow evaporation of a MeOH/DCM solution. A suitable colorless plank-like crystal, with dimensions of 0.191 mm × 0.120 mm × 0.042 mm, was mounted in paratone oil onto a nylon loop. All data were collected at 100.0(1) K, using a XtaLAB Synergy/ Dualflex, HyPix fitted with CuKα radiation (λ = 1.54184 Å). Data collection and unit cell refinement were performed using CrysAlisPro software.[2] The total number of data were measured in the 5.7° < 2θ < 153.0° for compound **10h**, using ω scans. Data processing and absorption correction, giving minimum and maximum transmission factors (0.655, 1.000 for compound **10h** were accomplished with CrysAlisPro[2] and SCALE3 ABSPACK[3]. The structure, using Olex2[4], was solved with the ShelXT[5] structure solution program using direct methods and refined (on F2) with the ShelXL[6] refinement package using full-matrix, least-squares techniques. All non-hydrogen atoms were refined with anisotropic displacement parameters. All hydrogen atom positions were determined by geometry and refined by a riding model.

Supplementary Table 6: Crystallographic data and structure refinement for **10h**

Identification code	Hpd294
Empirical formula	$C_{14}H_{12}F_3N_3O_4$
Formula weight	343.27
Crystal system	Triclinic
Space group	*P-1*
a (Å)	6.3889(3)
b (Å)	7.3632(2)
c (Å)	15.7141(7)
α (°)	88.345(2)
β (°)	80.040(3)
γ (°)	84.112(2)
Volume (Å^3)	724.21(5)
Z	2

ρ (calc.)	1.574
λ	1.54184
Temp. (K)	100.0(1)
F(000)	352
μ (mm^{-1})	1.241
T_{min}, T_{max}	0.655, 1.000
$2\theta_{range}$ (°)	5.7 to 153.0
Reflections collected	13179
Independent reflections	2890 [R(int) = 0.0460]
Completeness	99.4%
Data / restraints / parameters	2890 / 0 / 219
Observed data [I > 2σ(I)]	2491
wR(F^2 all data)	0.1336
R(F obsd data)	0.0534
Goodness-of-fit on F^2	1.09
largest diff. peak and hole (e $Å^{-3}$)	0.43 / -0.36

$$wR_2 = \{ \Sigma [w(F_o^2 - F_c^2)^2] / \Sigma [w(F_o^2)^2] \}^{1/2}$$

$$R_1 = \Sigma ||F_o| - |F_c|| / \Sigma |F_o|$$

Crystallographic data and structure refinement for compound 10

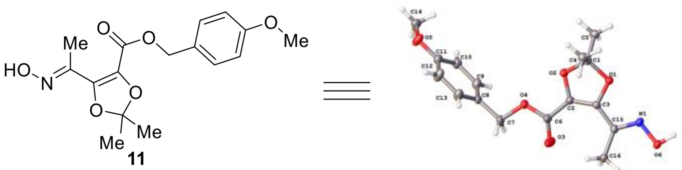

11

CCDC 2181552

Supplementary Fig. 9. ORTEP drawing of **11** showing thermal ellipsoids at the 50% probability level.

Single crystals of of $C_{16}H_{19}NO_6$ were prepared by slow evaporation of a DCM/hexane solution. A suitable colorless plank-like crystal, with dimensions of 0.123 mm × 0.090 mm × 0.033 mm, was mounted in paratone oil onto a nylon loop. All data were collected at 100.0(1) K, using a XtaLAB Synergy/ Dualflex, HyPix fitted with CuKα radiation (λ = 1.54184 Å). Data collection and unit cell refinement were performed using CrysAlisPro software.[2] The total number of data were measured in the 6.2° < 2θ < 152.8° for compound **11**, using ω scans. Data processing and absorption correction, giving minimum and maximum transmission factors (0.808, 1.000 for compound **11**) were accomplished with CrysAlisPro[2] and SCALE3 ABSPACK[3]. The structure, using Olex2[4], was solved with the ShelXT[5] structure solution program using direct methods and refined (on F2) with the ShelXL[6] refinement package using full-matrix, least-squares techniques. All non-hydrogen atoms were refined with anisotropic displacement parameters. All hydrogen atom positions were determined by geometry and refined by a riding model. the hydroxyl hydrogen atom on the oxygen atom, O6, was determined by electron density plot.

Supplementary Table 7: Crystallographic data and structure refinement for **11**

Identification code	Hpd300d
Empirical formula	$C_{16}H_{19}NO_6$
Formula weight	321.32
Crystal system	Monoclinic
Space group	$P2_1/c$
a (Å)	5.6390(1)
b (Å)	9.6551(3)
c (Å)	28.5560(7)
α (°)	90
β (°)	95.007(2)
γ (°)	90

Volume (Å^3)	1548.80(7)
Z	4
ρ (calc.)	1.378
λ	1.54184
Temp. (K)	100.0(1)
F(000)	680
μ (mm^{-1})	0.890
T_{min}, T_{max}	0.808, 1.000
$2\theta_{range}$ (°)	6.2 to 152.8
Reflections collected	18132
Independent reflections	3126 [R(int) = 0.0549]
Completeness	99.8%
Data / restraints / parameters	3126 / 0 / 215
Observed data [I > 2σ(I)]	2603
$wR(F^2$ all data)	0.1325
$R(F$ obsd data)	0.0502
Goodness-of-fit on F^2	1.04
largest diff. peak and hole (e Å^{-3})	0.78 / -0.62

$$wR_2 = \{ \Sigma [w(F_o^2 - F_c^2)^2] / \Sigma [w(F_o^2)^2] \}^{1/2}$$

$$R_1 = \Sigma ||F_o| - |F_c|| / \Sigma |F_o|$$

2.8 Computational Details

All calculations were performed using the Gaussian 16, Revision B.01 package.[7] All structures were optimized in gas phase at the (U)BP86 level[8] of theory in combination with D3(BJ) dispersion corrections,[9] and the 6-31G(d) basis set[10] was used for all atoms. Analytical frequency calculations were carried out at the same level of theory in order to confirm each stationary point as either an intermediate (no imaginary frequencies) or a transition state (only one imaginary frequency). Key transition-state structures were confirmed to connect corresponding reactants and products by intrinsic reaction coordinate (IRC) calculations.[11] The electronic energy in acetonitrile was then refined using 6-311++G(d,p) basis set[12] for all atoms at the (U)BP86 level with D3(BJ) dispersion corrections on the optimized geometries, in which the solvent effects (ε = 35.688 for MeCN, ε = 8.93 for DCM) were evaluated by SMD solvation model.[13] Standard state concentrations of 1.0 mol/L were used for all species in calculations. The given Gibbs free energies in acetonitrile were calculated in Supplementary **Table 8** according to the formula: $G_{sol} = TCG + E_{sol} + 1.89$ (kcal/mol). The CYL View software was employed to show the 3D structures of the studied species.[14]

2.9 Computed Reaction Pathways

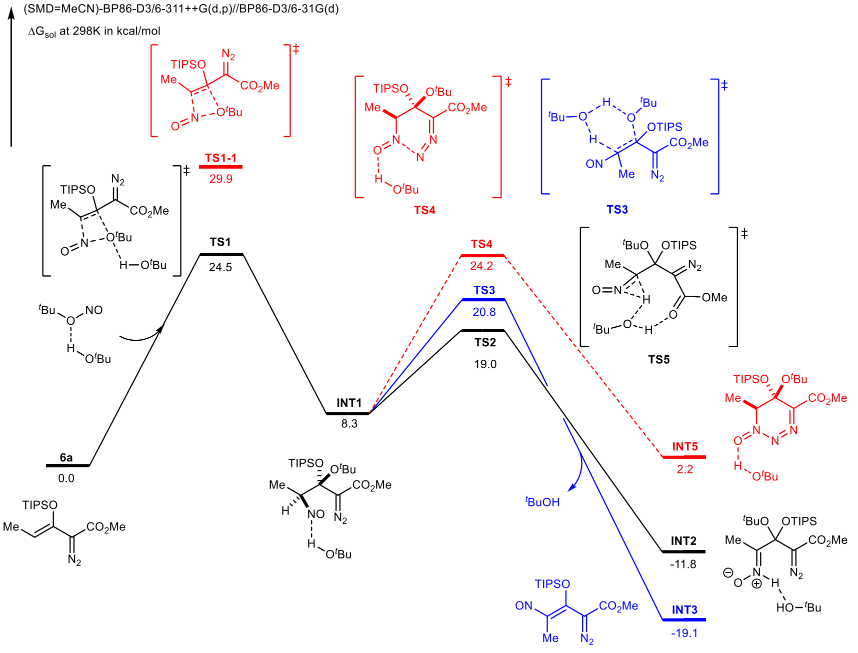

Supplementary Fig. 10. Relative free-energy profile for the reaction of enol diazoacetate **6a** with TBN.

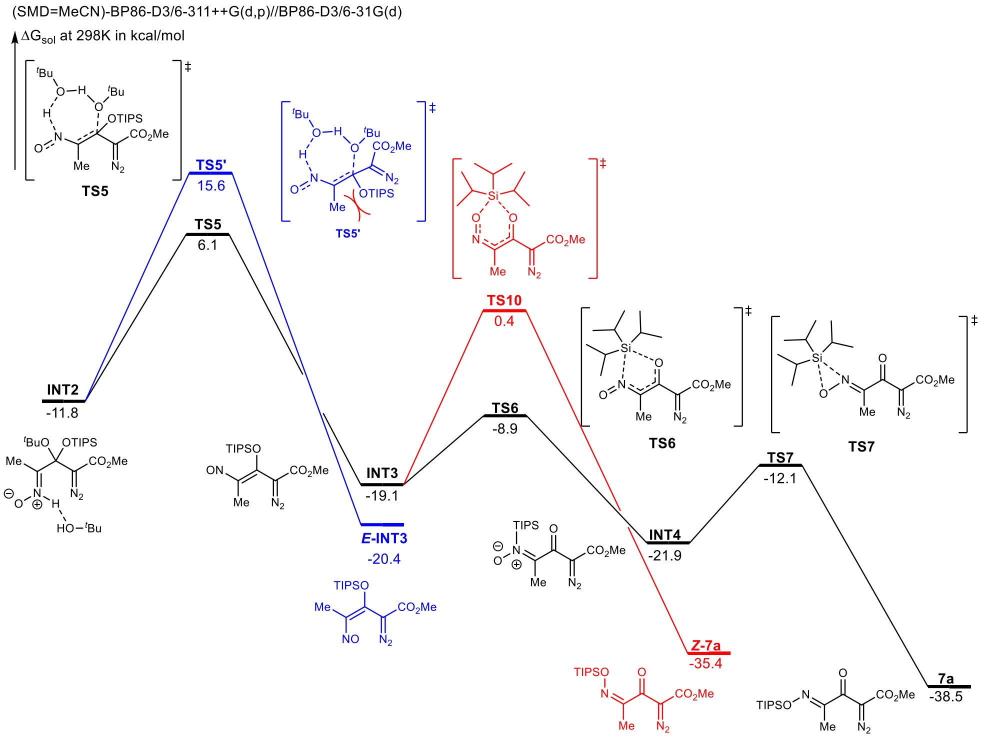

Supplementary Fig. 11. Relative free-energy profile for different reaction pathways from **INT2**.

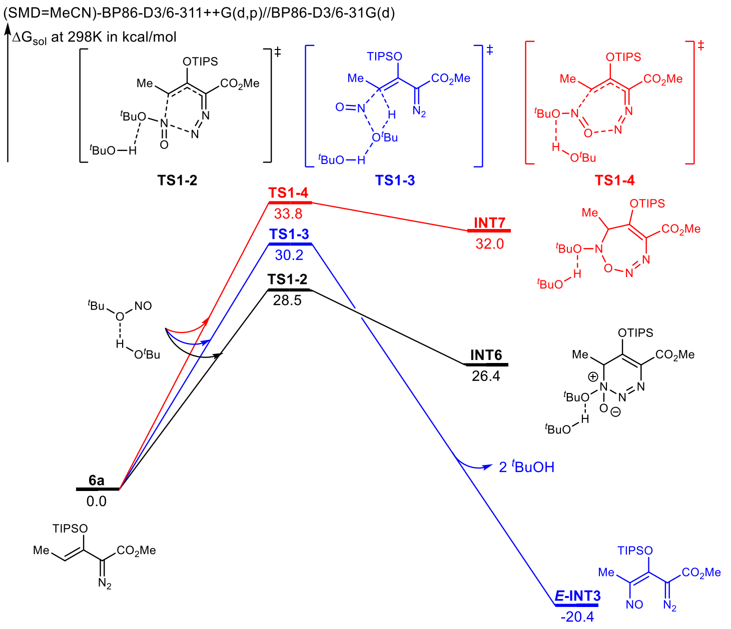

Supplementary Fig. 12. Relative free-energy profile for different reaction pathways of enol-diazoacetate **6a** with TBN.

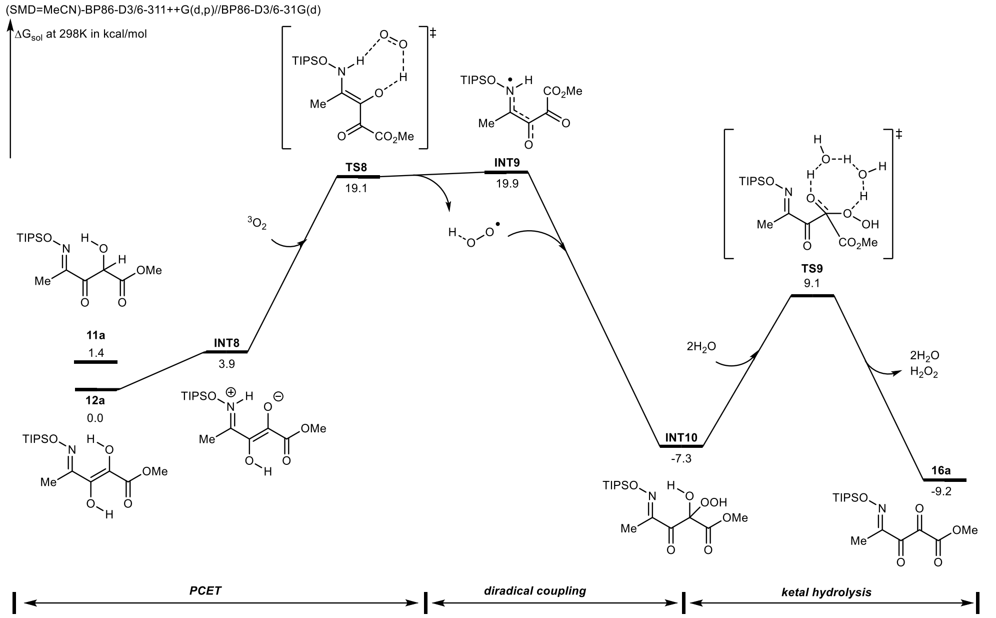

Supplementary Fig. 13. Relative free-energy profile for oxidation of 11a with oxygen.

2.10 Computed Energy of all Stationary Points

Supplementary Table S8. Calculated Energies of all Stationary Points for Reaction Pathways.

Thermal correction to Gibbs free energies (***TCG***, in Hartree), thermal correction to enthalpies (***TCH***, in Hartree), sum of electronic and thermal free energies (***G***, in Hartree), Sum of electronic and thermal enthalpies (***H***, in Hartree), and single point energies in dichloromethane or acetonitrile computed at the (U)BP86-D3/6-311++G(d,p) level (***E***, in Hartree).

Name	***TCG***/a.u.	***TCH***/a.u.	***G***/a.u.	***H***/a.u.	***E***/a.u.
**6a**	0.349057	0.435642	-1212.872836	-1212.786251	-1213.523191
**TBN**	0.096473	0.138518	-362.890662	-362.848618	-363.103709
**TS1-1**	0.469304	0.574514	-1575.716942	-1575.611731	-1576.599911
**tBuOH**	0.102995	0.139136	-233.569217	-233.533076	-233.763068
**TBN-tBuOH**	0.215506	0.281005	-596.455006	-596.389507	-596.872890
**TS1**	0.595941	0.717808	-1809.296612	-1809.174744	-1810.385424
**INT1**	0.595683	0.719896	-1809.322007	-1809.197793	-1810.410954
**TS2**	0.599513	0.716247	-1809.296928	-1809.180194	-1810.397753
**INT2**	0.602375	0.722225	-1809.356420	-1809.23657	-1810.449611
**TS3**	0.596142	0.714417	-1809.300617	-1809.182342	-1810.391483
**INT3**	0.343886	0.434981	-1342.204917	-1342.113822	-1342.888701
**TS4**	0.600824	0.718974	-1809.300773	-1809.182622	-1810.390761
**INT5**	0.601151	0.721827	-1809.332729	-1809.212053	-1810.426115
**TS5**	0.592039	0.712931	-1809.323613	-1809.202721	-1810.410893
**TS5'**	0.594626	0.714532	-1809.308971	-1809.189065	-1810.398257
***E*-INT3**	0.344946	0.435012	-1342.204825	-1342.114759	-1342.891770
**TS6**	0.345811	0.434576	-1342.187489	-1342.098723	-1342.874435
**INT4**	0.343740	0.435412	-1342.204003	-1342.112331	-1342.893025
**TS7**	0.343204	0.433480	-1342.193463	-1342.103188	-1342.876857
**7a**	0.341026	0.434632	-1342.232131	-1342.138525	-1342.916798
**TS10**	0.345651	0.433659	-1342.176145	-1342.088137	-1342.859405
***Z*-7a**	0.342573	0.434425	-1342.229345	-1342.137493	-1342.913439
**TS1-2**	0.592802	0.717815	-1809.290632	-1809.165618	-1810.375914
**INT6**	0.596931	0.720309	-1809.293185	-1809.169806	-1810.383284
**TS1-3**	0.583851	0.712526	-1809.283710	-1809.155034	-1810.364180
**TS1-4**	0.592084	0.717459	-1809.285562	-1809.160187	-1810.366751
**INT7**	0.599609	0.720067	-1809.291100	-1809.170642	-1810.377196
**13a**	0.360390	0.451895	-1309.164036	-1309.072531	-1309.876524
**13a-1**	0.362423	0.451653	-1309.168124	-1309.078893	-1309.880753
**INT8**	0.359931	0.450735	-1309.159942	-1309.069137	-1309.872094

**$^{3}O_2$**	-	0.006863	-150.347164	-150.323850	-150.380969
**TS8**	0.355093	0.453850	-1459.476412	-1459.377655	-1460.237416
**INT9**	0.345813	0.439270	-1308.547113	-1308.453657	-1309.245917
**HOO•**	-	0.017160	-150.917781	-150.891750	-150.975139
**INT10**	0.364970	0.460958	-1459.522295	-1459.426307	-1460.289333
**H_2O**	0.002882	0.024357	-76.406776	-76.385301	-76.467103
**TS9**	0.406483	0.509219	-1612.335832	-1612.233096	-1613.227073
**8a**	0.337495	0.428433	-1307.969861	-1307.878923	-1308.650822
**H_2O_2**	0.002889	0.029500	-151.539450	-151.512838	-151.619988

2.11 Copies of NMR Spectra

Supplementary Fig. 14. **^{1}H-NMR (300 MHz, $CDCl_3$, 298 K) of compound 7a.**

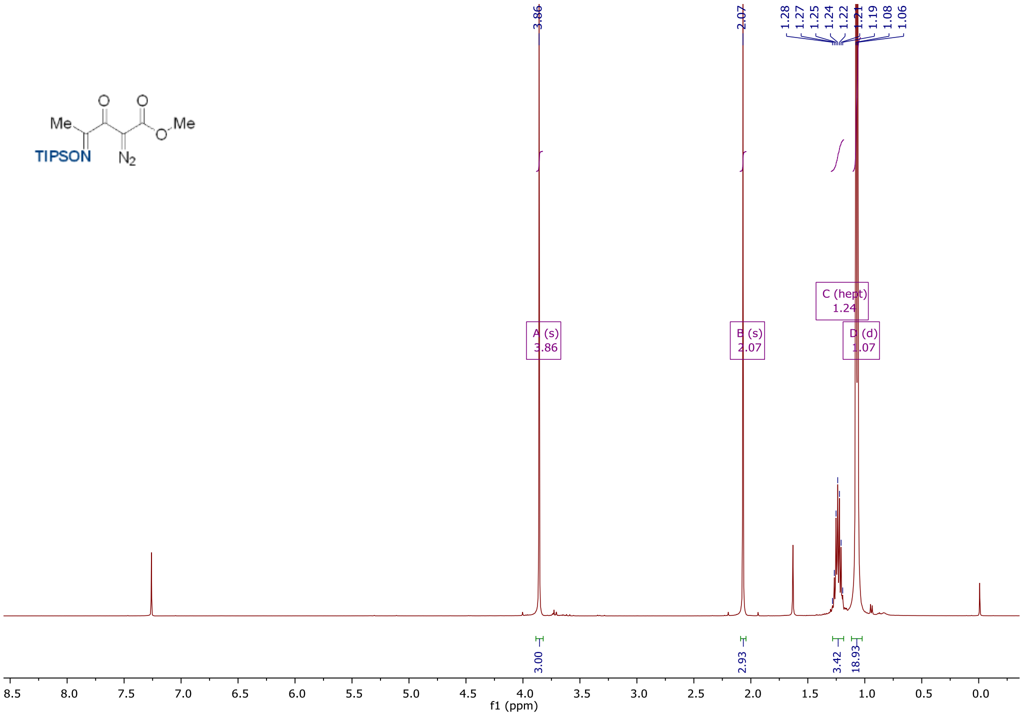

Supplementary Fig. 15. **^{13}C-NMR (75 MHz, $CDCl_3$, 298 K) of compound 7a**

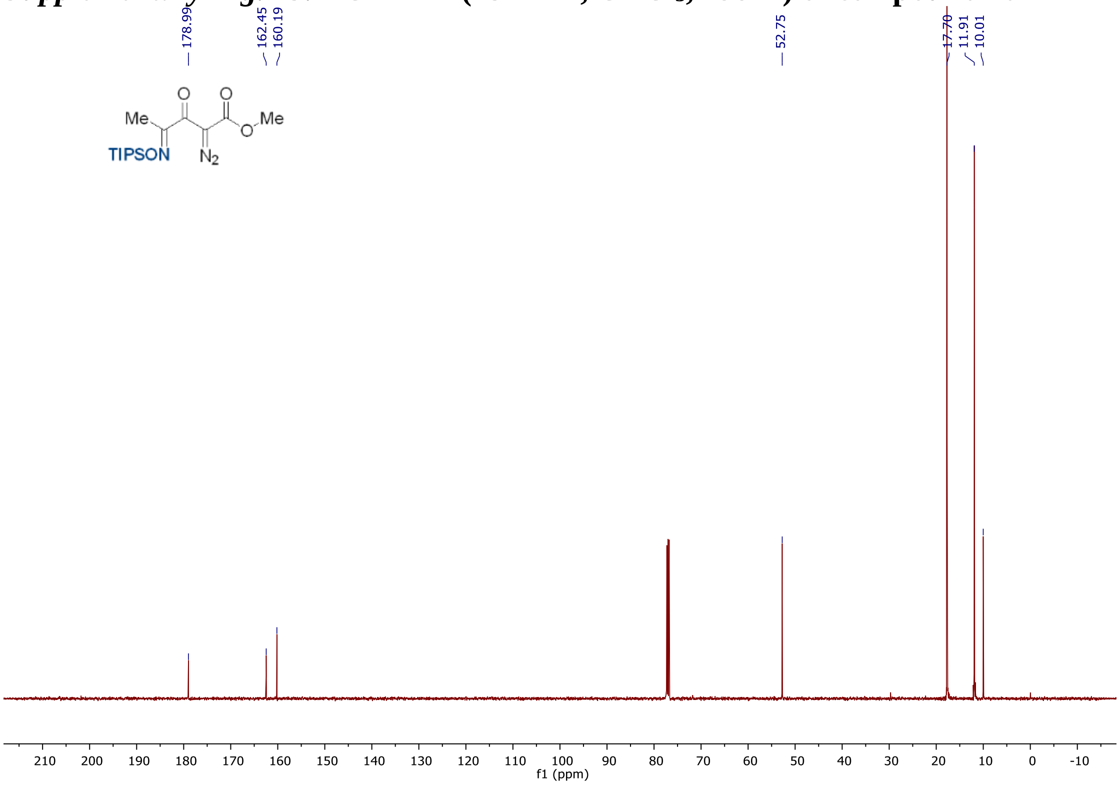

Supplementary Fig. 16. **^{15}N-NMR (75 MHz, $CDCl_3$, 298 K) of compound 7a**

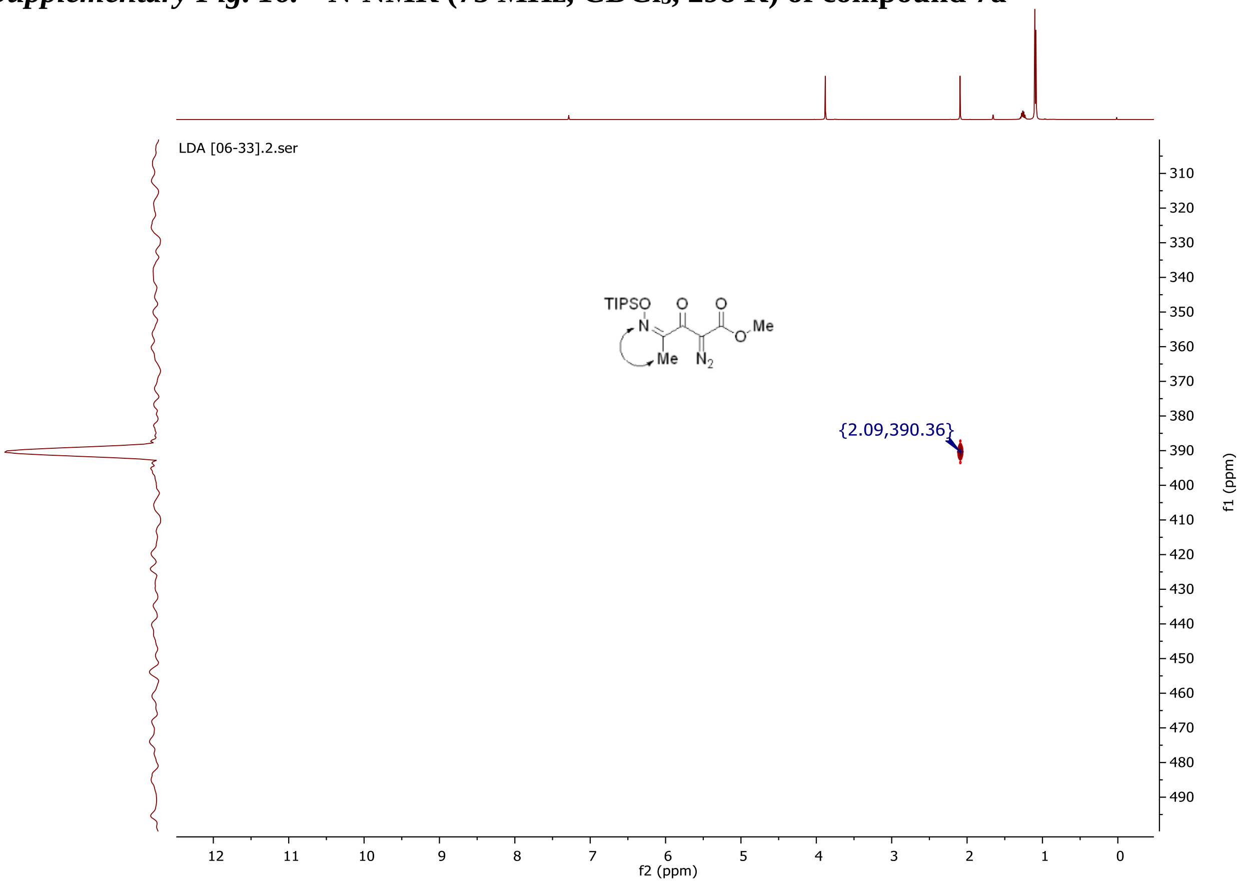

Supplementary Fig. 17. **^{1}H-NMR (500 MHz, $CDCl_3$, 298 K) of compound 7b**

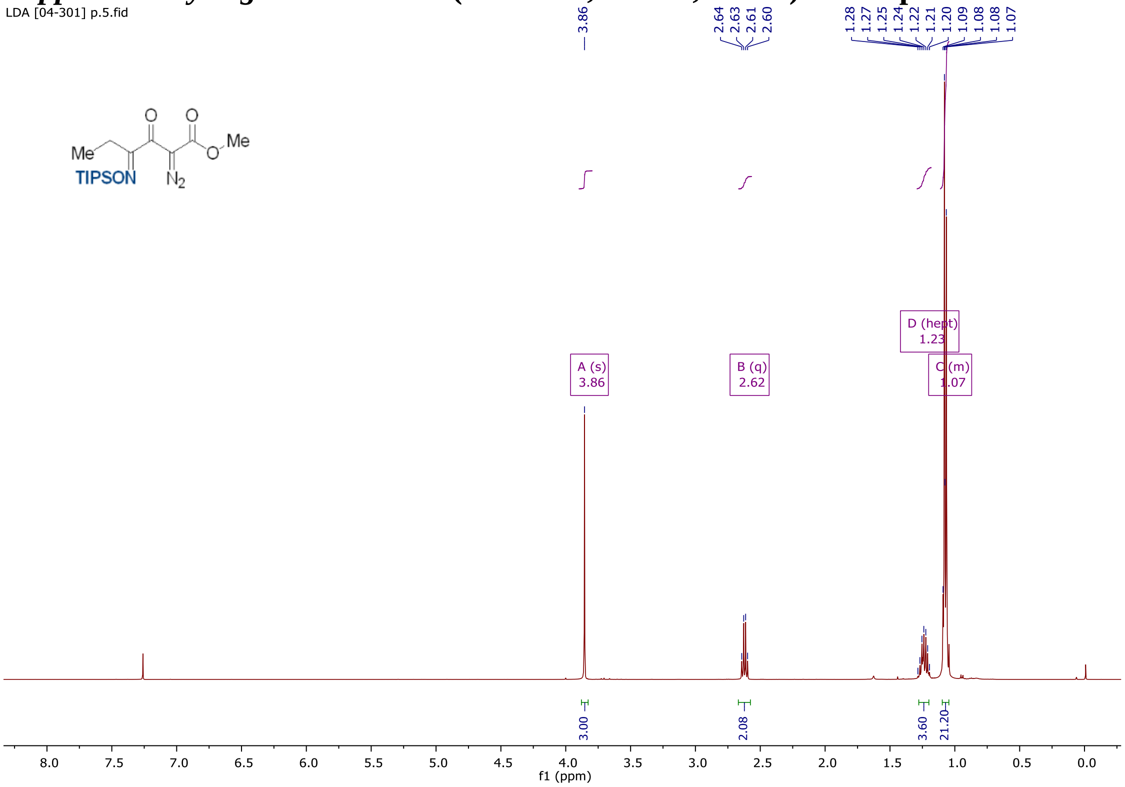

Supplementary Fig. 18. **^{13}C-NMR (126 MHz, $CDCl_3$, 298 K) of compound 7b**

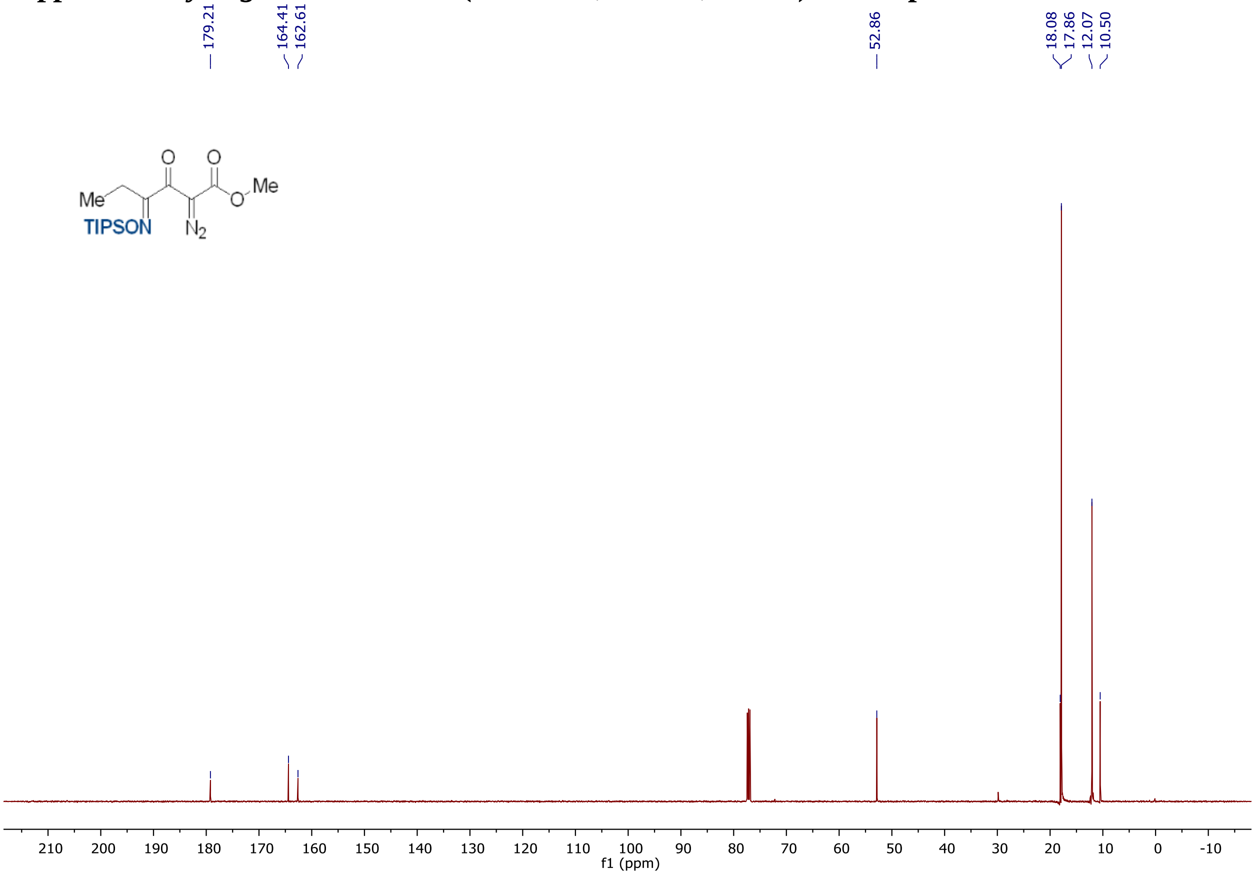

Supplementary Fig. 19. ^{1}H-NMR (500 MHz, $CDCl_3$, 298 K) of compound 7c

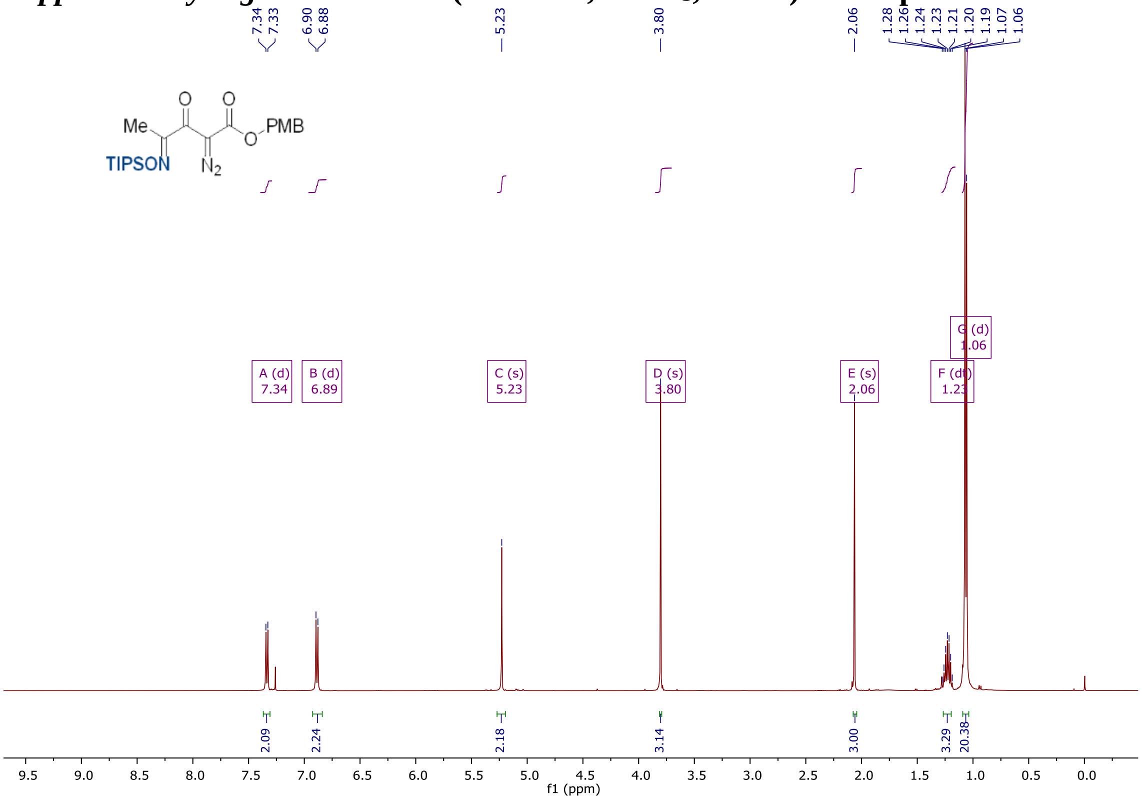

Supplementary Fig. 20. ^{13}C-NMR (126 MHz, $CDCl_3$, 298 K) of compound 7c

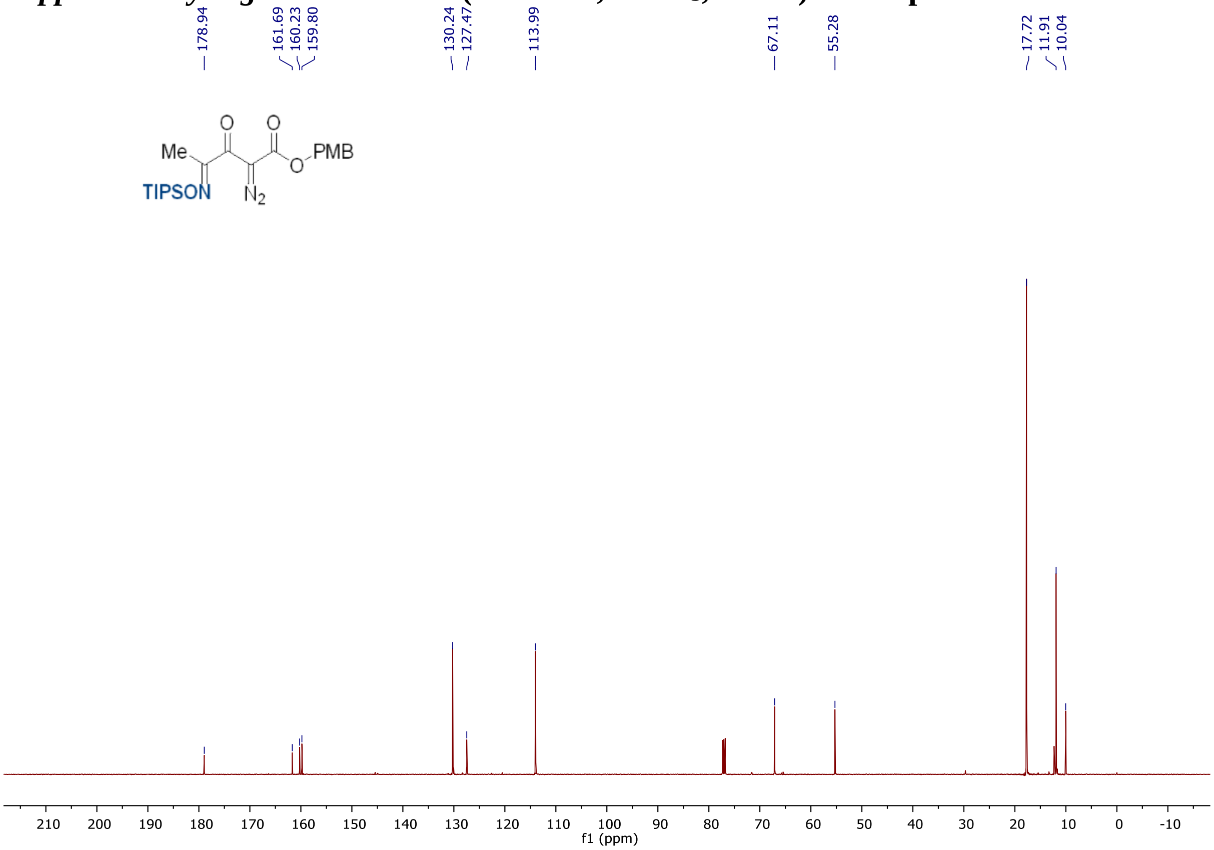

Supplementary Fig. 21. **^{1}H-NMR (500 MHz, CDCl₃, 298 K) of compound 7d**

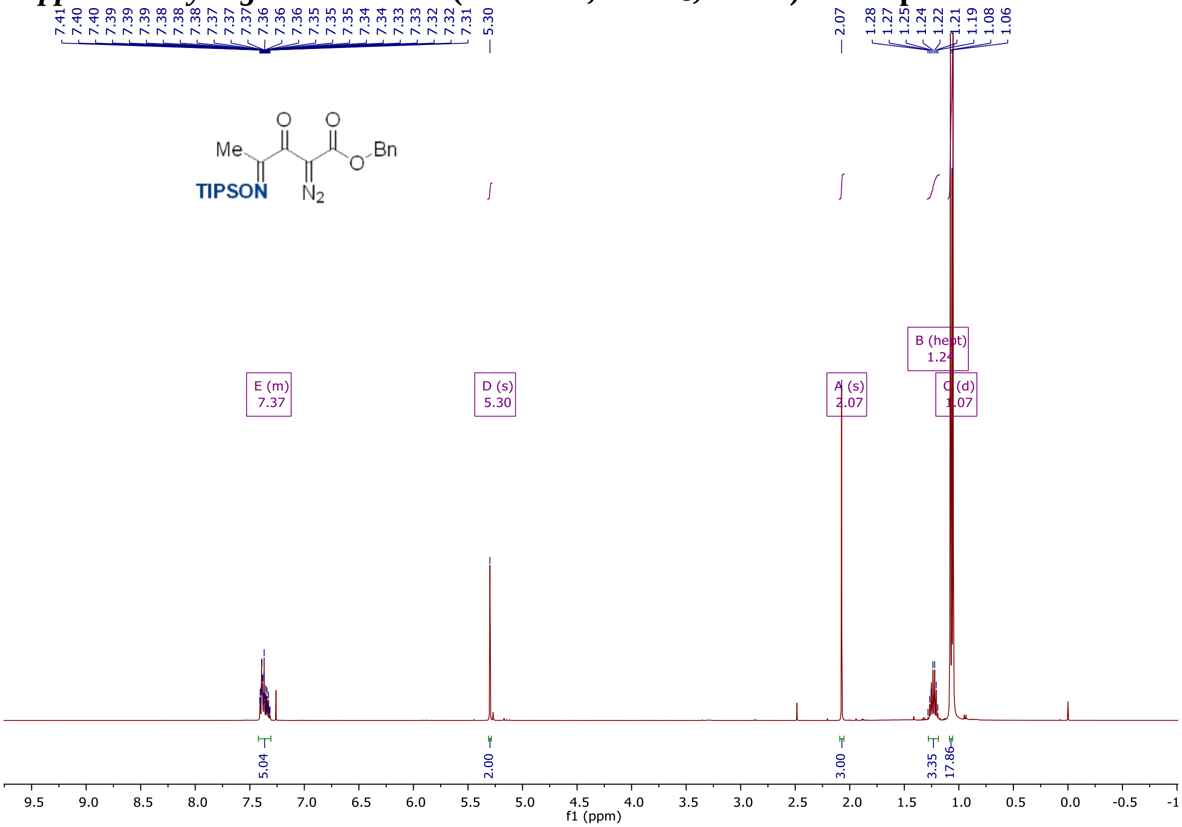

Supplementary Fig. 22. **^{13}C-NMR (126 MHz, CDCl₃, 298 K) of compound 7d**

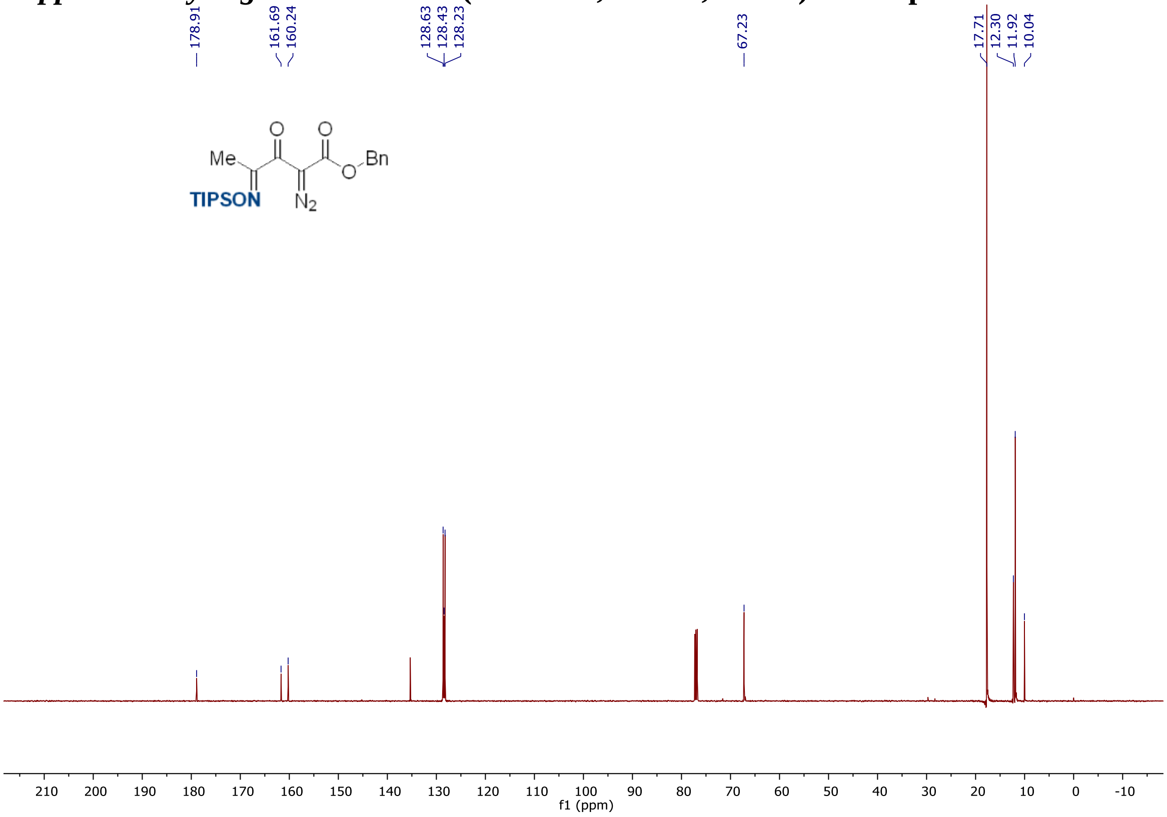

Supplementary Fig. 23. ^{1}H-NMR (500 MHz, $CDCl_3$, 298 K) of compound 7e

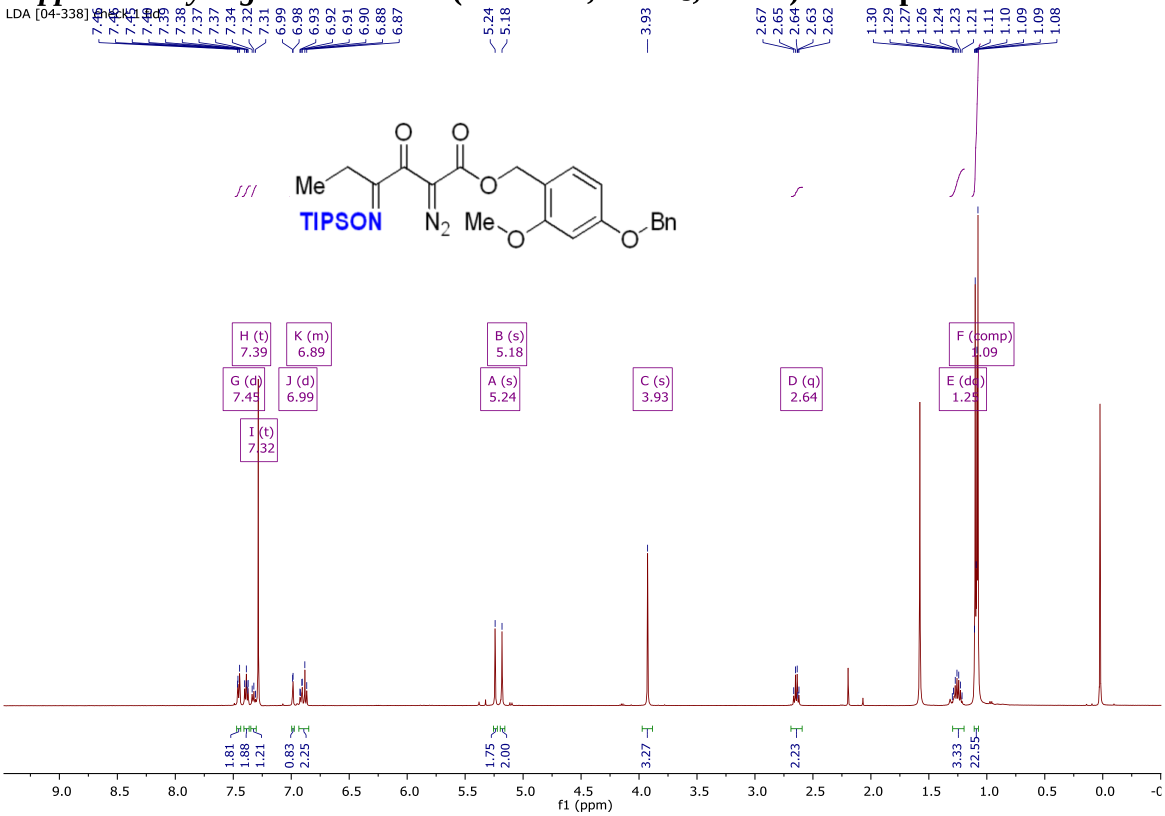

Supplementary Fig. 24. ^{13}C-NMR (126 MHz, $CDCl_3$, 298 K) of compound 7e

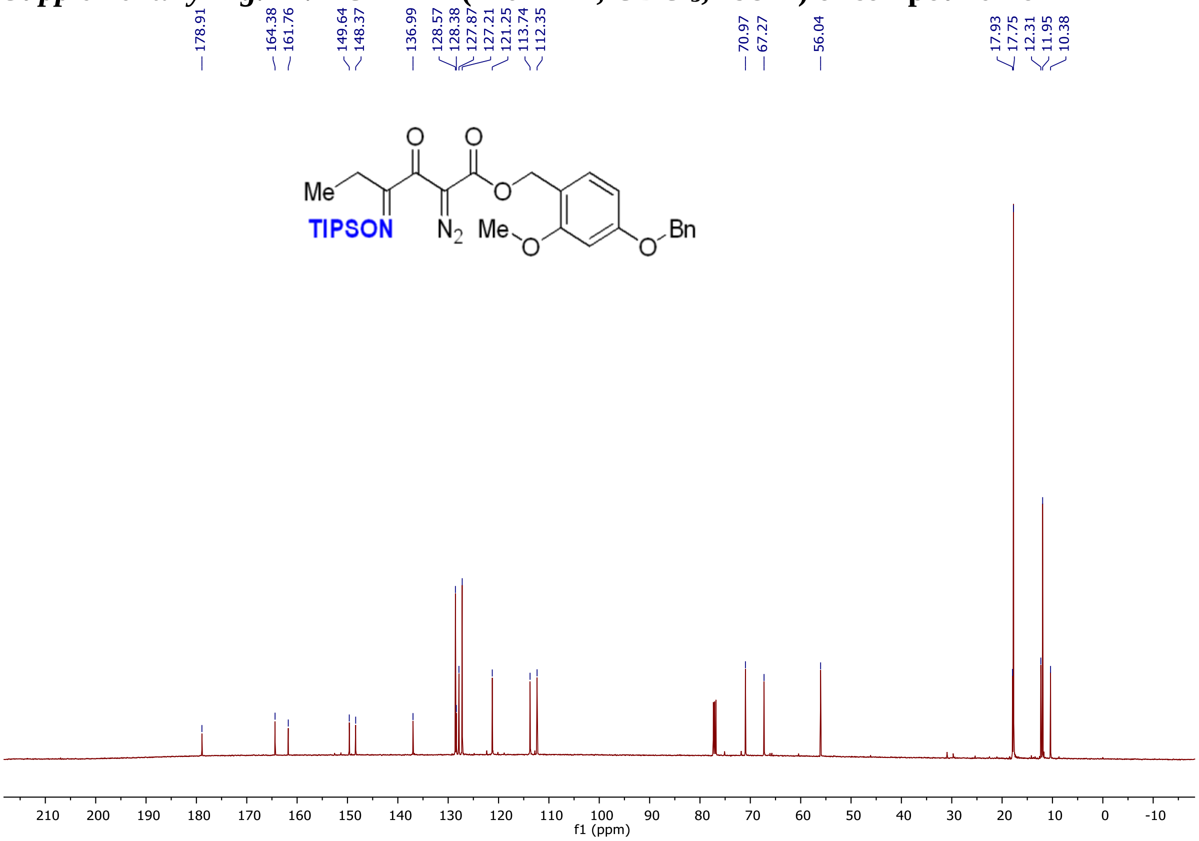

Supplementary Fig. 25. **^{1}H-NMR (500 MHz, CDCl$_3$, 298 K) of compound 7f**

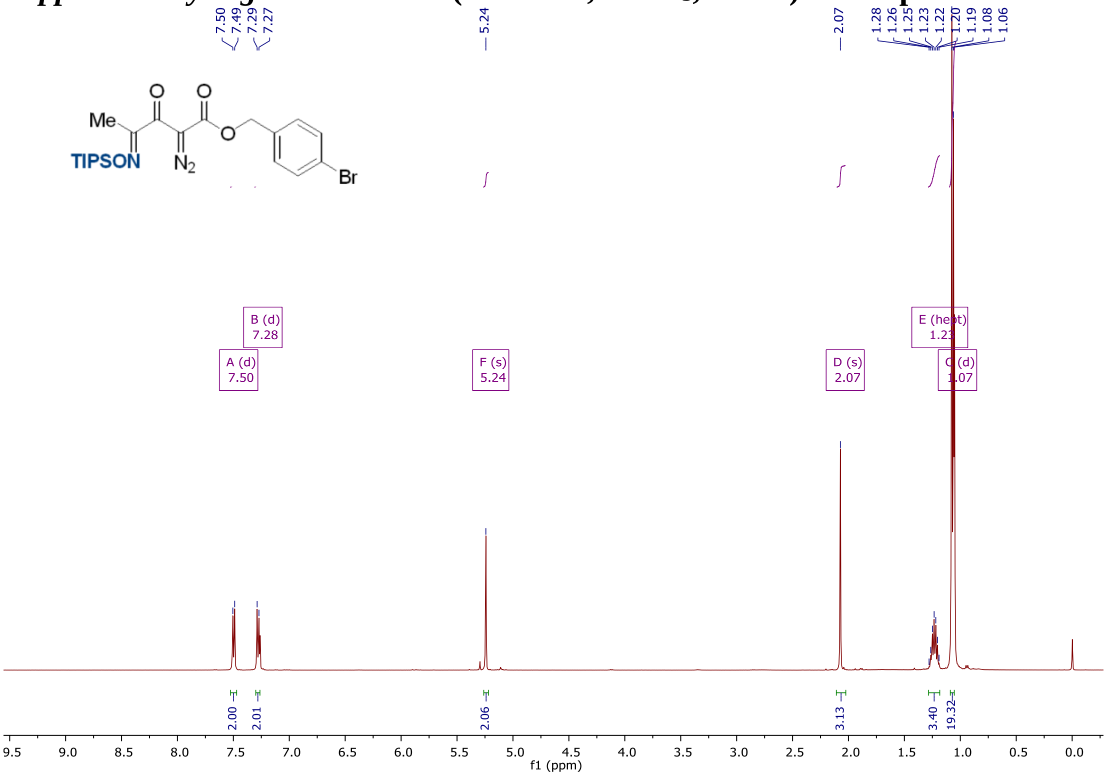

Supplementary Fig. 26. **^{13}C-NMR (126 MHz, CDCl$_3$, 298 K) of compound 7f**

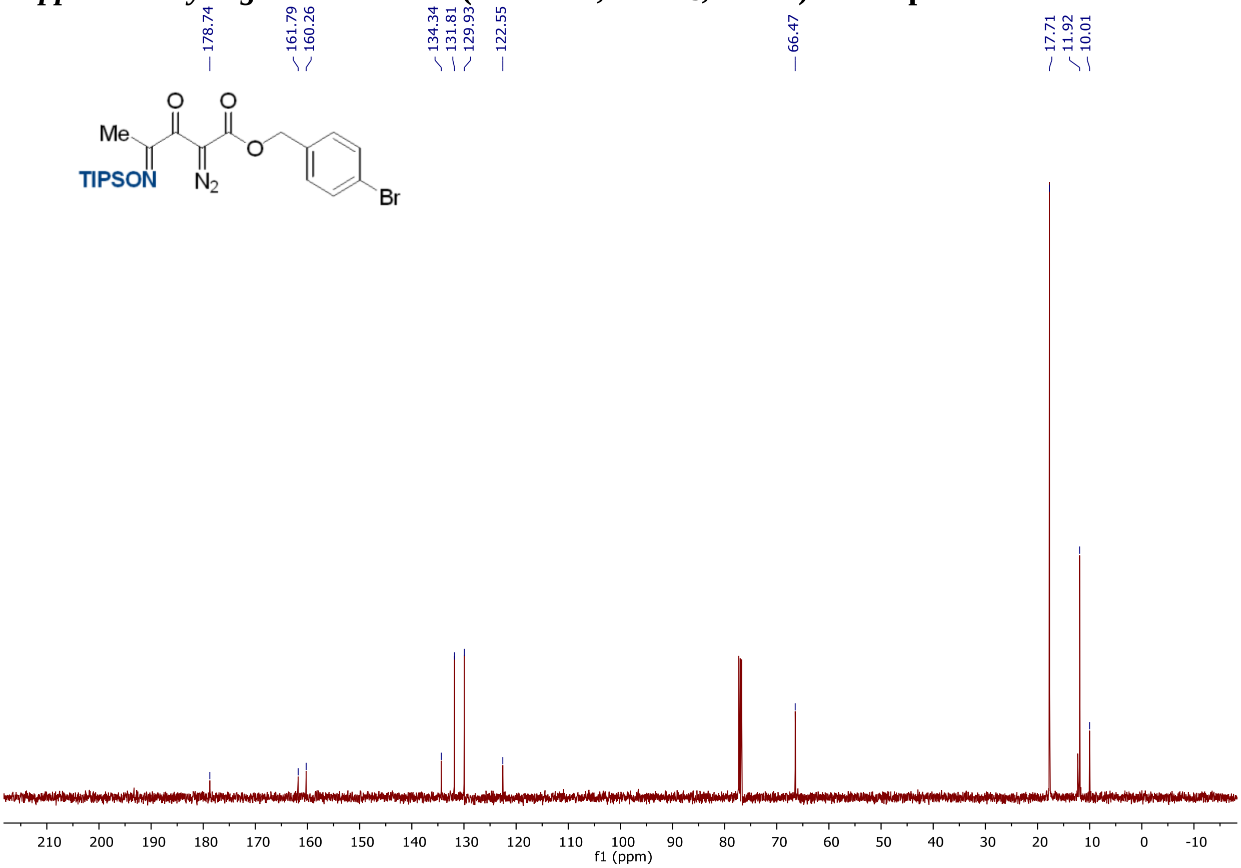

Supplementary Fig. 27. **^{1}H-NMR (500 MHz, $CDCl_3$, 298 K) of compound 7g**

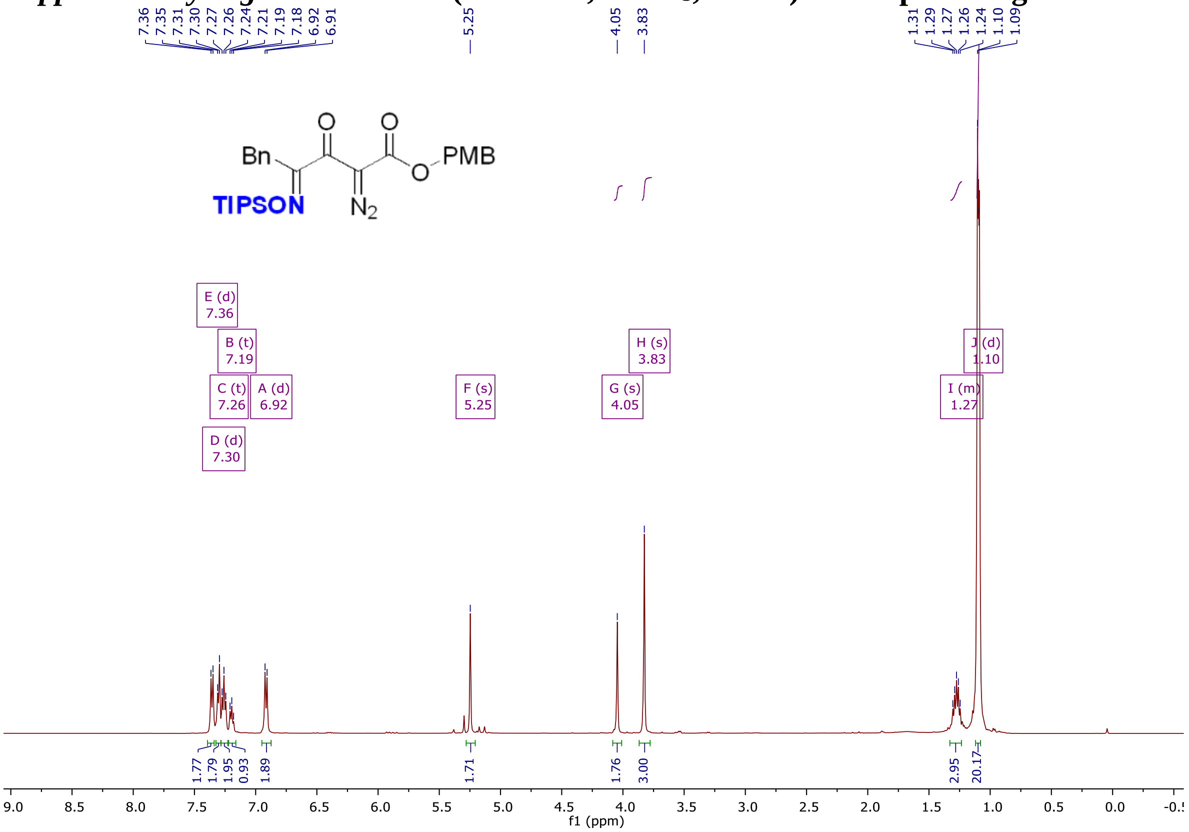

Supplementary Fig. 28. **^{13}C-NMR (126 MHz, $CDCl_3$, 298 K) of compound 7g**

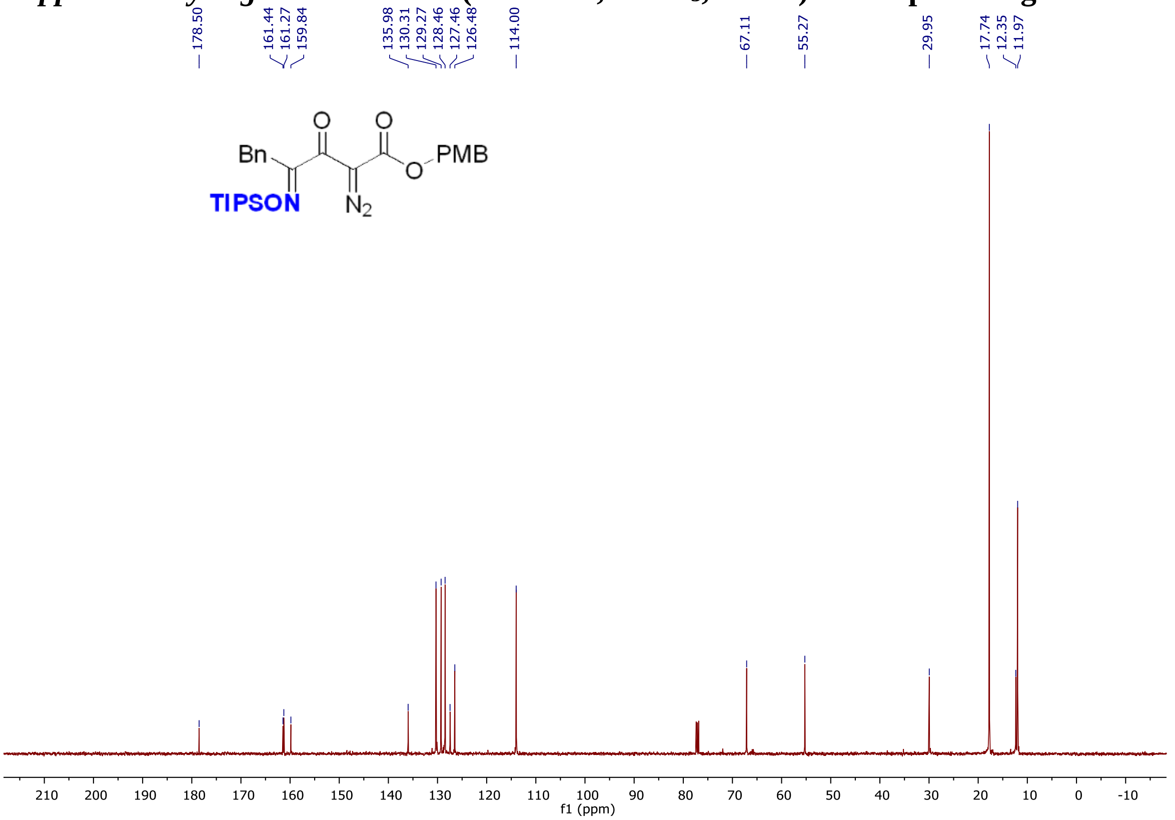

Supplementary Fig. 29. **^{1}H-NMR (500 MHz, CDCl$_3$, 298 K) of compound 7h**

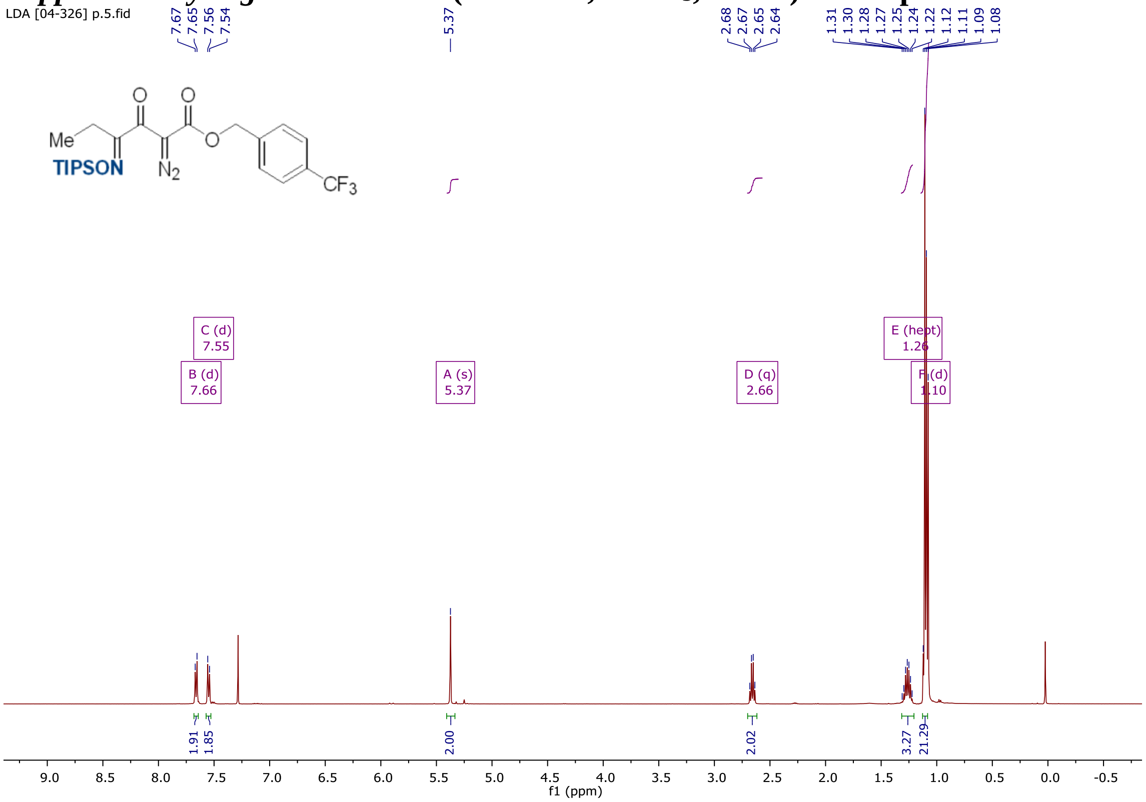

Supplementary Fig. 30. **^{13}C-NMR (126 MHz, CDCl$_3$, 298 K) of compound 7h**

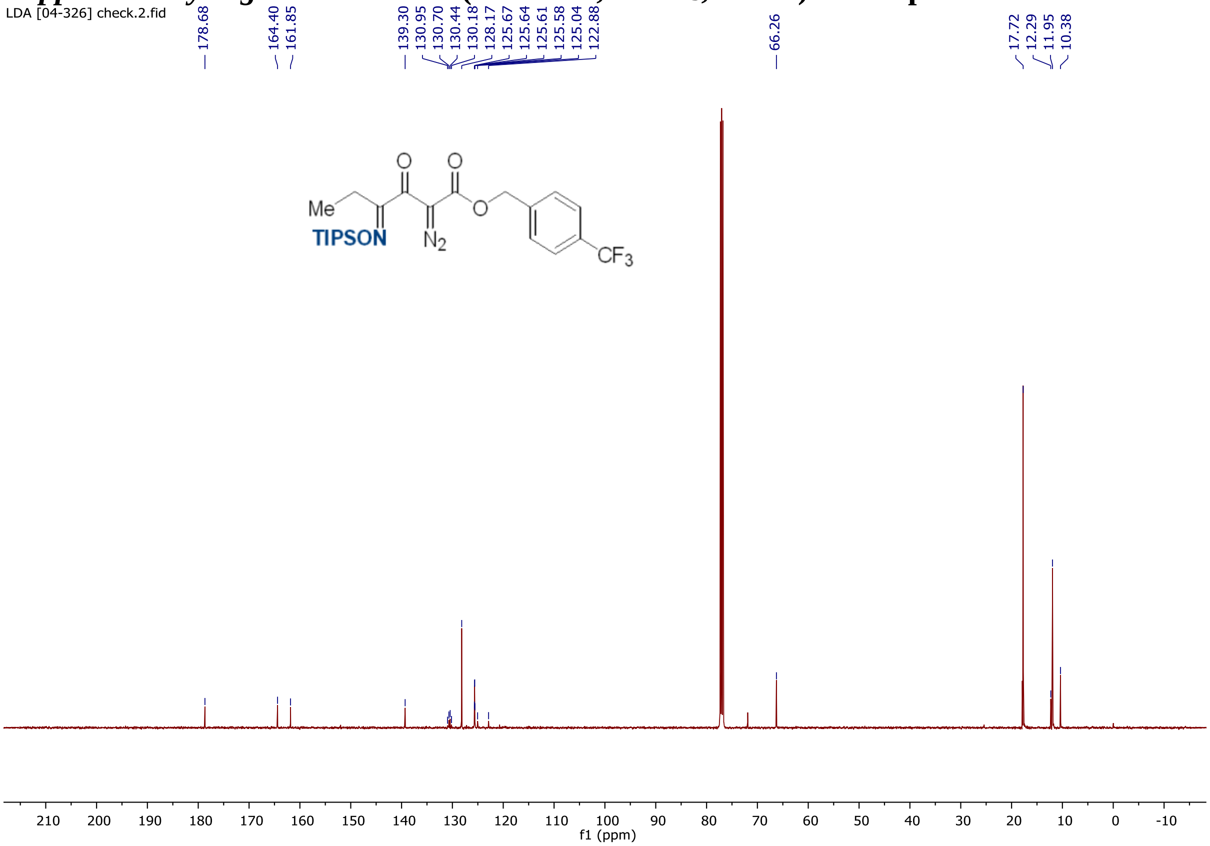

Supplementary Fig. 31. **^{1}H-NMR (500 MHz, CDCl3, 298 K) of compound 7i**

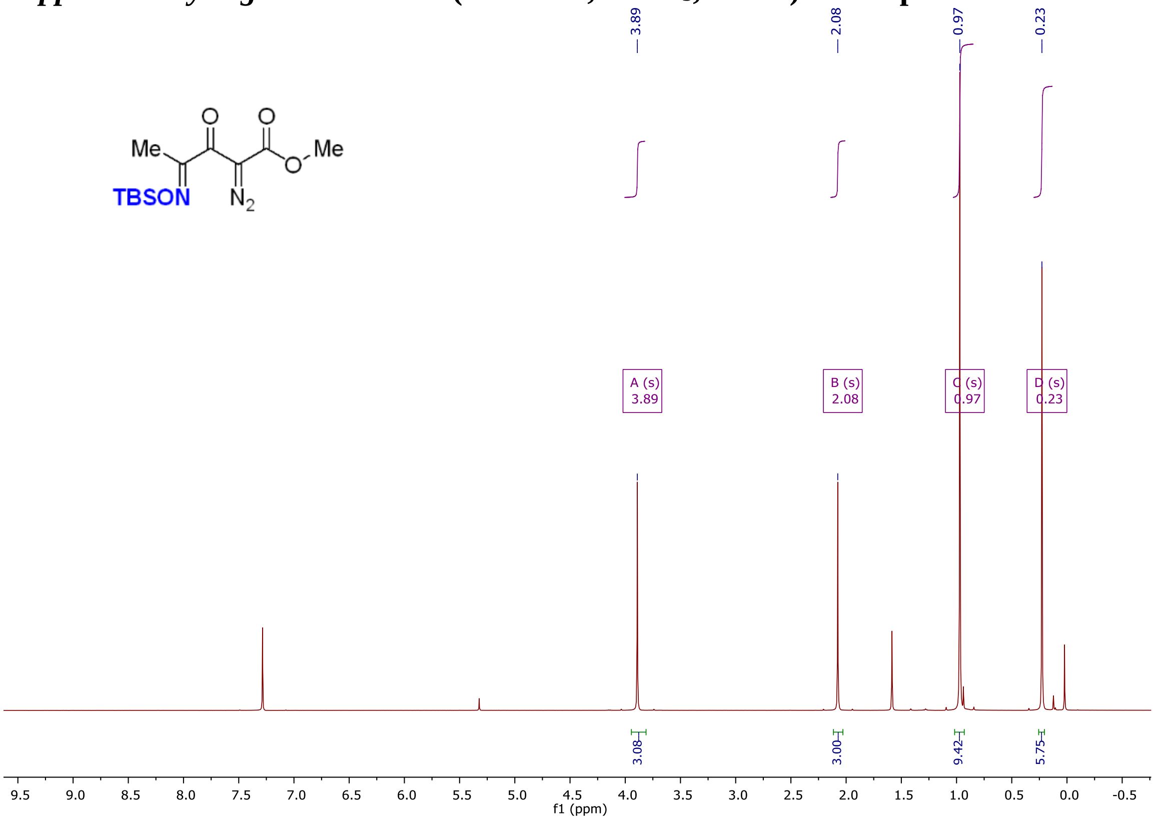

Supplementary Fig. 31. **^{13}C-NMR (126 MHz, CDCl3, 298 K) of compound 7i**

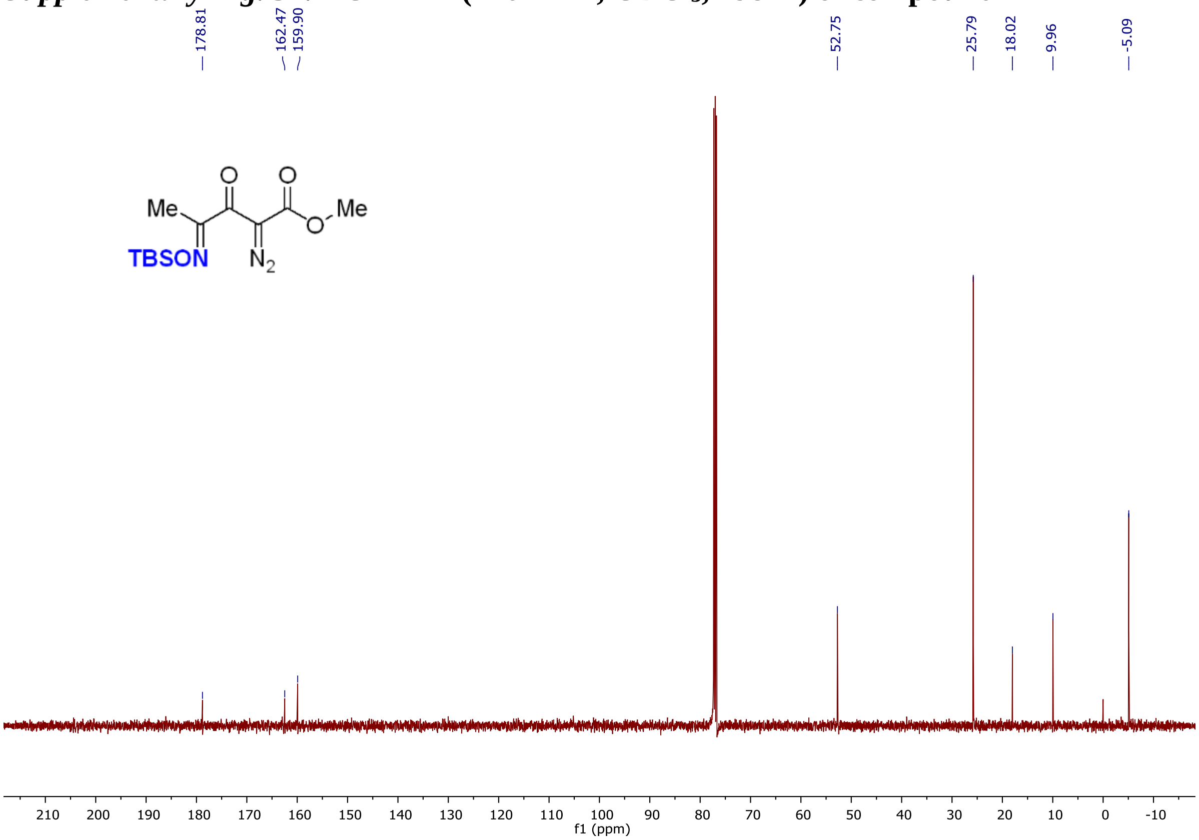

Supplementary Fig. 32. **^{1}H-NMR (500 MHz, DMSO-d6, 298 K) of compound 10c**

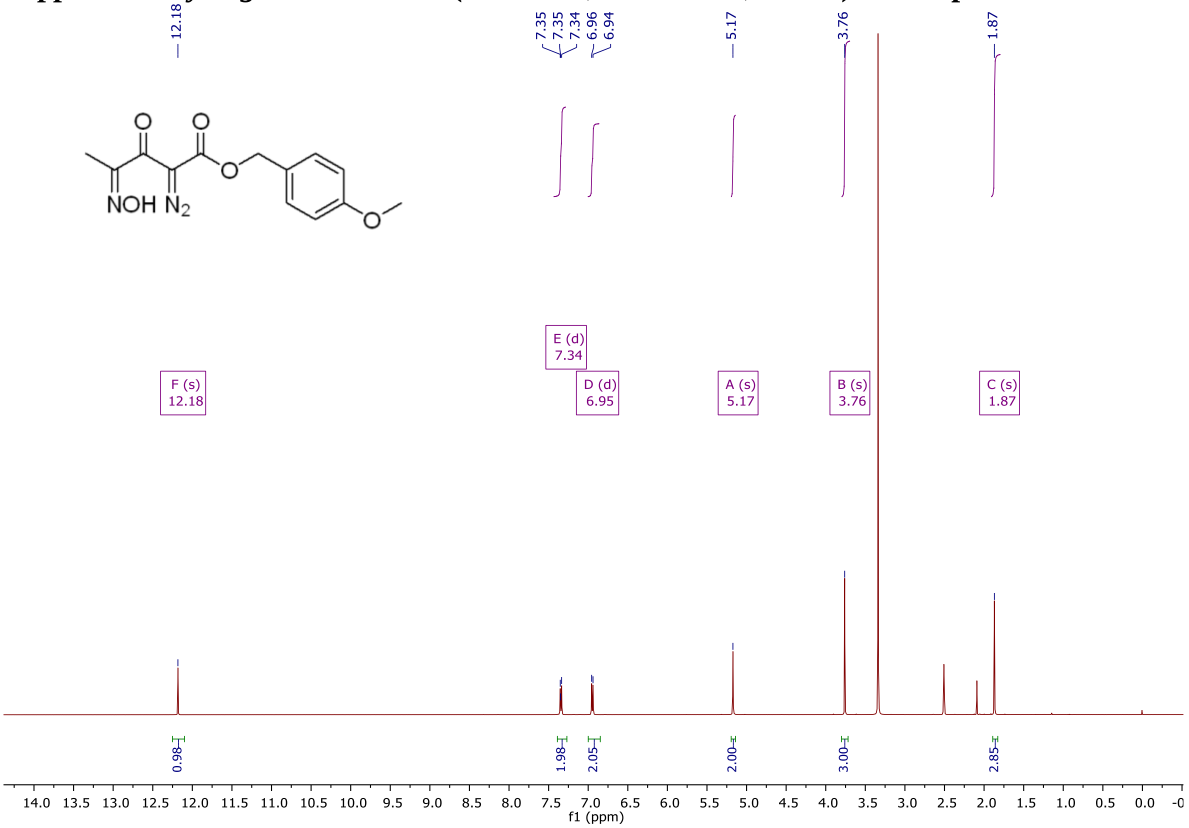

Supplementary Fig. 33. **^{13}C-NMR (126 MHz, DMSO-d6, 298 K) of compound 10c**

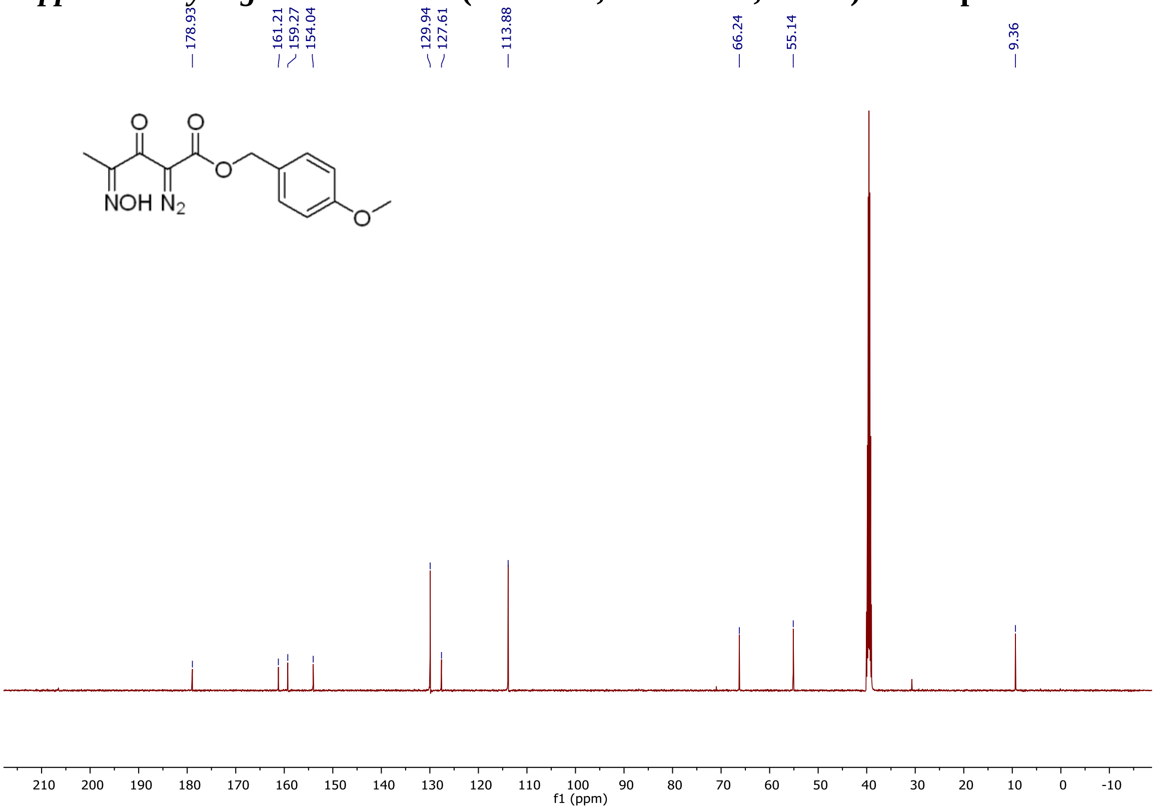

Supplementary Fig. 34. ¹H-NMR (500 MHz, DMSO-d6, 298 K) of compound 10h

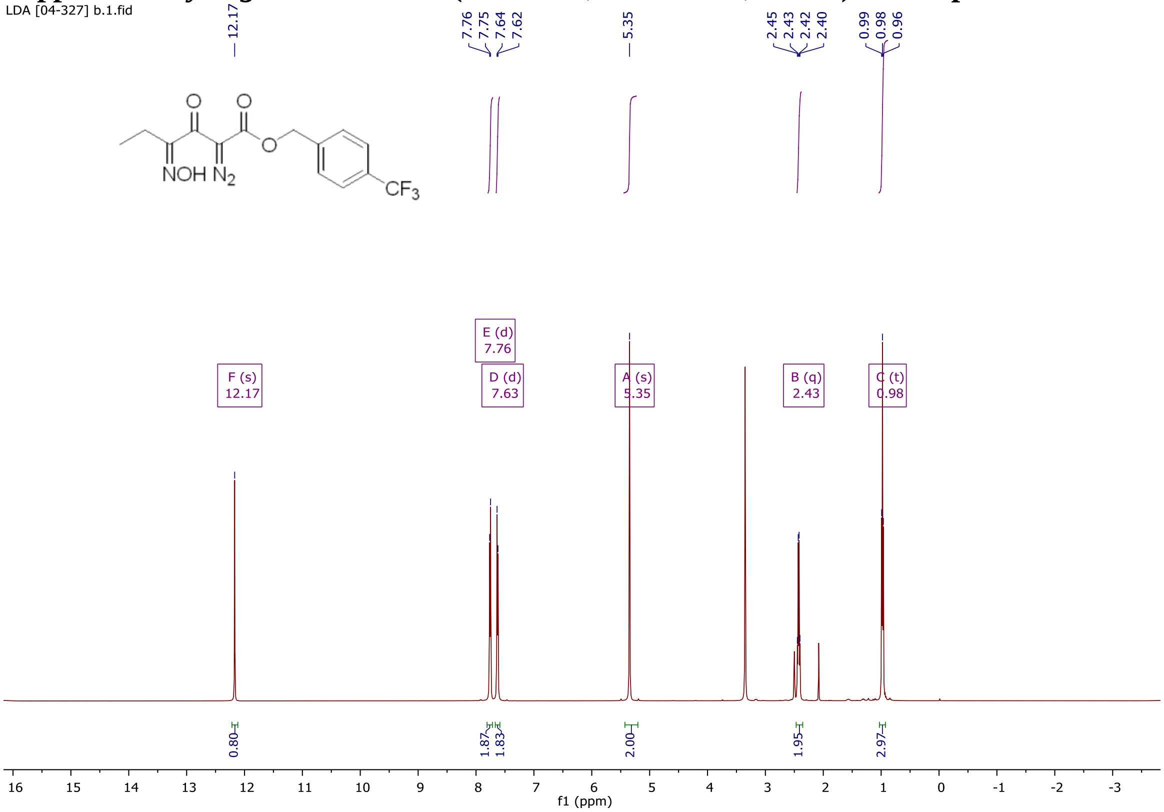

Supplementary Fig. 35. ¹³C-NMR (126 MHz, DMSO-d6, 298 K) of compound 10h

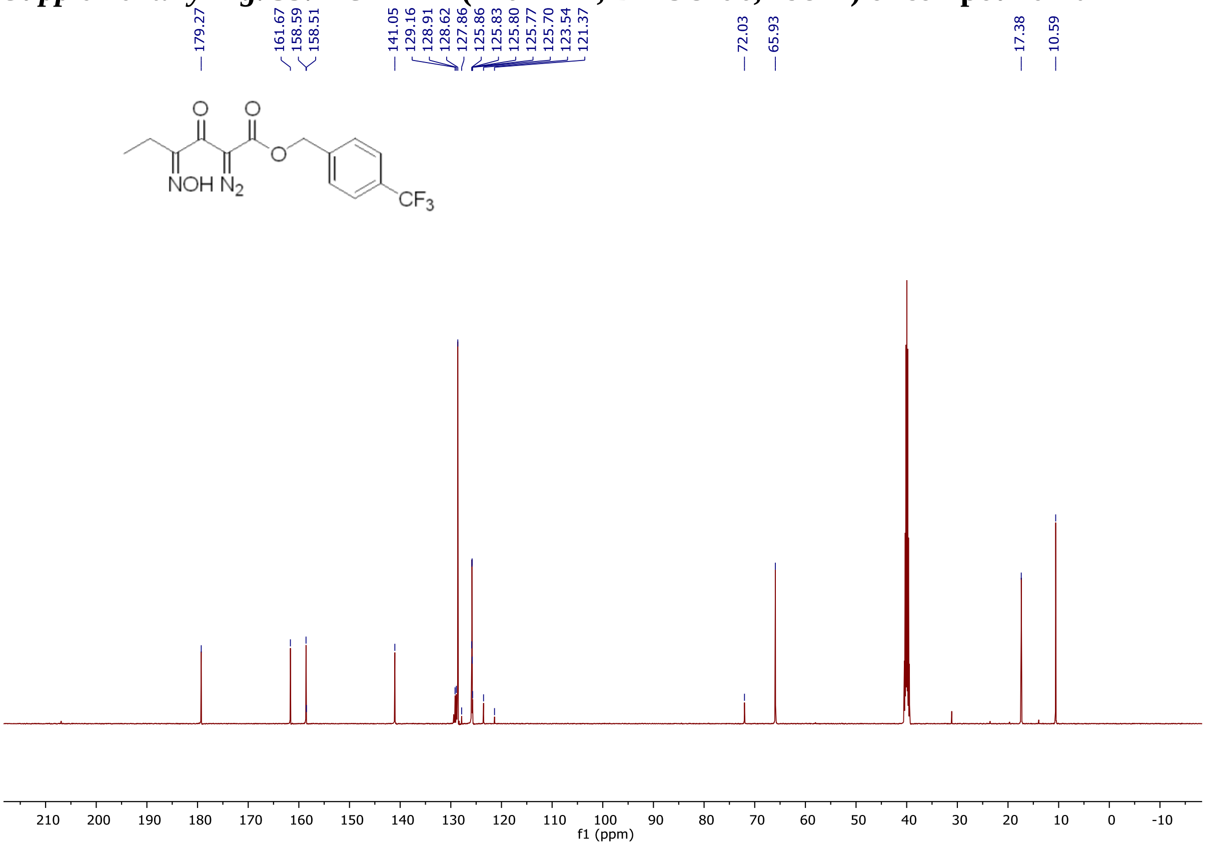

Supplementary Fig. 36. ^{1}H-NMR (500 MHz, $CDCl_3$, 298 K) of compound 11

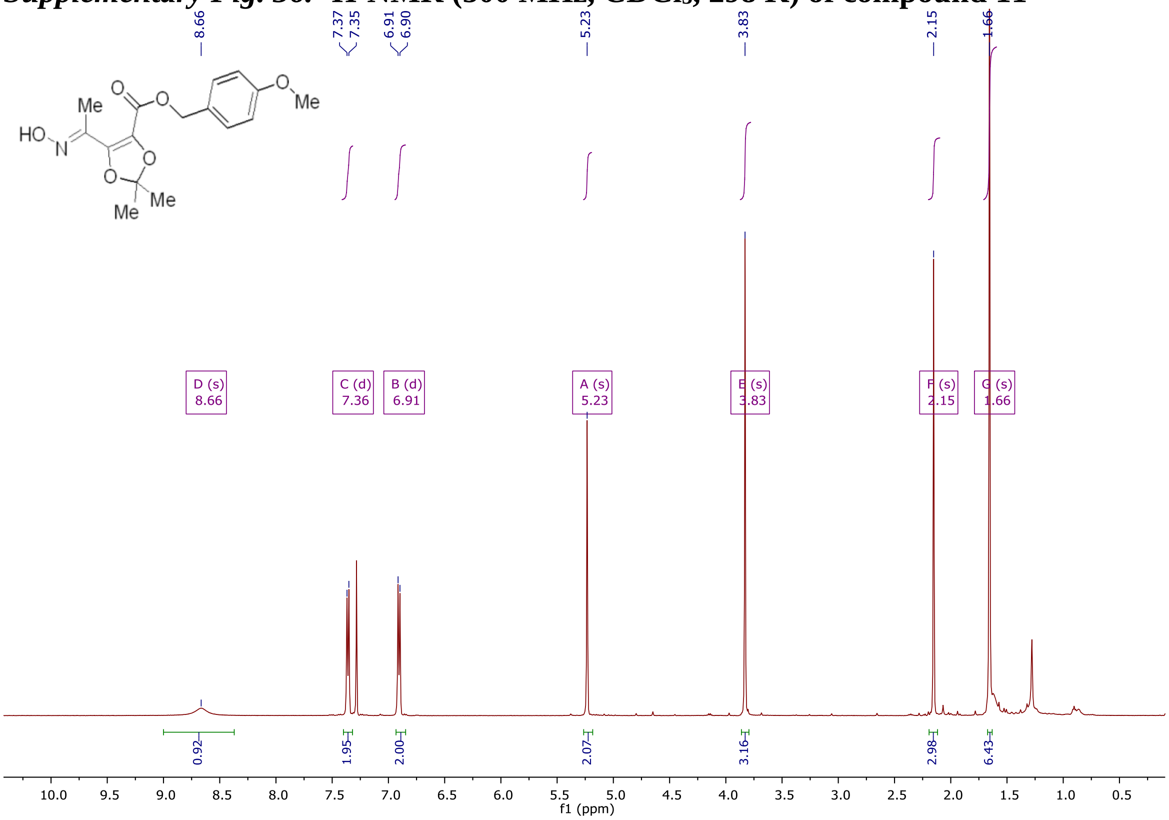

Supplementary Fig. 37. ^{13}C-NMR (126 MHz, $CDCl_3$, 298 K) of compound 11

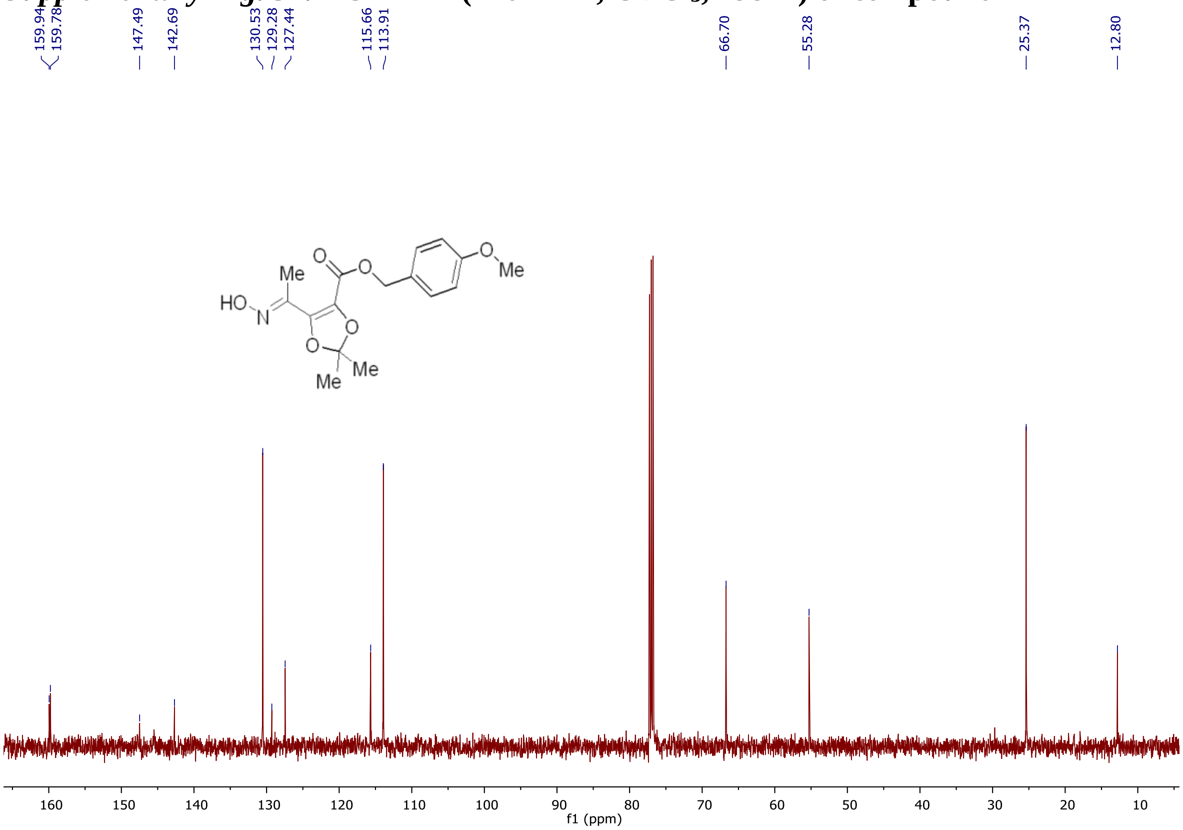

Supplementary Fig. 38. **^{1}H-NMR (500 MHz, $CDCl_3$, 298 K) of compound 8a/14a**

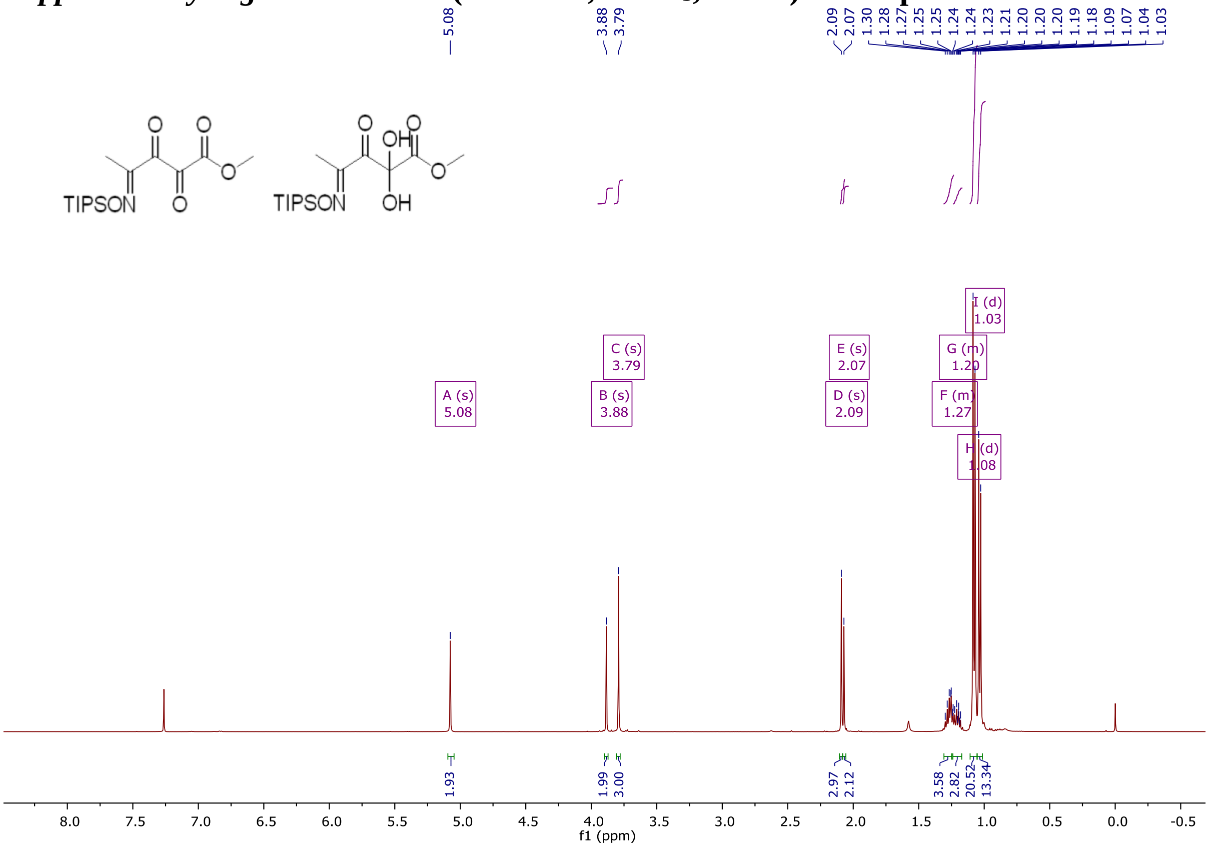

Supplementary Fig. 39. **^{13}C-NMR (126 MHz, $CDCl_3$, 298 K) of compound 8a/14a**

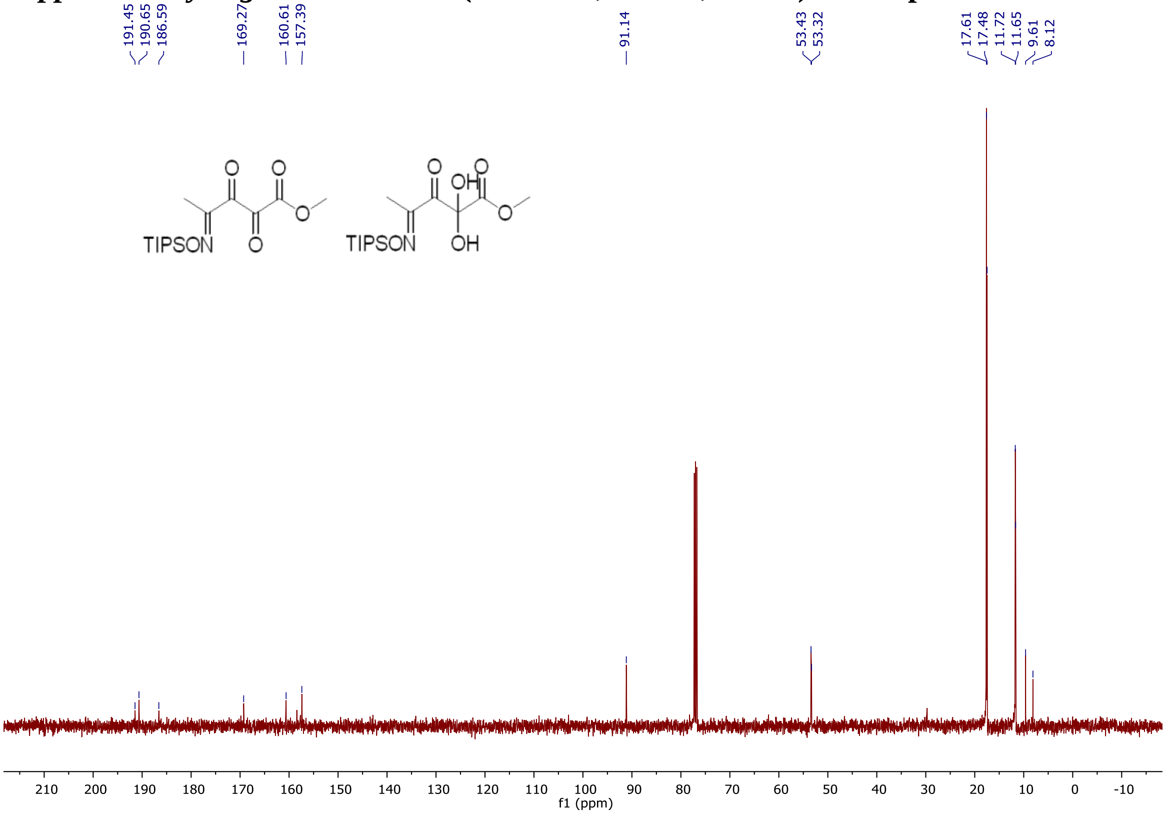

Supplementary Fig. 40. **^{15}N-NMR (50.664 MHz, CDCl$_3$, 298 K) of compound 8a/14a**

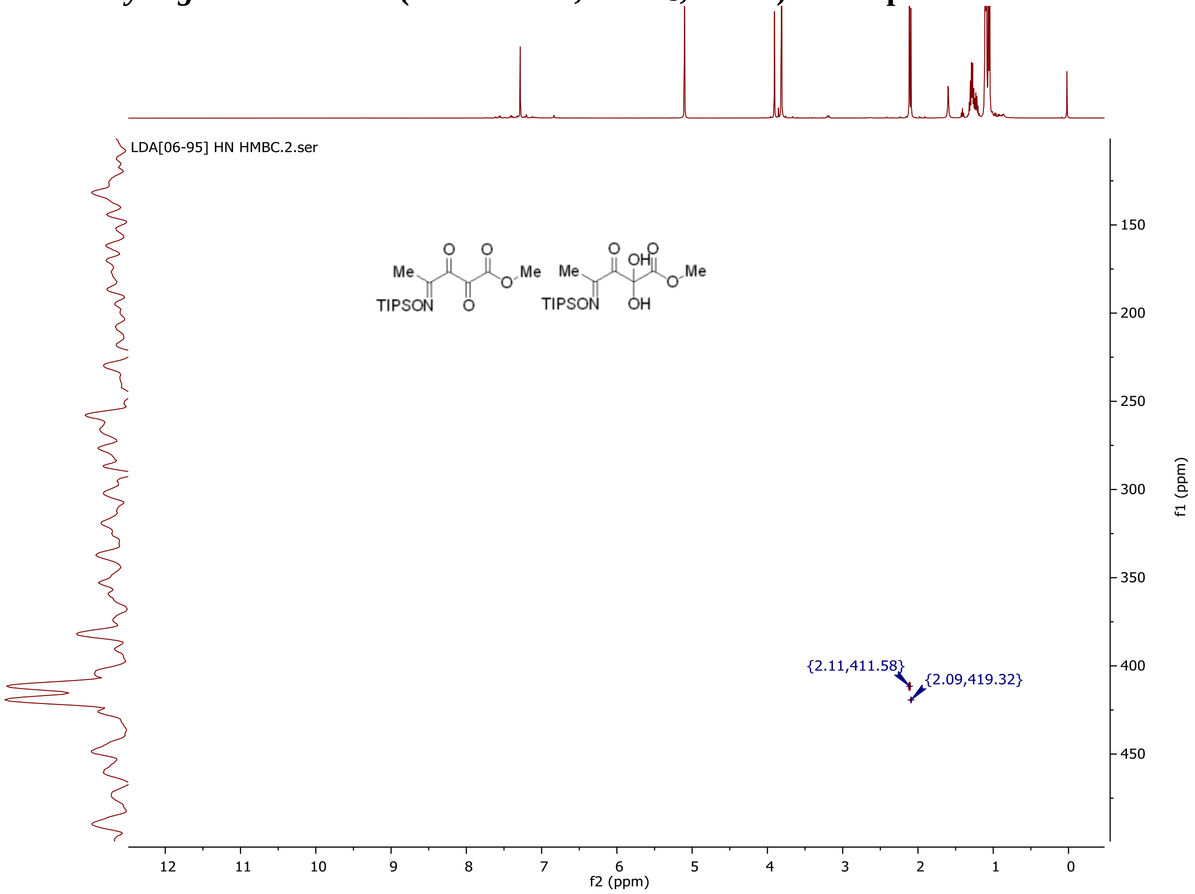

Supplementary Fig. 41. **^{1}H-NMR (500 MHz, $CDCl_3$, 298 K) of compound 8b/14b**

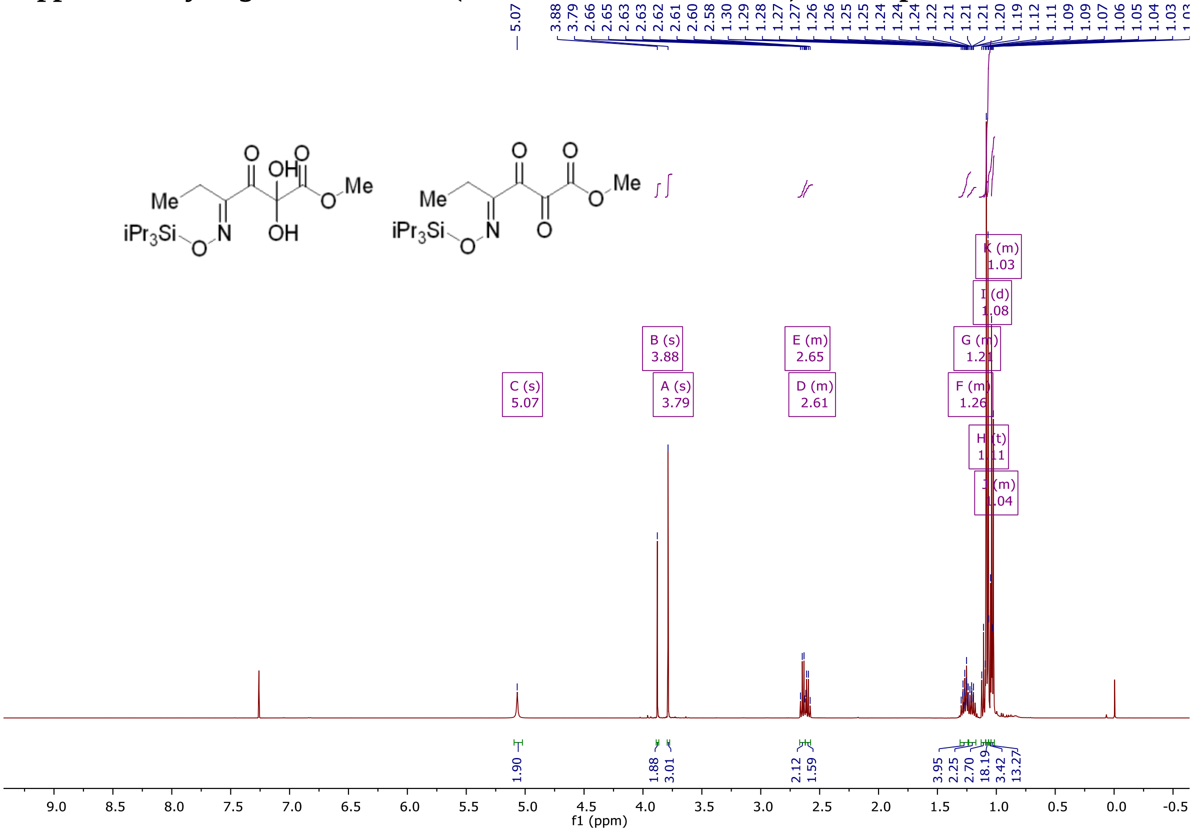

Supplementary Fig. 42. **^{13}C-NMR (126 MHz, $CDCl_3$, 298 K) of compound 8b/14b**

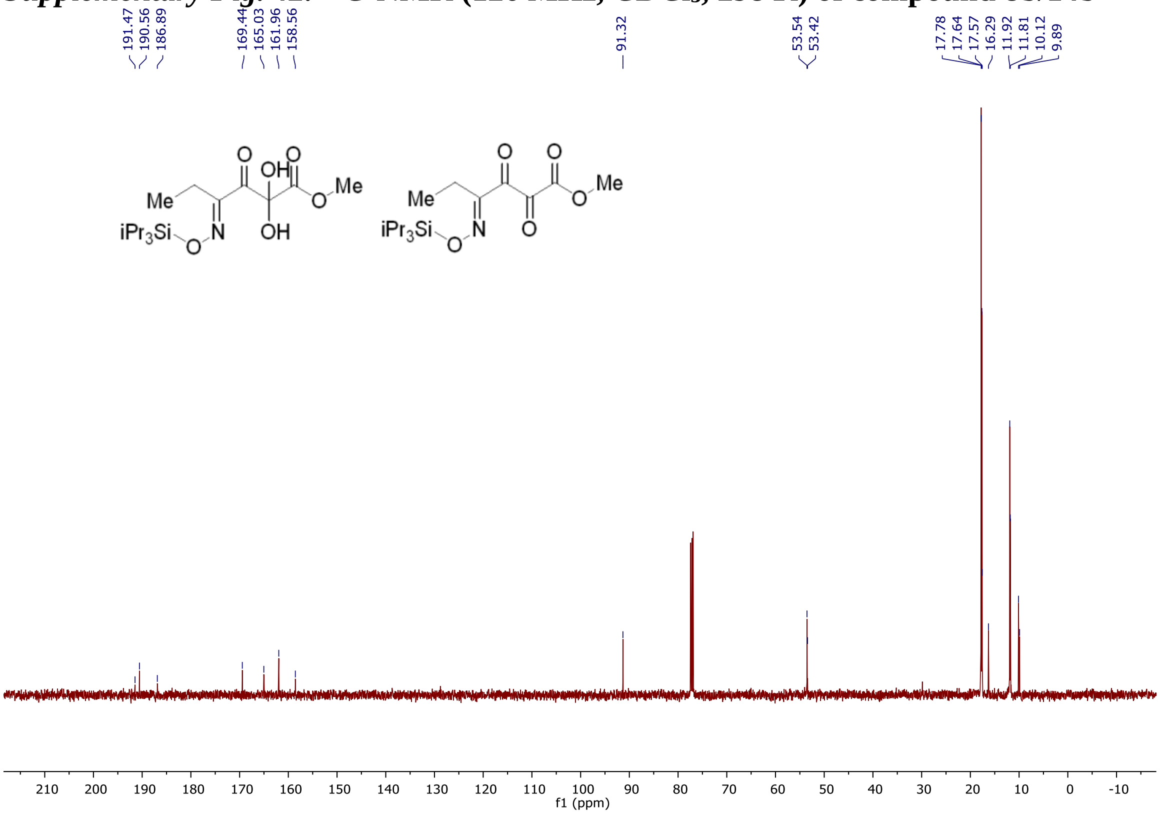

Supplementary Fig. 43. **^{1}H-NMR (500 MHz, CDCl$_3$, 298 K) of compound 8d/14d**

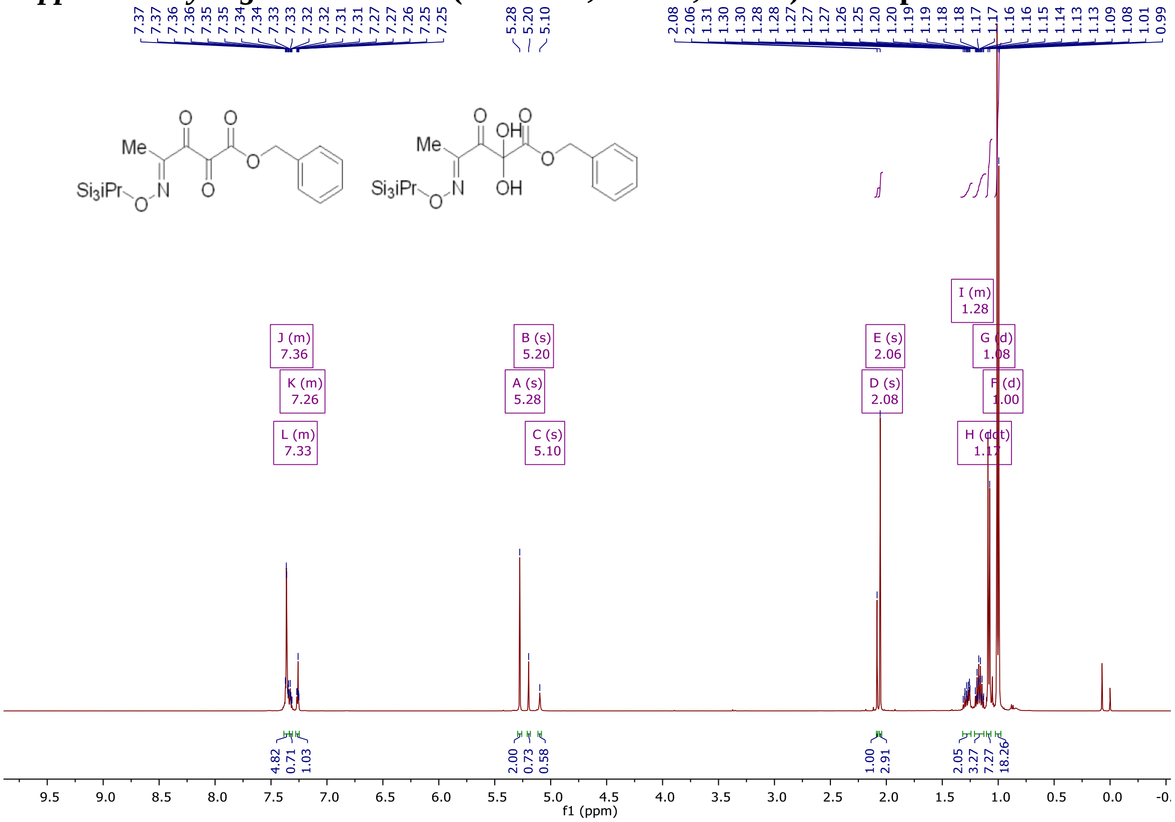

Supplementary Fig. 44. **^{13}C-NMR (126 MHz, CDCl$_3$, 298 K) of compound 8d/14d**

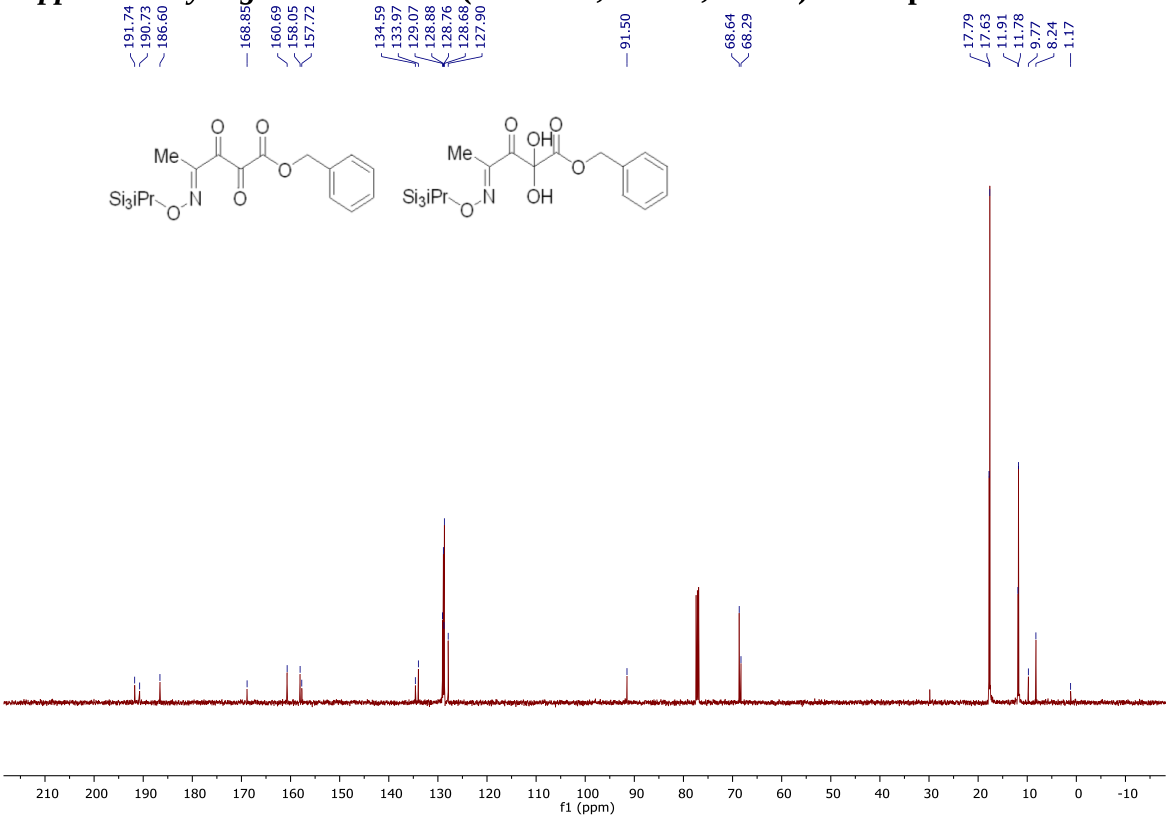

Supplementary Fig. 45. **^{1}H-NMR (500 MHz, $CDCl_3$, 298 K) of compound 8e/14e**

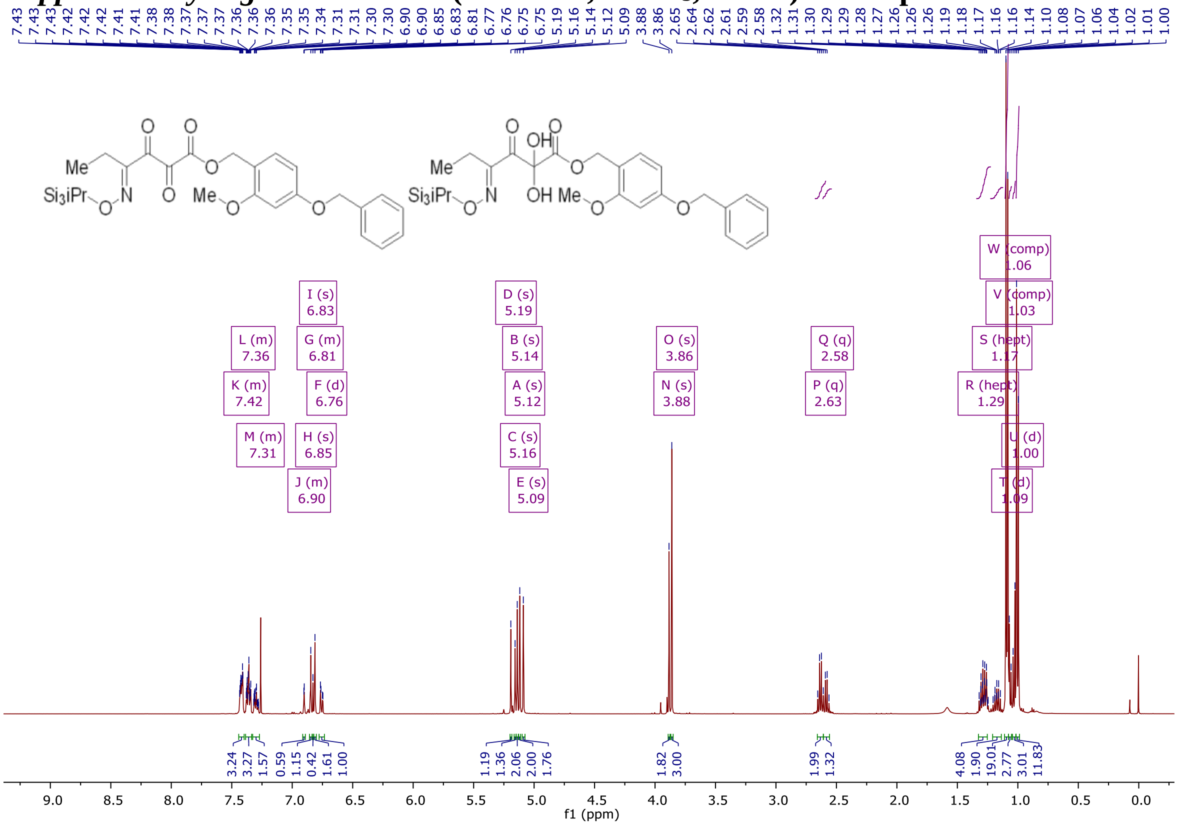

Supplementary Fig. 46. **^{13}C-NMR (126 MHz, $CDCl_3$, 298 K) of compound 8e/14e**

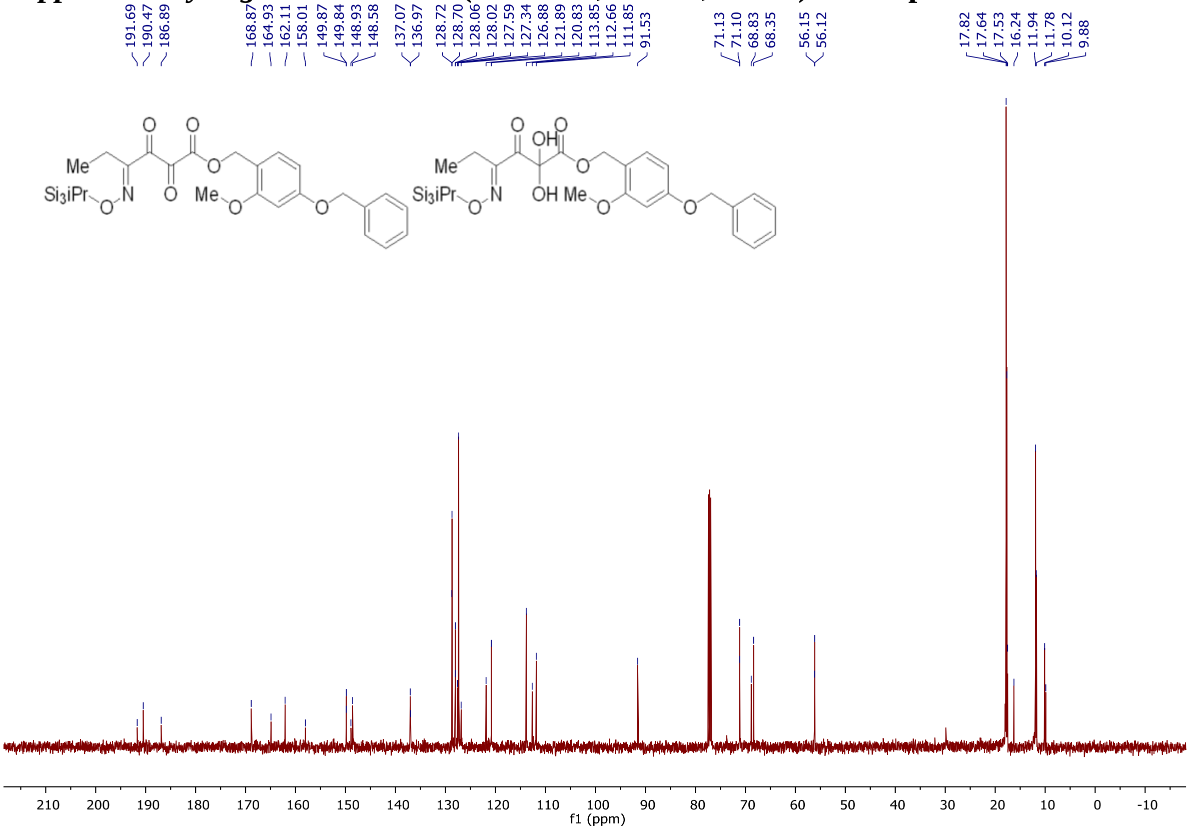

Supplementary Fig. 47. **¹H-NMR (500 MHz, CDCl₃, 298 K) of compound 8f/14f**

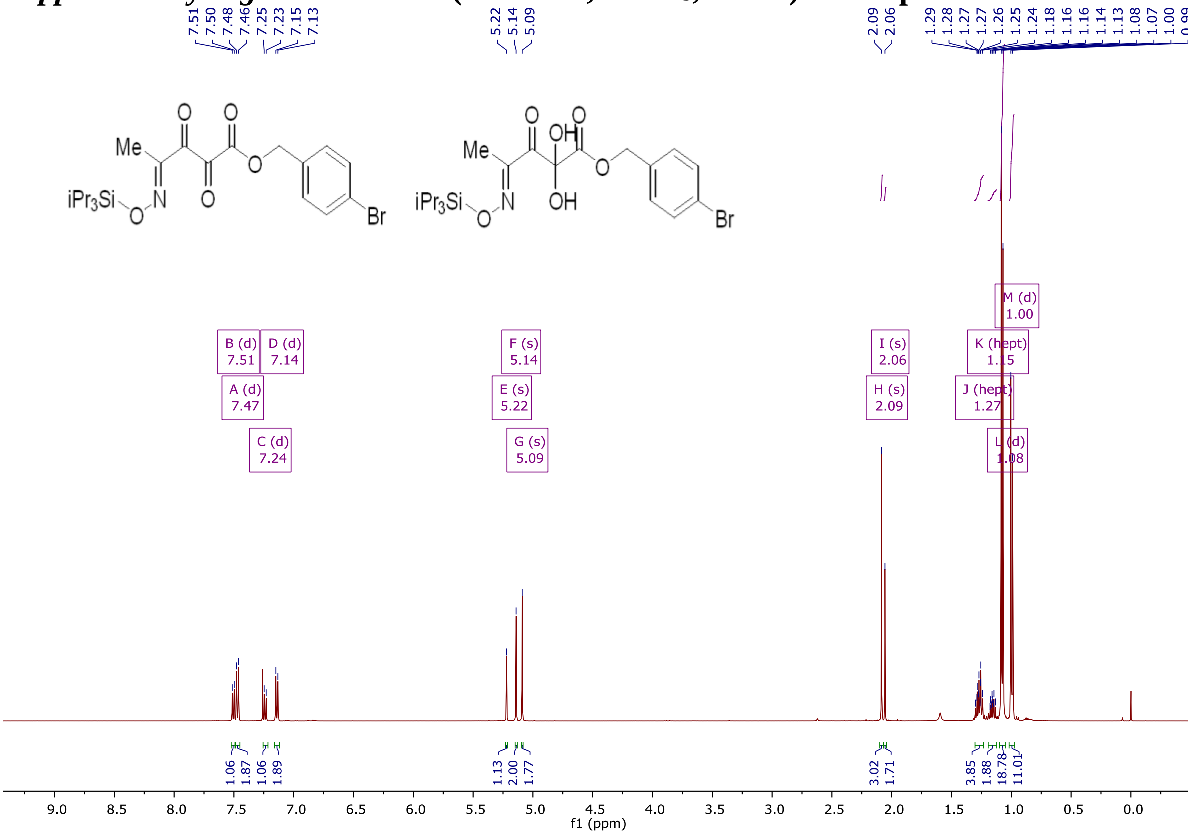

Supplementary Fig. 48. **¹³C-NMR (126 MHz, CDCl₃, 298 K) of compound 8f/14f**

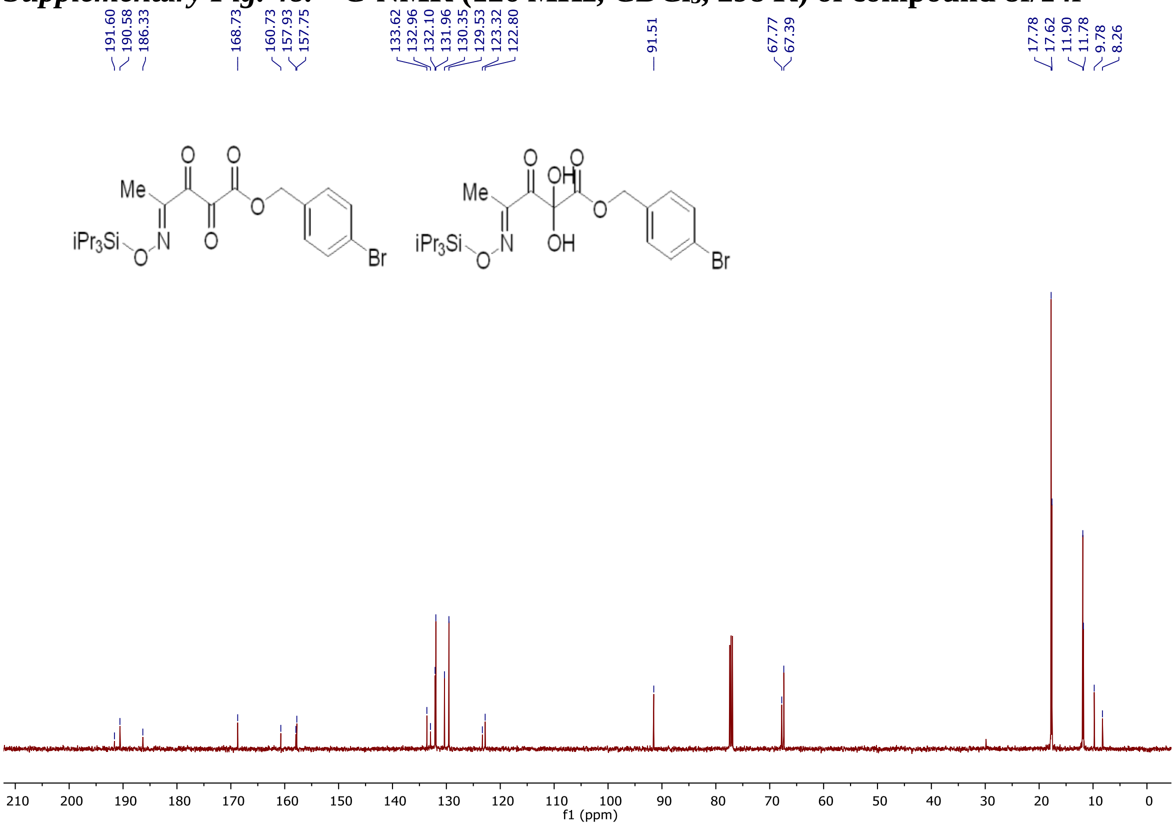

Supplementary Fig. 49. **^{1}H-NMR (500 MHz, $CDCl_3$, 298 K) of compound 8g/14g**

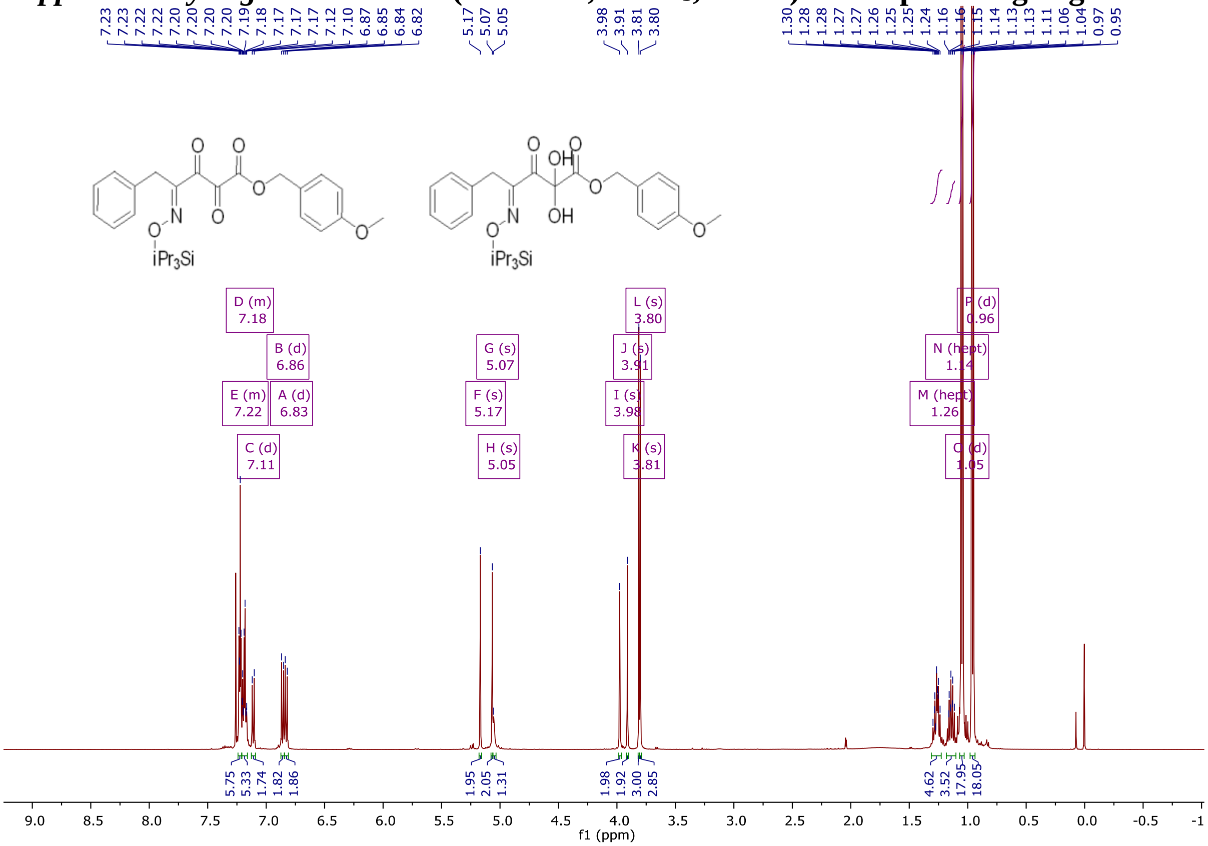

Supplementary Fig. 50. **^{13}C-NMR (126 MHz, $CDCl_3$, 298 K) of compound 8g/14g**

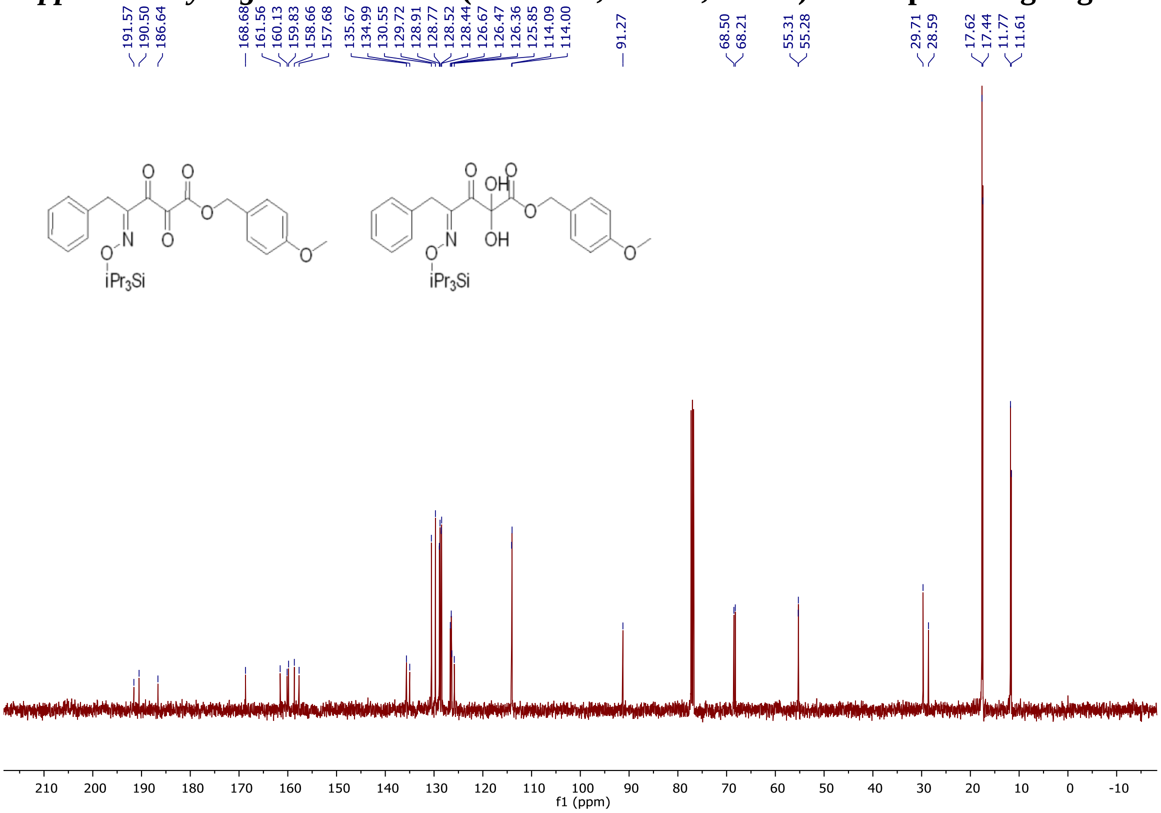

Supplementary Fig. 51. **^{1}H-NMR (500 MHz, $CDCl_3$, 298 K) of compound 8h/14h**

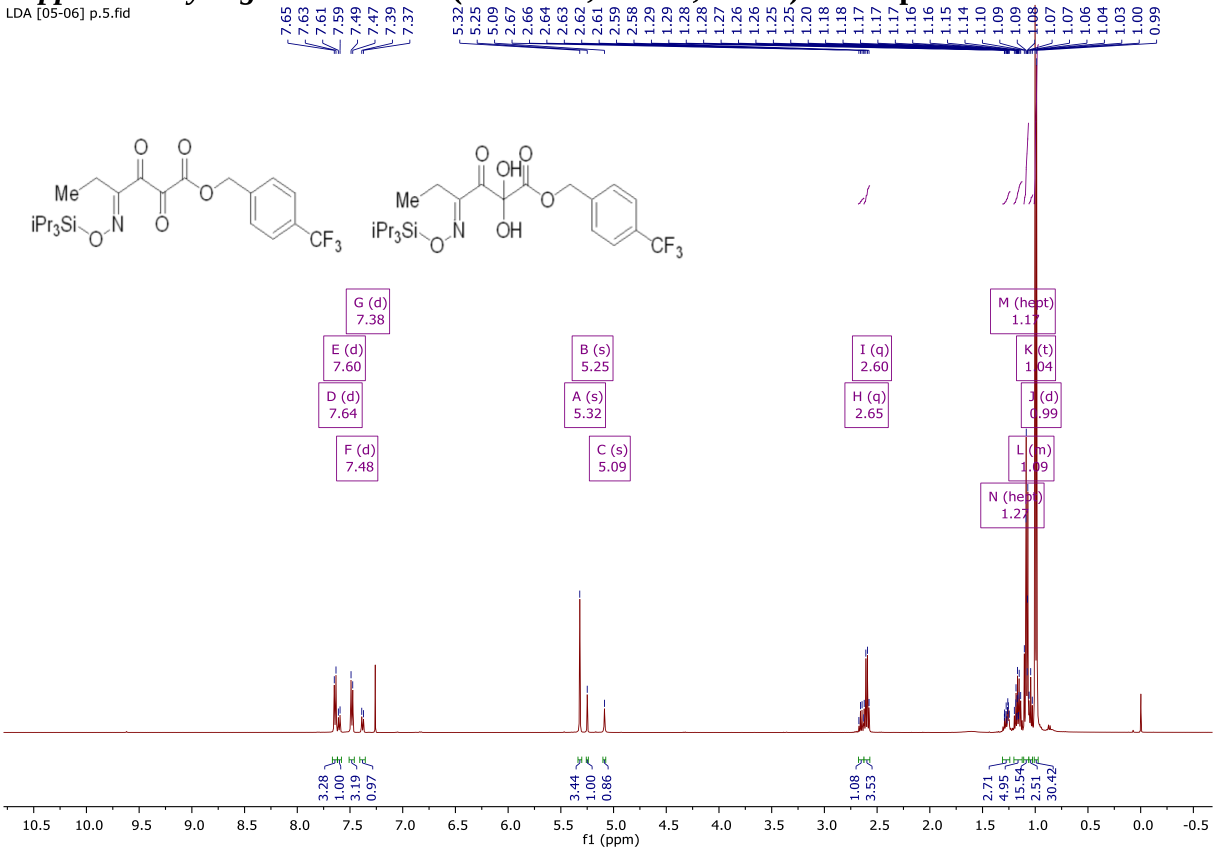

Supplementary Fig. 52. **^{13}C-NMR (126 MHz, $CDCl_3$, 298 K) of compound 8h/14h**

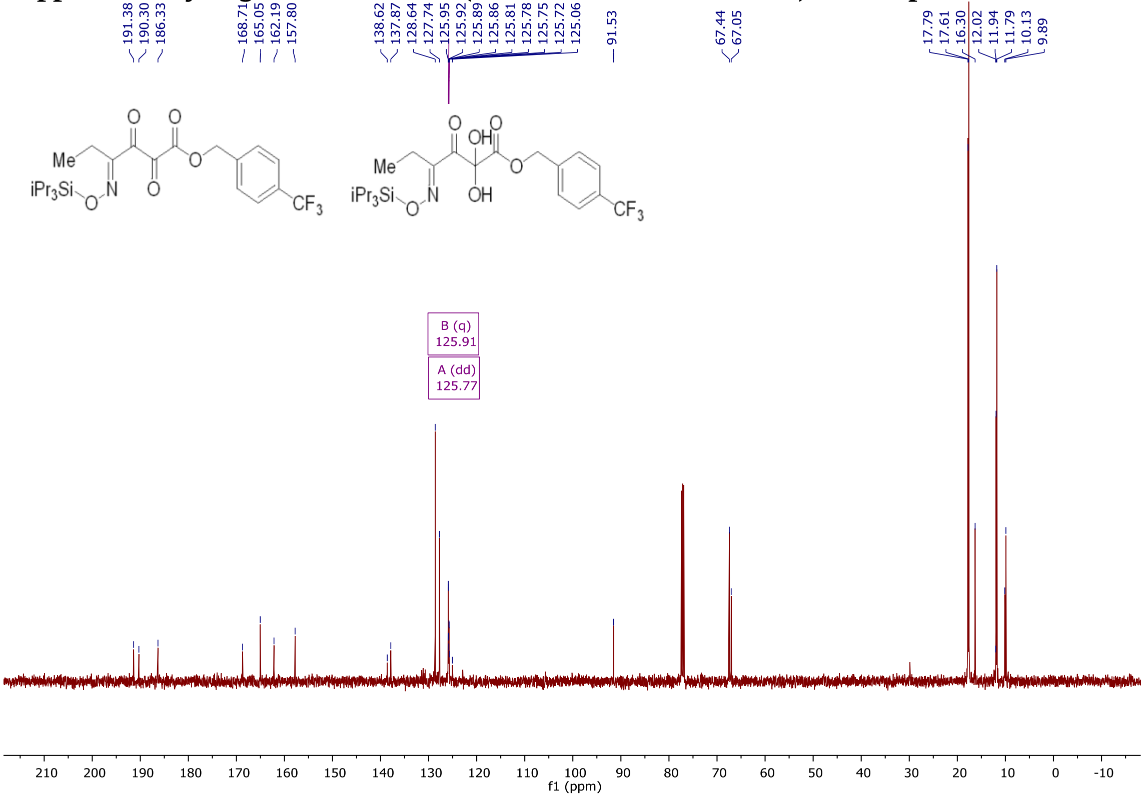

Supplementary Fig. 53. **^{1}H-NMR (500 MHz, $CDCl_3$, 298 K) of compound 8k/14k**

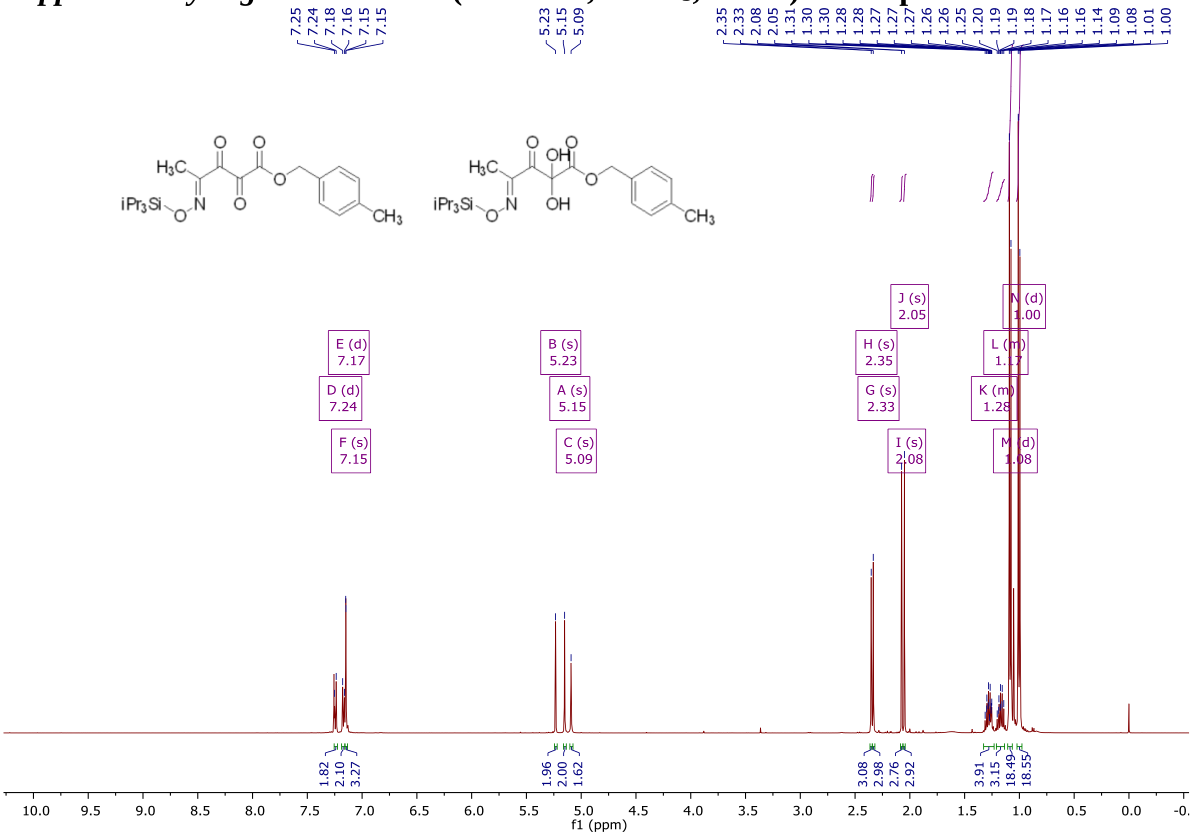

Supplementary Fig. 54. **^{13}C-NMR (126 MHz, $CDCl_3$, 298 K) of compound 8k/14k**

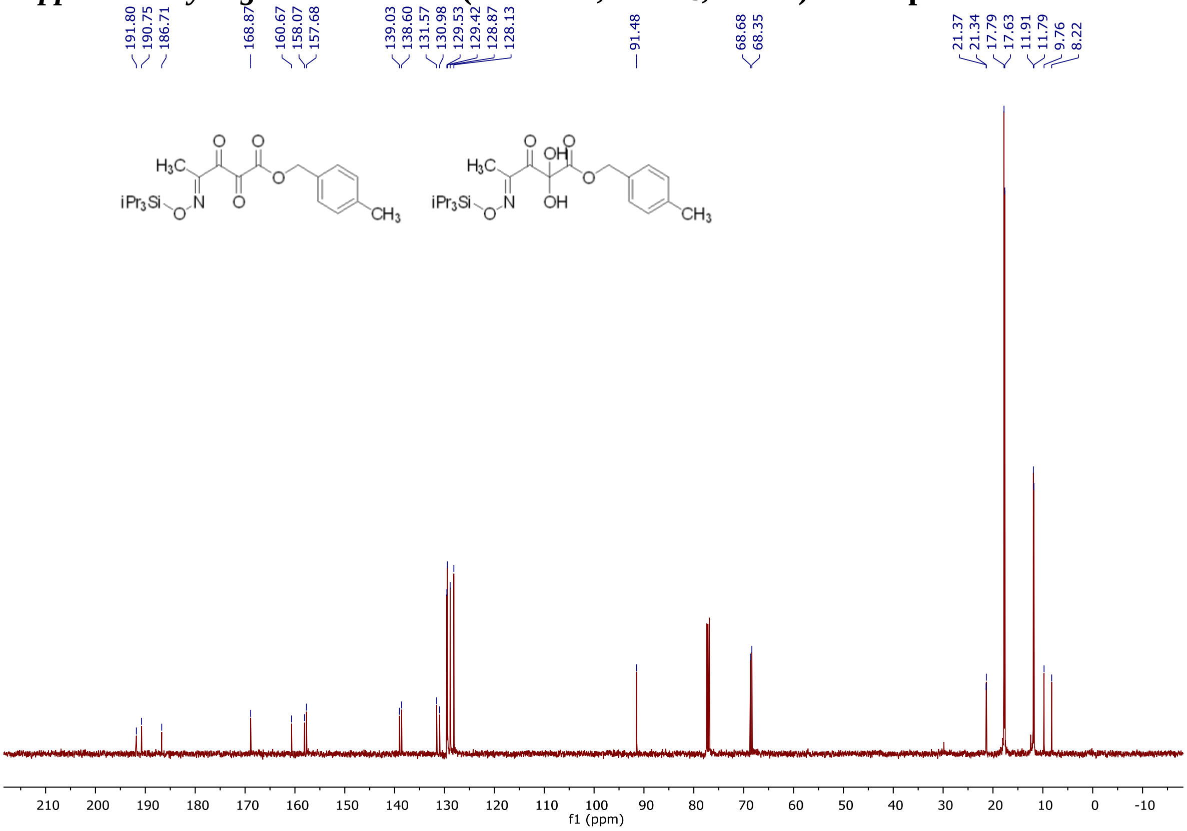

Supplementary Fig. 55. ^{1}H-NMR (500 MHz, acetonitrile-d_3, 298 K) of compound 13a

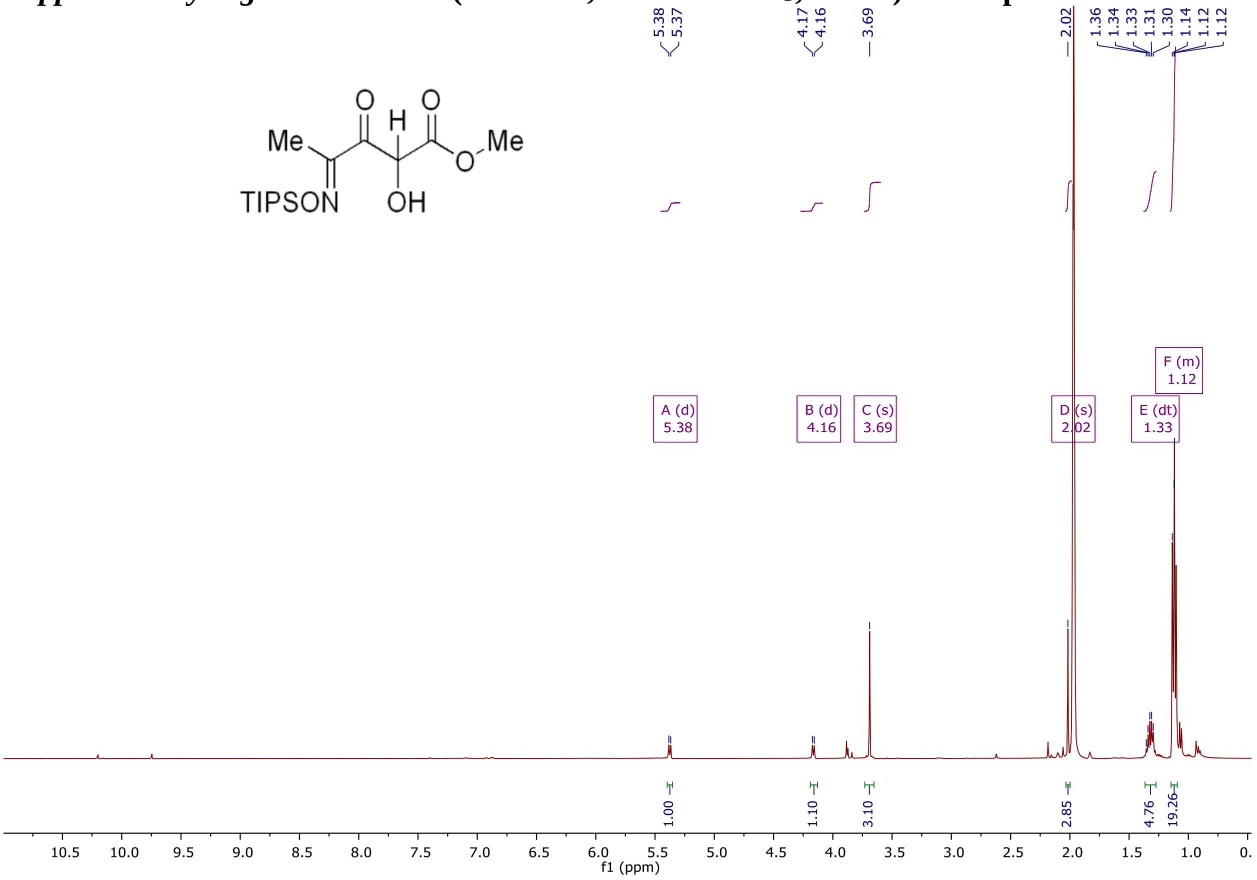

Supplementary Fig. 56. ^{13}C-NMR (126 MHz, acetonitrile-d_3, 298 K) of compound 13a

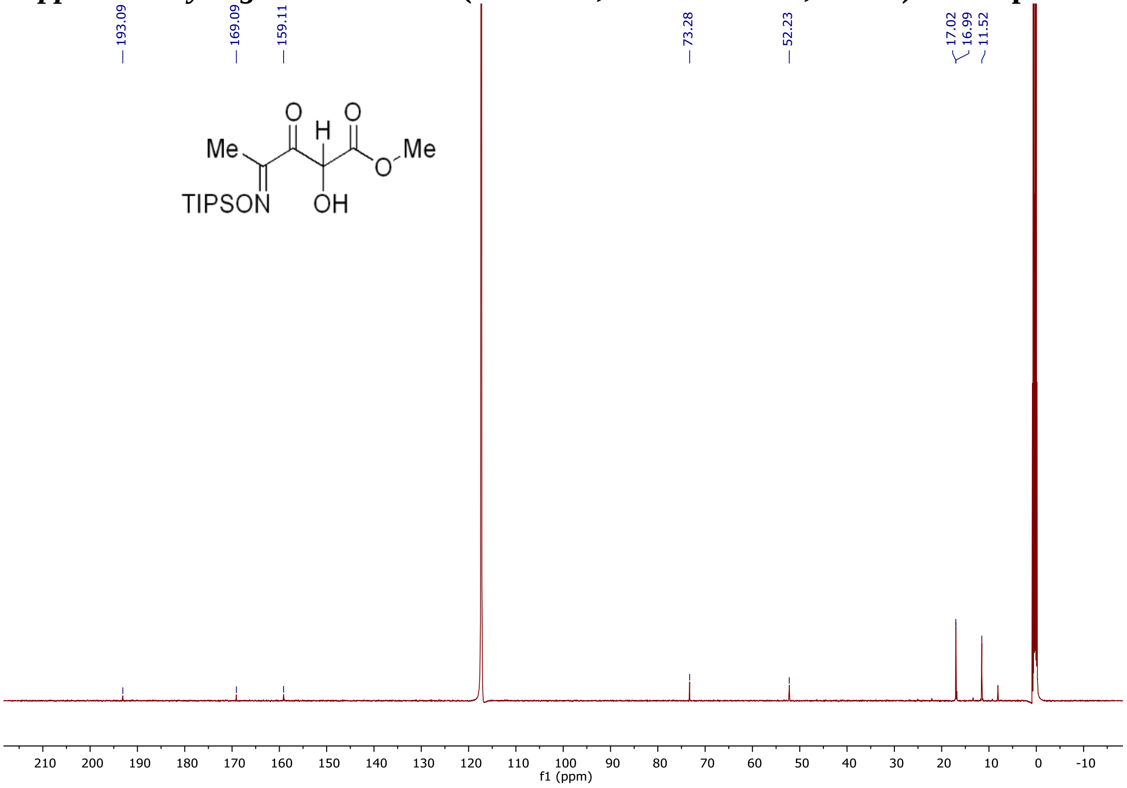

Supplementary Fig. 57. **^{1}H-NMR (500 MHz, $CDCl_3$, 298 K) of compound 12a**

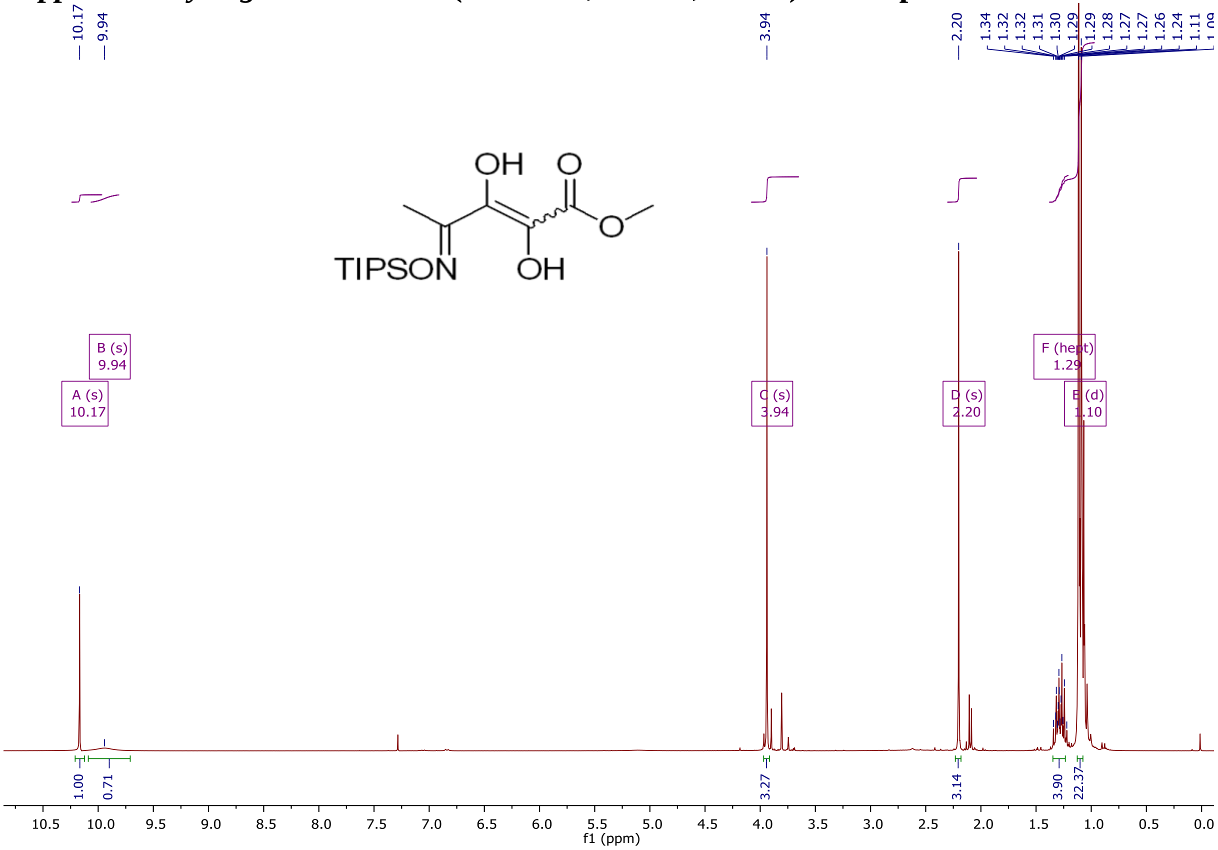

Supplementary Fig. 58. **^{13}C-NMR (126 MHz, $CDCl_3$, 298 K) of compound 12a**

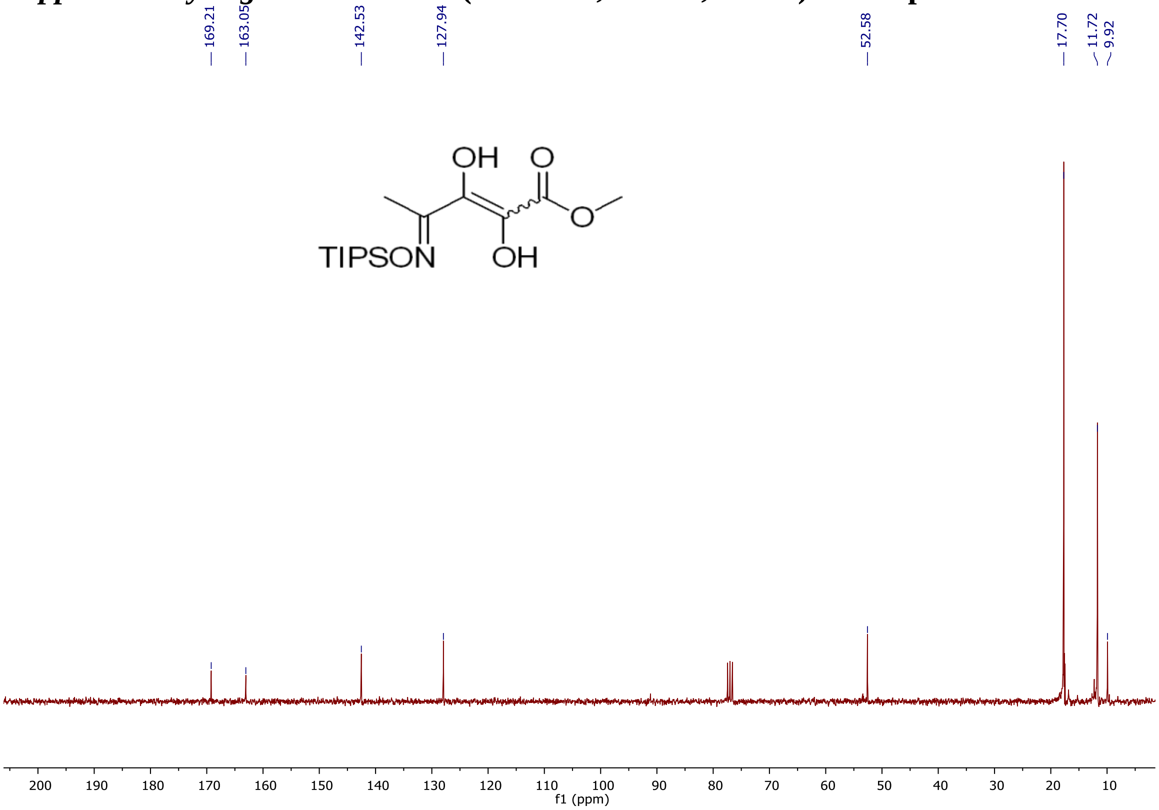

3 Supplementary References

1. (a) Xu, X., Wang, X., Zavalij, P. V. & Doyle, P. M. Straightforward Access to the [3.2.2]Nonatriene Structural Framework via Intramolecular Cyclopropenation/Buchner Reaction/Cope Rearrangement Cascade. *Org. Lett.* **17**, 790-793 (2015). (b) Deng, Y., Massey, L. A., Rodriguez Núñez, Y. A., Arman, H. & Doyle, M. P. Catalytic Divergent [3+3]- and [3+2]-Cycloaddition by Discrimination Between Diazo Compounds. *Angew. Chem. Int. Ed.* **57**, 12292-12296 (2017). (c) Dong, K., Marichev, K. O., Xu, X. & Doyle, M. P. High Stereocontrol in the Preparation of Silyl-Protected γ-Substituted Enoldiazoacetates. *Synlett* **30**, 1457-1461 (2019).
2. CrysAlisPro 1.171.40.63a (Rigaku Oxford Diffraction) (2019).
3. SCALE3 ABSPACK -An Oxford Diffraction program (1.0.4,gui:1.0.3) (C) Oxford Diffraction Ltd. (2005).
4. O. V. Dolomanov, L. J. Bourhis, R. J. Gildea, J. A. K. Howard & H. Puschmann, OLEX2: a complete structure solution, refinement and analysis program. *J. Appl. Cryst*. **42**, 339-341 (2009).
5. Sheldrick, G. M. Crystal structure refinement with SHELXL. *Acta Cryst*. **71**, 3-8 (2015).
6. Sheldrick, G. M. A short history of SHELX. *Acta Cryst.* **64**, 112-122 (2008).
7. M. J. Frisch, et al., Gaussian 16, Revision B.01, Gaussian, Inc., Wallingford CT (2016).
8. (a) Becke, A. D. Density-functional exchange-energy approximation with correct asymptotic behavior. *Phys. Rev. A* **38**, 3098-3100 (1988). (b) Perdew, J. P. Density-functional approximation for the correlation energy of the inhomogeneous electron gas. *Phys. Rev. B* **33**, 8822-8824 (1986).
9. Grimme, S., Antony, J., Ehrlich, S. & Krieg, H. A consistent and accurate *ab initio* parametrization of density functional dispersion correction (DFT-D) for the 94 elements H-Pu. *J. Chem. Phys.* **132**, 154104 (2010).
10. Wadt, W. R. & Hay, P. J. *Ab initio* effective core potentials for molecular calculations. Potentials for main group elements Na to Bi. *J. Chem. Phys.* **82**, 284-298 (1985).
11. (a) Fukui, K. Formulation of the reaction coordinate. *J. Phys. Chem.* **74**, 4161-4163 (1970). (b) Fukui, K. The path of chemical reactions - the IRC approach. *Acc. Chem. Res.* **14**, 363-368 (1981).
12. (a) Spitznagel, G. W., Clark, T., Schleyer, P. von R. & Hehre, W. J. An evaluation of the performance of diffuse function-augmented basis sets for second row elements, Na-Cl. *J. Comput. Chem.* **8**, 1109-1116 (1987). (b) Clark, T., Chandrasekhar, J., Spitznagel, G. W. & von Ragué Schleyer, P. Efficient diffuse function-augmented basis sets for anion calculations. III. The 3-21+G basis set for first-row elements, Li-F. *J. Comput. Chem.* **4**, 294-301 (1983). (c) Francl, M. M., Pietro, W. J., Hehre, W. J., Binkley, J. S., Gordon, M. S., DeFrees, D. J. & Pople, J. A. Self-consistent molecular orbital methods. XXIII. A polarization-type basis set for second-row elements. *J. Chem. Phys.* **77**, 3654-3665 (1982). (d) Krishnan, R., Binkley, J. S., Seeger, R. & Pople, J. A. Self-consistent molecular orbital methods. XX. A basis set for correlated wave functions. *J. Chem. Phys.* **72**, 650-654 (1980). (e) McLean, A. D. & Chandler, G. S. Contracted Gaussian basis sets for molecular calculations. I. Second row atoms, Z=11-18. *J. Chem. Phys.* **72**, 5639-5648 (1980).
13. Marenich, A. V., Cramer, C. J. & Truhlar, D. G. Universal Solvation Model Based on Solute Electron Density and on a Continuum Model of the Solvent Defined by the Bulk Dielectric Constant and Atomic Surface Tensions. *J. Phys. Chem. B* **113**, 6378-6396 (2009).
14. Legault, C. Y. CYLview20, Université de Sherbrooke, (http://www.cylview.org) (2020).
