# Supplementary material for: Polyfunctionalization of vicinal carbon centers and synthesis of unsymmetric 1,2,3,4-tetracarbonyl compounds
Source: Nat Commun. 2023 Feb 27;14:1109. doi: 10.1038/s41467-023-36757-w (PMC9971237; doi:10.1038/s41467-023-36757-w)
Supplement: Supplementary file 2 — Description of Additional Supplementary Files [file 41467_2023_36757_MOESM2_ESM.docx]

**Description of Additional Supplementary Files**

**Supplementary Data 1. Stationary points.** Cartesian coordinates of the structures.
